# Transcriptional Programming of Normal and Inflamed Human Epidermis at Single-Cell Resolution

**DOI:** 10.1016/j.celrep.2018.09.006

**Published:** 2018-10-23

**Authors:** Jeffrey B. Cheng, Andrew J. Sedgewick, Alex I. Finnegan, Paymann Harirchian, Jerry Lee, Sunjong Kwon, Marlys S. Fassett, Justin Golovato, Matthew Gray, Ruby Ghadially, Wilson Liao, Bethany E. Perez White, Theodora M. Mauro, Thaddeus Mully, Esther A. Kim, Hani Sbitany, Isaac M. Neuhaus, Roy C. Grekin, Siegrid S. Yu, Joe W. Gray, Elizabeth Purdom, Ralf Paus, Charles J. Vaske, Stephen C. Benz, Jun S. Song, Raymond J. Cho

**Affiliations:** 1 Department of Dermatology, University of California, San Francisco and Veterans Affairs Medical Center, San Francisco, CA, USA; 2 Nantomics, LLC, Culver City, CA, USA; 3 Department of Physics, Carl R. Woese Institute of Genomic Biology, University of Illinois at Urbana-Champaign, Champaign, IL, USA; 4 Department of Biomedical Engineering, OHSU Center for Spatial Systems Biomedicine, Portland, OR, USA; 5 Department of Dermatology, University of California, San Francisco, San Francisco, CA, USA; 6 Department of Dermatology and Skin Tissue Engineering Core, Northwestern University, Chicago, IL, USA; 7 Department of Pathology, University of California, San Francisco, San Francisco, CA, USA; 8 Department of Plastic Surgery, University of California, San Francisco, San Francisco, CA, USA; 9 Department of Statistics, University of California, Berkeley, Berkeley, CA, USA; 10 Centre for Dermatology Research, University of Manchester, Manchester Academic Health Science Centre and NIHR Manchester Biomedical Research Centre, Manchester, UK; 11 Department of Dermatology and Cutaneous Surgery, University of Miami Miller School of Medicine, Miami, FL, USA; 12 These authors contributed equally; 13 Senior author; 14 Lead Contact

## Abstract

Perturbations in the transcriptional programs specifying epidermal differentiation cause diverse skin pathologies ranging from impaired barrier function to inflammatory skin disease. However, the global scope and organization of this complex cellular program remain undefined. Here we report single-cell RNA sequencing profiles of 92,889 human epidermal cells from 9 normal and 3 inflamed skin samples. Transcriptomics-derived keratinocyte subpopulations reflect classic epidermal strata but also sharply compartmentalize epithelial functions such as cell-cell communication, inflammation, and *WNT* pathway modulation. In keratinocytes, ~12% of assessed transcript expression varies in coordinate patterns, revealing undescribed gene expression programs governing epidermal homeostasis. We also identify molecular fingerprints of inflammatory skin states, including *S100* activation in the interfollicular epidermis of normal scalp, enrichment of a *CD1C*^+^*CD301A*^+^ myeloid dendritic cell population in psoriatic epidermis, and *IL1β*^hi^ *CCL3*^hi^*CD14*^+^ monocyte-derived macrophages enriched in foreskin. This compendium of RNA profiles provides a critical step toward elucidating epidermal diseases of development, differentiation, and inflammation.

## In Brief

Cheng et al. report single-cell RNA sequencing of normal and inflamed human epidermis, revealing a discrete set of specialized keratinocytes that exhibit a distinct composition at different anatomic sites. Myeloid dendritic cells and macrophages also vary sharply with epidermal anatomic site and inflammation, indicating dynamic programming of antigen-presenting cells.

## Graphical Abstract

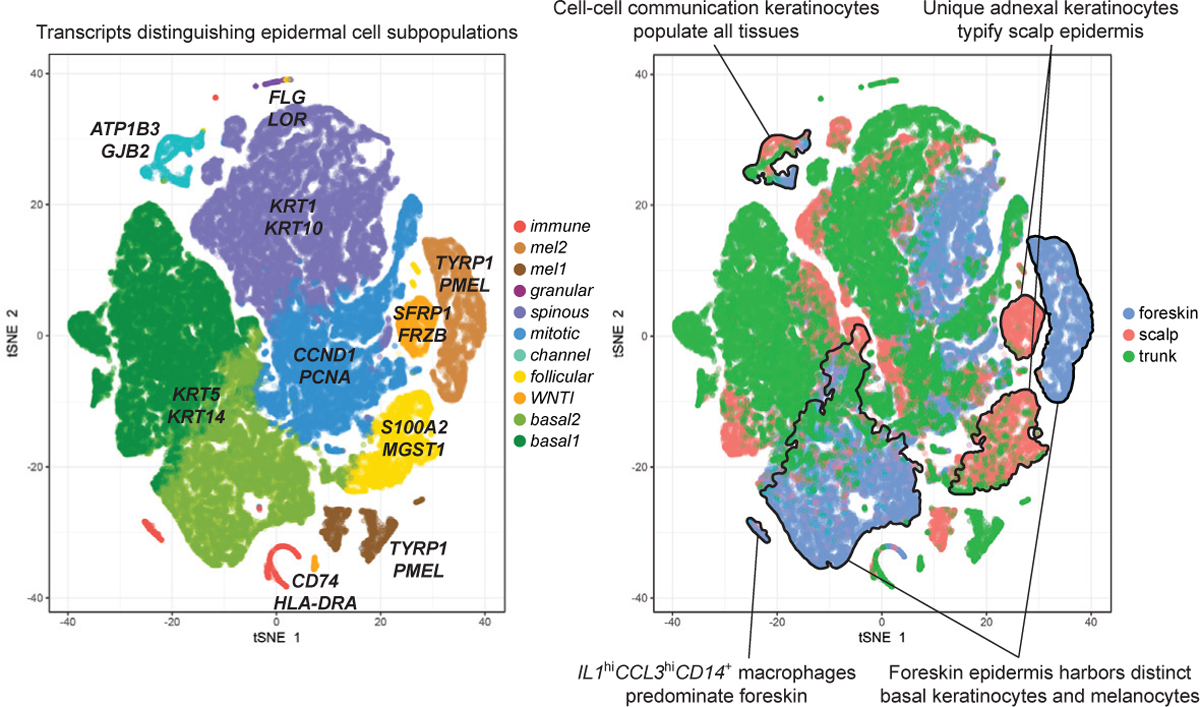

## INTRODUCTION

Epidermal cells of the skin functionally specialize by altering transcriptional identity. Keratinocytes differentiate from a single lineage to form proliferative basal cells, terminally differentiating cells, a cornified barrier layer, and complex appendages such as hair follicles and sebaceous glands (Goldsmith et al., 2012). Keratinocytes also vary dramatically between anatomic sites, generating protective, hyperkeratotic surfaces on volar surfaces but also thin, permeable mucosae. This remarkable functional diversity reflects a heterogeneous and plastic cell identity dependent on transcription of thousands of genes. However, historically, keratinocytes have been classified based only on morphology and selected molecular markers.

Few single-cell expression studies have previously examined the mammalian epidermis. Published data suggest that the transcript abundances of many genes fluctuate during differentiation and murine hair follicle stem cells and transit-amplifying cells possess distinct transcriptional identities (Joost et al., 2016; Yang et al., 2017). The most comprehensive single-cell transcriptional study of the epidermis utilized microfluidics (i.e., the Fluidigm C1 system) to generate a 1,422-cell composite epidermal sample derived from 19 mice (Joost et al., 2016). Although informative, aggregating independent samplings introduces epigenetic and biological variation, reducing the power to distinguish related groups of cells.

We hypothesized that more completely parsing the molecular heterogeneity of a tissue could advance translational science in two important ways. First, it might identify cell populations enriched in specific diseases, making it possible to assess their pathogenic role and nominate them as therapeutic targets. Second, cataloging disease-related variance between individuals or anatomic sites should help classify pathologic states and personalize treatment.

To finely stratify single-cell subpopulations (e.g., hair follicle or immune cells), large numbers of samples must be studied at each anatomic site. We sought here to initially define normal epidermal molecular heterogeneity, laying the foundation required to place future, higher-resolution studies into context. We present an analysis of single-cell RNA sequencing (scRNA-seq) of three human epidermal samples each from adult scalp, adult truncal skin, and neonatal foreskin (a total of 9 samples). We also compared this dataset with that obtained from three additional samples of psoriatic truncal epidermis. We found that the expression of ~12% of assayed transcripts is regulated in stereotyped patterns during epidermal differentiation, most without a known mechanism. Discrete subpopulations of keratinocytes compartmentalize crucial molecular activities, revealing new functional lineages in the human epidermis.

## RESULTS

To interrogate single-cell gene expression at each of these three anatomic sites, normal surgical tissue discards from circumcisions, reduction abdominoplasties and mammoplasties, and scalp excisions were obtained. Three samples of truncal psoriatic skin, obtained from patients not receiving topical or systemic treatment, received a confirmatory histopathologic evaluation from a board-certified dermatopathologist. After sample collection, the epidermis was enzymatically separated from the dermis and dissociated to single cells. Dissociated cell suspensions underwent fluorescence-activated cell sorting to exclude dead cells and cellular debris. Chromium Single Cell 3^ʹ^ v2 libraries were then generated, followed by next-generation sequencing (Illumina; Experimental Procedures).

Sequencing data were analyzed by treating reads from a single droplet as arising from a single cell, using an identifying bar code (Zheng et al., 2017). All reads were thus organized on a per-transcript and per-cell basis. Factors that can reduce the quality of this approach include cell lysis, which raises non-specific background noise, and incorporation of multiple cells per droplet, resulting in profiling of cell doublets. We first performed fluorescence-activated cell sorting (FACS) to exclude dead cells. To minimize doublets, we loaded limiting numbers of cells for each reaction. For this study, we profiled approximately 2,000–12,000 cells per sample with a range of approximately 40,000–115,000 reads per cell (Table S1). On average, 2,334 genes per cell were detected, similar to previous Chromium single cell RNA-seq studies (Skelly et al., 2018; Tsang et al., 2017). A freely available, browsable collection of gene expressions for these datasets is available at http://scarab-research.nantomics.com/.

### Spectral Clustering of Epidermal Single Cells Robustly Identifies Distinct Cell States

By enzymatically dissociating intact epidermis, we expected to generate a heterogeneous mixture of keratinocytes, melanocytes, Langerhans cells, and hematopoietic cells. We discriminated these cell populations by applying spectral clustering (Yan et al., 2009) to the normalized, imputed transcription data (Experimental Procedures; Figure S1). Single cells from multiple tissue samples can be classified in different ways. For example, all cell profiles from multiple specimens can be segregated in a single analysis, favoring the classification of similar cell types shared across samples (Joost et al., 2016). Alternatively, each specimen can be analyzed independently, enabling high-resolution discovery of cell subpopulations specific to that sample.

**Figure 1.**
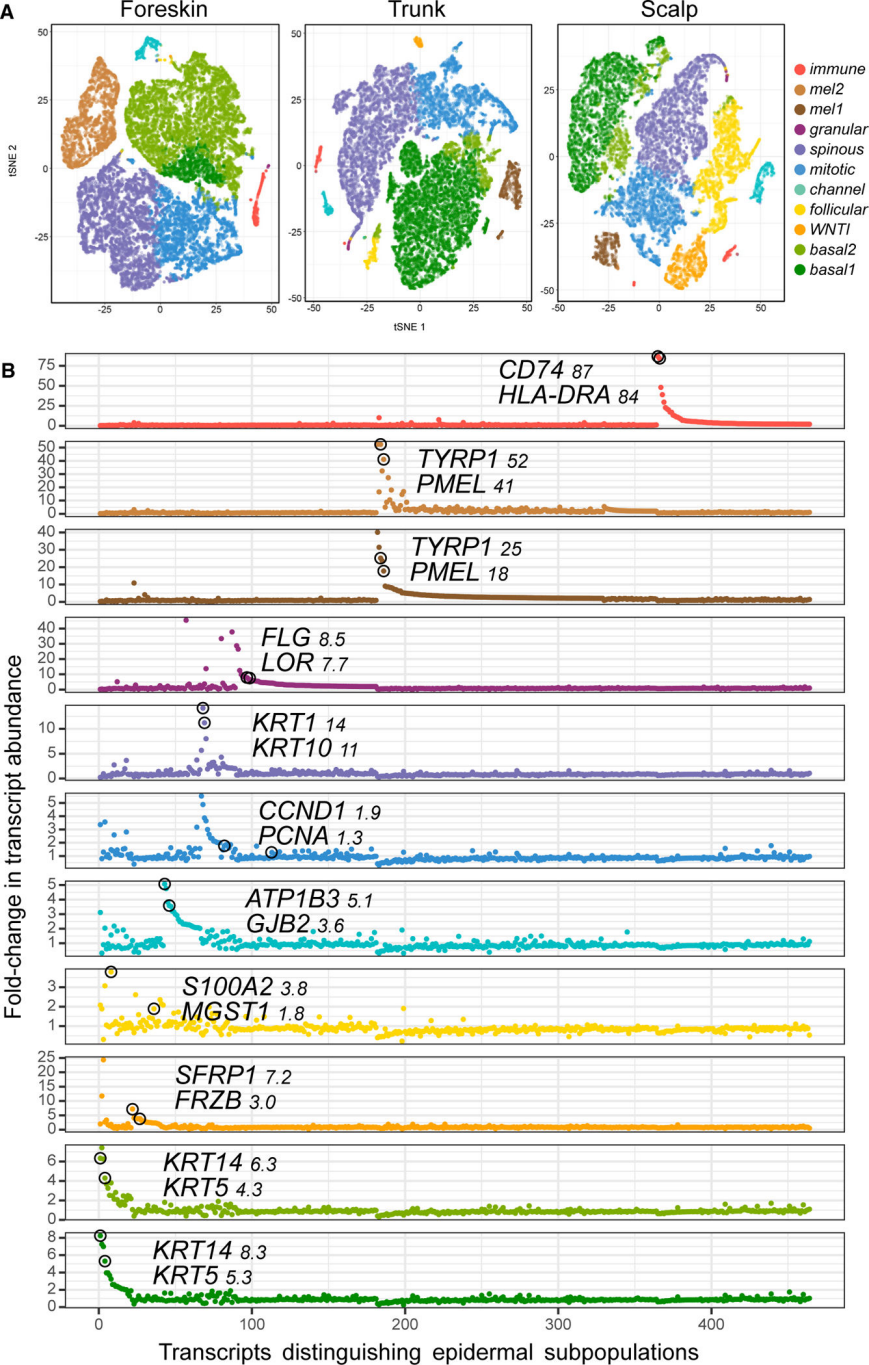
Unbiased Classification of Epidermal Cell Subpopulations in Human Skin (A) t-SNE maps show the relatedness of epidermal cell groups distinguished by spectral clustering from three aggregated samples each from foreskin (26,174 cells), trunk (25,129 cells), and scalp (20,561 cells) (see also Figures S1, S4, and S5 and Data S1). Cells in eight of the 11 clusters express high levels of keratins, identifying them as keratinocytes. Two groups representing melanocytes are coded in brown, and the initial immune cell cluster is depicted in red. (B) Genes with log fold change > 1 in at least 1 cluster ordered by cluster (x axis) and fold change (y axis) (Tables S1, S2, S3, and S6). Genes only appear once, in the first cluster (from bottom to top), where their log fold change passes the threshold. Two genes with specificity for each cluster are named, and their respective coordinates are highlighted with black circles.

We employed the former approach to detect recurrent patterns across our independent samples. To avoid conflating related but distinct groups, we partitioned epidermal cells into different numbers of clusters, with n ranging from 8 to 12. For the remainder of our analyses, we utilized 11 clusters, where many established, important cell populations are clearly distinguished (t-distributed stochastic neighbor embedding [t-SNE] representations of these data are shown in Figure 1A). Pseudo-coloration of these plots by individual sample shows similar representation in each cluster per individual sample (Data S1A–S1C). A single t-SNE plot incorporating all 9 normal samples reproduces the 11 main clusters (Data S1F). For each cluster, genes with high relative expression distinguishing that subpopulation of cells (compared with all other clusters) are depicted in Figure 1B, projected onto t-SNE plots (Data S1G), and cataloged in Table S2. We also tested *k*-means clustering to partition cell profiles. The resulting subpopulations showed close similarity to those from spectral clustering, as judged by differential expression analysis (Table S3), reflecting the robustness of our approach.

### Keratinocyte Subpopulations Demarcate WNT Inhibition and Cell-Cell Communication

We named the two cell populations expressing the highest levels of *KRT5* and *KRT14* (Table S2) ‘*basal1*’ and ‘*basal2*,’ respectively, in accordance with their predicted position in the epidermis. The highest *KRT1*- and *KRT10*-expressing cells also displayed high *DSG1* and *DSP* levels and were termed ‘*spinous*.’ A cluster of cells (‘*granular*’) expressing a suite of late differentiation markers, including *LOR*, *FLG*, and *SPINK5*, was also identified, although at consistently low cell numbers. Because keratinocyte differentiation is a terminal process that culminates with nuclear and organelle loss and cell death (Eckhart et al., 2013), we deduced that more differentiated keratinocytes died during preparation or were excluded by DAPI-negative gating during FACS.

The four remaining keratinocyte clusters all showed intermediate levels of *KRT5* and *KRT14*. One showed coordinate elevation of more than a dozen well-recognized DNA synthesis and cell division transcripts, such as *PCNA* and *KI67*. We therefore termed this cluster ‘*mitotic*.’ Another showed high levels of transcripts whose gene products are secreted and antagonize WNT signaling, including *SFRP1*, *FRZB*, *DKK3* (Cruciat and Niehrs, 2013), and *WIF1* (Malinauskas et al., 2011). The third was elevated for transcripts known to be expressed in human follicular root sheaths (*S100A2*; Mitoma et al., 2014), sebaceous gland and root sheath hair follicles (mouse, *APOE*; Grehan et al., 2001; human, *KRT17*; Troyanovsky et al., 1989), and mouse sebaceous glands (*MGST1*; Joost et al., 2016; *APOC1*; Jong et al., 1998). Finally, a fourth cluster was distinguished by coordinate upregulation of ion channel and cell-cell communication transcripts, including *GJB2*, *GJB6*, *ATP1B3*, *ATP1A1*, *ATP1B1*, *ATP5B*, and *FXYD3*, and also mitochondrial channel proteins such as *VDAC2* and *SLC25A5*. These latter three cell populations were named, respectively, ‘*WNTI*,’ ‘*follicular*,’ and ‘*channel*.’

Two clusters (‘*mel1*’ and ‘*mel2*’) showed markedly higher expression of the *PMEL*, *TYRP1*, and *MLANA* components of the melanocyte pigment synthesis pathway (Hoashi et al., 2005), identifying them as melanocytes. The final cluster was characterized by the high *HLA* levels associated with immune cells.

**Figure 2.**
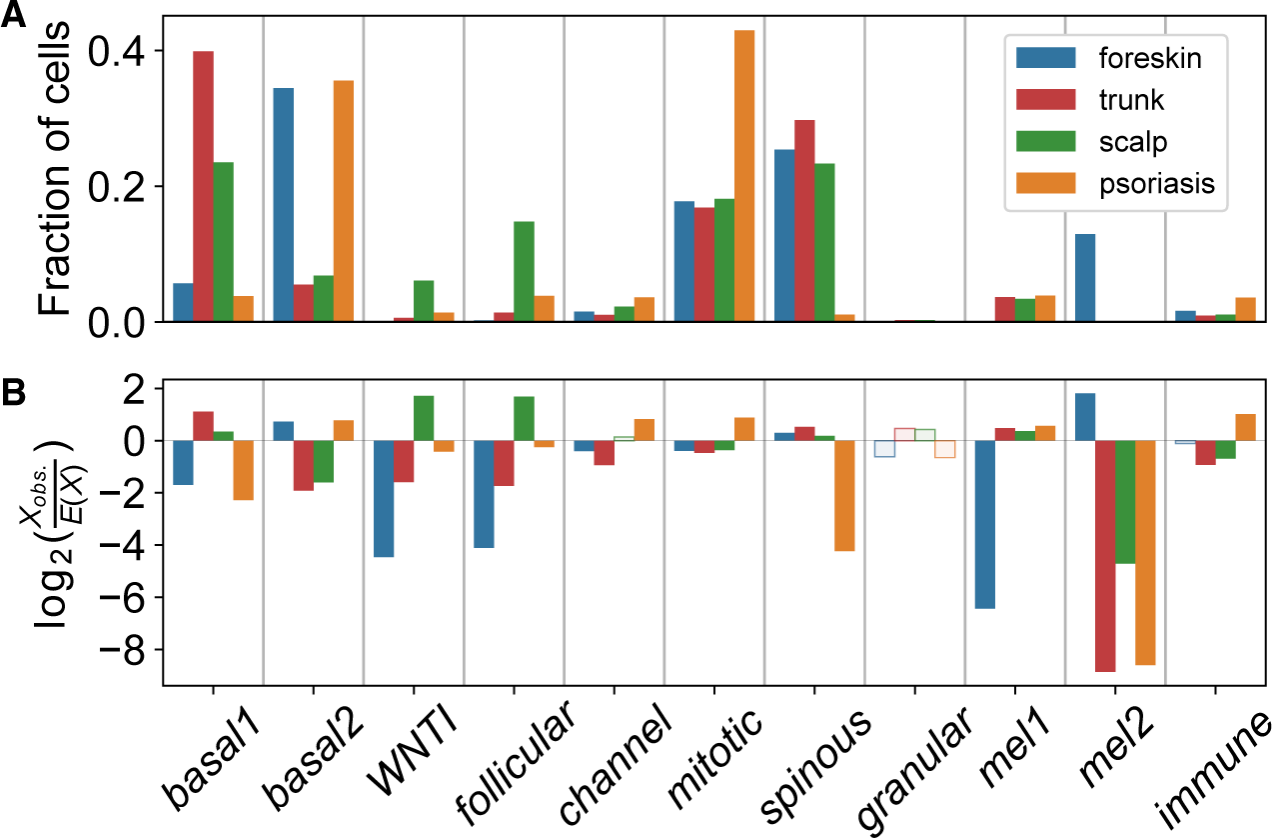
Enrichment of WNTI and Follicular Clusters in Scalp Epidermis (A) Fraction of cells from each anatomic site or psoriatic skin belonging to each cluster. (B) Log ratio of the observed number of cells from an anatomic site or psoriatic skin in the cluster to the expected number when sampling cells in cluster uniformly without replacement. Positive and negative log ratios indicate cluster enrichment and depletion for anatomic site or psoriatic skin. All tissue and cluster associations with solid fill bars are significant (p_adj_ < 0.05, Pearson’s chi-square test with Bonferroni adjustment).

We next asked whether our newly identified keratinocyte subpopulations reflect the gross phenotypic variation in epidermis from different anatomic sites. Large disparities in anatomic distribution were immediately apparent (Figures 1 and 2). The *WNTI* and *follicular* subpopulations were significantly enriched in scalp tissue (p_adj_ < 10^—309^, Pearson’s chi-square test with Bonferroni correction), more sparse in trunk tissue, and almost absent in foreskin tissue, suggesting that they represent components of hair follicles. In other cases, subpopulations appeared to represent distinct versions of a single cell type in different tissues. For example, the *basal1* and *mel1* subpopulations appear to represent the main basal keratinocytes and melanocytes in scalp and trunk cells. In contrast, *basal2* and *mel2* cells predominate in foreskin.

### Temporal Tracing Reveals the Keratinocyte Differentiation Program at Single-Cell Resolution

**Figure 3.**
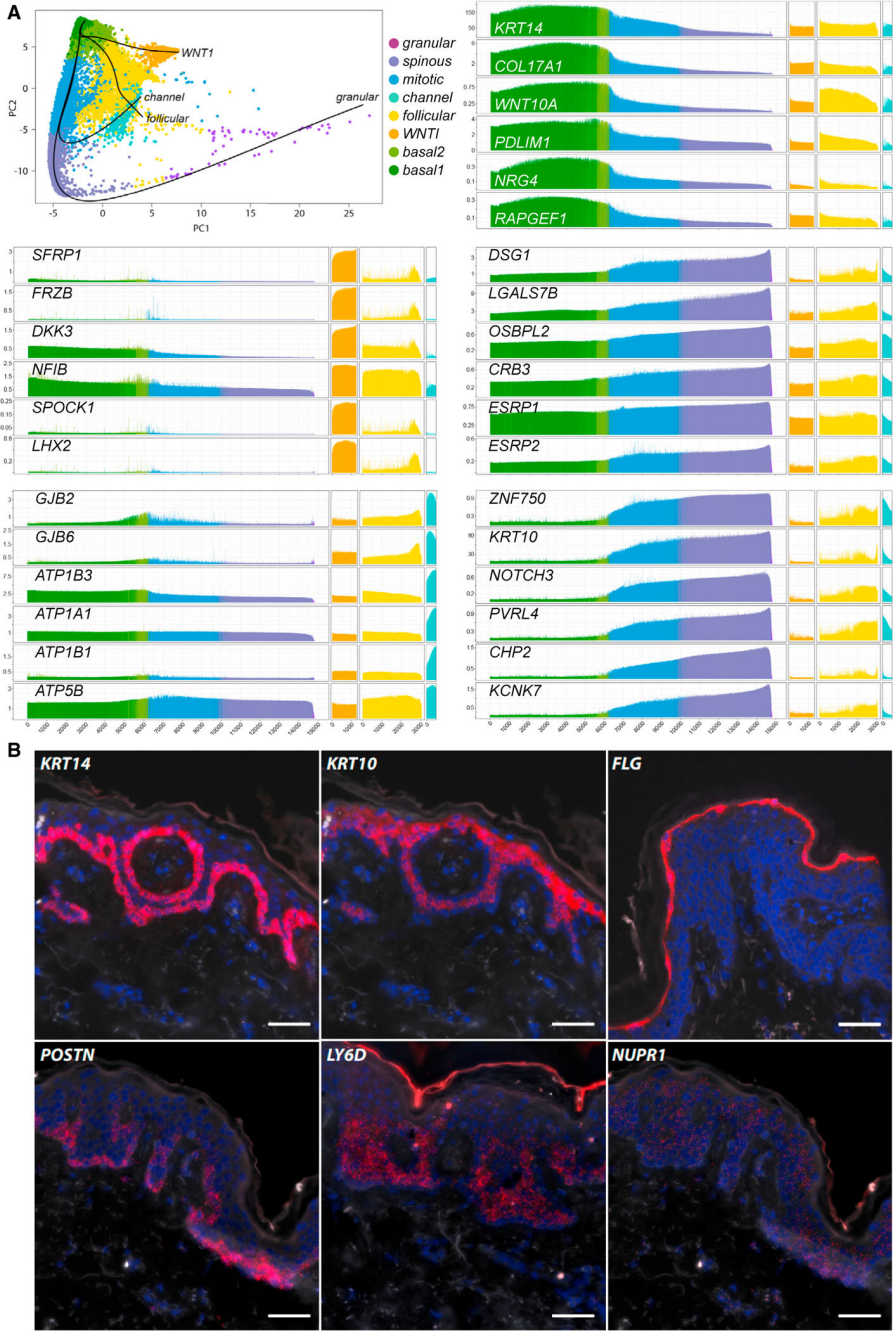
Coordinate, Finely Distinguished Kinetics of Gene Expression in Differentiating Scalp Keratinocytes (A) Top left: the longest pseudotime reconstruction of differentiation (line ending in purple granular cells) defines basic keratinocyte differentiation used in the other panels. Other pseudotime lines show distinct differentiation pathways from basal cells to WNTI, follicular, and channel cells. In the remaining five panels, the leftmost section shows transcript abundance (in imputed counts/10,000, y axis) in about 21,000 pseudotime-ordered differentiating scalp keratinocytes on the x axis, from left to right. Also charted are transcript levels in WNTI, follicular, and channel cells in the remaining 3 sections. Left center and left bottom: genes distinguishing the WNTI and channel clusters, respectively. Right: distinct kinetics of differentiation-dependent transcript regulation. (B) RNA *in situ* hybridization staining (red channel) confirms the layer specificity of genes identified in this report: basal layer *POSTN*, spinous layer *LY6D*, and spinous and granular layer *NUPR1*. The blue channel represents DAPI staining. Scale bars, 50 μm. See also Figure S2.

Keratinocytes undergo a scripted transcriptional program as they travel from a basal, proliferative layer to terminal corneocytes, with ~12% of transcripts differentially expressed between keratinocyte subpopulations (Table S1). We evaluated our eight keratinocyte clusters from normal skin in the context of this progression. We first placed each scalp keratinocyte on a linear spectrum of differentiation based on the expression patterns of established markers: *KRT5*, *KRT14*, *KRT1*, *KRT10*, *IVL*, and *FLG* (Supplemental Experimental Procedures, Pseudotime). As expected, this trajectory partially recapitulated the spectral clustering of keratinocytes, easily visualized by color-coding cells (Figure 3A).

We next used this linear order of cells to inspect both novel and previously undescribed groups of transcripts showing differentiation-related expression. *KRT14* and *COL17A1* show basal-specific expression, reflective of their function at the basement membrane. However, we also discovered a broad array of genes that show closely related patterns of expression; for example, *WNT10A*, *PDLIM1*, *NRG4*, and *RAPGEF1* (Figure 3A). This sort of gene discovery was readily reproduced for other stereotyped expression patterns. The superficial desmoglein *DSG1* predictably shows maximal expression in the granular cluster. However, similar kinetics were seen not only for other cell membrane components (e.g., galectin *LGALS7B*), but also cytoskeletal and cell polarity regulators such as *OSBPL2* (Kentala et al., 2018), *CRB3* (Tapia et al., 2017), and *ESRP1* and *ESRP2* (Warzecha et al., 2010). Notably, genes helping to distinguish the *mitotic*, *follicular*, and *channel* cell clusters did not show linear covariance, indicating that a classic differentiation model of the epidermis fails to distinguish some subpopulations. These data thus highlight the importance of single-cell analysis in discerning cell identities within a heterogeneous population.

We sought to understand the positional specificity of expression patterns in our data. We performed RNA *in situ* hybridization (Kwon et al., 2017) of cluster-specific transcripts alongside genes known to vary with differentiation (*KRT14*, *KRT10*, and *FLG*), as shown in Figure 3B. These data biologically validate our assignments of basal layer expression for *POSTN*, spinous layer expression for *LY6D*, and spinous and granular layer for *NUPR1*. For validation of marker genes for clusters that did not show linear covariance in pseudotime, we performed *in situ* hybridization for *ATP1A1* (which showed a punctate basal and suprabasal pattern that may be representative of the channel cluster) and *KI67 (*which displayed a basal and suprabasal pattern characteristic of the *mitotic* cluster; Figure S2). Additionally, we plotted transcript expression of the *mitotic* cluster cell cycle genes (*PCNA*, *CENPF*, *KI67*, and *CCNA2*) against *KRT10* abundance to show that their expression peaks at an intermediate *KRT10* level (Figure S3).

### Amphiregulin Enrichment Distinguishes a Subpopulation of Foreskin Basal Keratinocytes

Coarse clustering of cells from heterogenous tissues, as in our initial approach, may lack the discriminative power to identify finer subpopulations of cell types. To search for such classes, we re-analyzed the largest cluster in our original analysis (basal keratinocytes comprising *basal1* and *basal2*) in all 9 normal samples, performing spectral clustering at n = 3–10 subdivisions. To avoid finer subdivisions arising from batch artifacts (Figures S4A–S4C), we required that at least 5% of cells in each new cluster derive from each of the three samples of a contributing tissue type.

We focused on n = 3, where all samples from a contributing tissue were robustly represented in each cluster (Figures S4D–S4F). This analysis reproduces the trunk and scalp (*basal1*) and foreskin and psoriasis (*basal2*) dominant subpopulations from the original analysis. In addition, we also identify a new, clearly demarcated cluster specific to foreskin (‘*basal3*’). Limma-based differential gene expression analysis (Ritchie et al., 2015) between these three clusters shows that *basal1* is particularly enriched for *CXCL14* and *DMKN*, whereas *basal2* is enriched for *CCL2* and *IL1R2*, suggesting immunosecretory distinctions among basal keratinocytes. In contrast, *basal3* is highly enriched for amphiregulin (*AREG*), an epidermal growth factor receptor (EGFR) ligand that promotes keratinocyte proliferation (Stoll et al., 2016; Table S2).

### Scalp Keratinocyte Transcriptomes Identify *MGST1* and *TKT1* as Human Follicle Markers and Localize *S100* Overexpression to Interfollicular Epidermis

**Figure 4.**
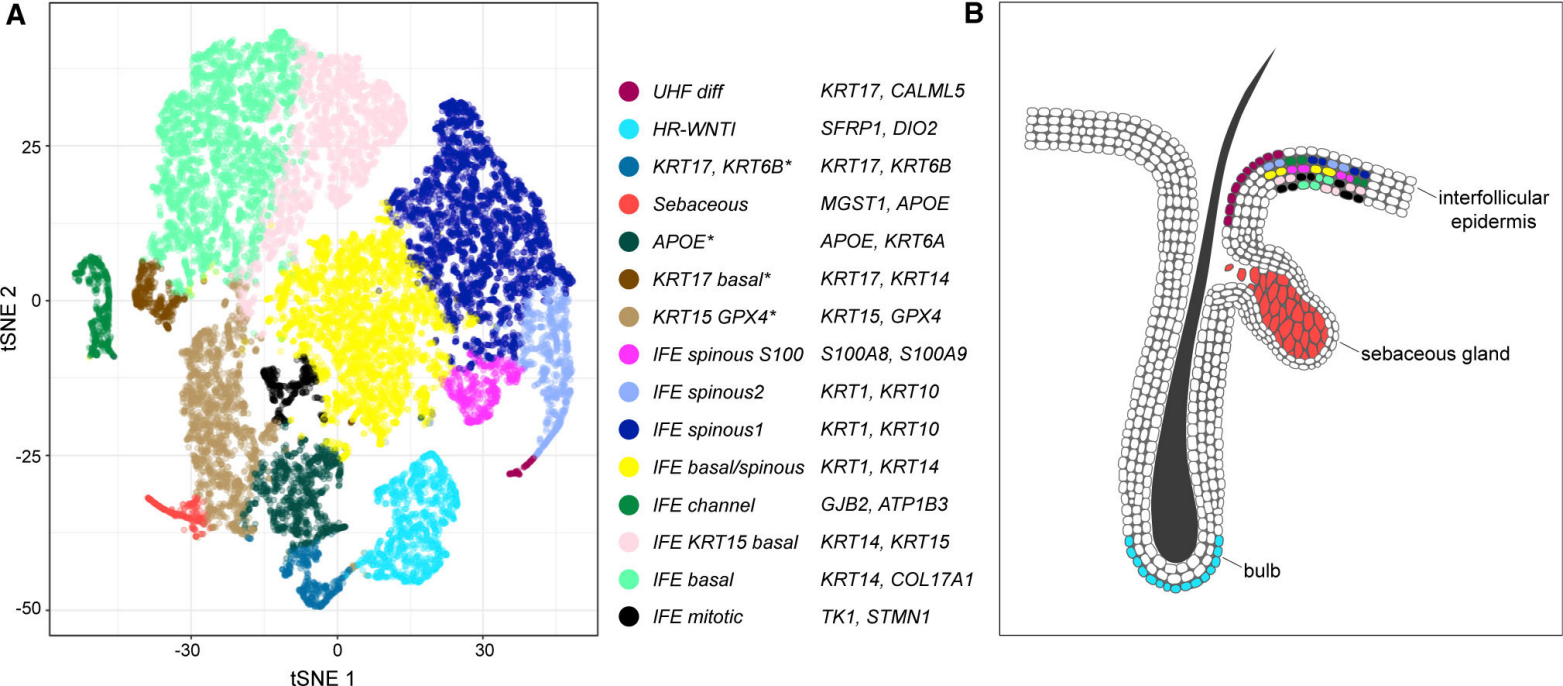
Distinctive Epidermal Cell Subpopulations in Human Scalp Corresponding to Follicular and Interfollicular Keratinocytes The t-SNE map shows spectral clustering of scalp keratinocytes into 15 groups (Table S4), which reveal correlates of outer and inner bulge cells, sebaceous gland cells, upper follicular epithelium, and also recapitulation of multi-site interfollicular epithelium strata. *These cell clusters could not be confidently assigned locations and are not depicted in the follicle diagram. See also Figure S2 and Data S1.

Our successful secondary partitioning of basal epidermis suggested that we also focus this approach on scalp keratinocytes to identify the specialized cell subpopulations characteristic of hair follicles. We re-clustered the keratinocyte populations in our three scalp samples at n = 10–20. At 15 clusters, putative subpopulations in human hair follicles resolved further without duplicating subcategories (Figure 4; Table S4). WNT-inhibitory transcripts found in the initial multi-anatomic site *WNTI* cluster (*SFRP1*, *FRZB*, and *DKK3*) again localized to a discrete cellular population in this scalp-specific analysis (named *high-resolution WNTI*, or ‘*HR-WNTI*’). These cells may represent outer bulge cells, which, in mice, have been shown to secrete WNT inhibitors, influencing differentiation of the inner bulge (Lim et al., 2016). The elevated *MGST1* and *APOE* transcripts of the *follicular* cluster were also identified in a scalp subpopulation we named ‘*sebaceous*’. Our higher-resolution clustering additionally identified ‘*UHF diff*,’ a cluster potentially analogous to mouse differentiated upper hair follicles (*CST6* [Veniaminova et al., 2013]; *KRT17* [Joost et al., 2016]; *KRT79* [Joost et al., 2016]).

To validate the spatial specificity of markers from these clusters, we used *in situ* staining to localize RNA for *MGST1*, *TKT*, and *SFRP1* (Figure S2). *MGST1* (previously reported as a mouse sebaceous gland marker; Joost et al., 2016) and *TKT* are prominently expressed in the sebaceous gland epithelium, with *TKT* expression most pronounced at the periphery. *SFRP1* shows strong expression in the cuboidal cells of the outer root sheath at the base of the hair follicle.

Distinct interfollicular epidermis (IFE) clusters identified in the multisite analysis also appeared in our scalp analysis, including analogs of the *basal*, *spinous*, *mitotic*, and *channel* subpopulations. Notably, elevated *S100A7*, *S1008*, and *S100A9* expression was restricted to a distinct IFE cluster, a class not resolved in the lower-resolution multi-site analysis.

### Epidermal Subpopulations Shift Immunological and Proliferative Programs between Anatomic Sites

**Figure 5.**
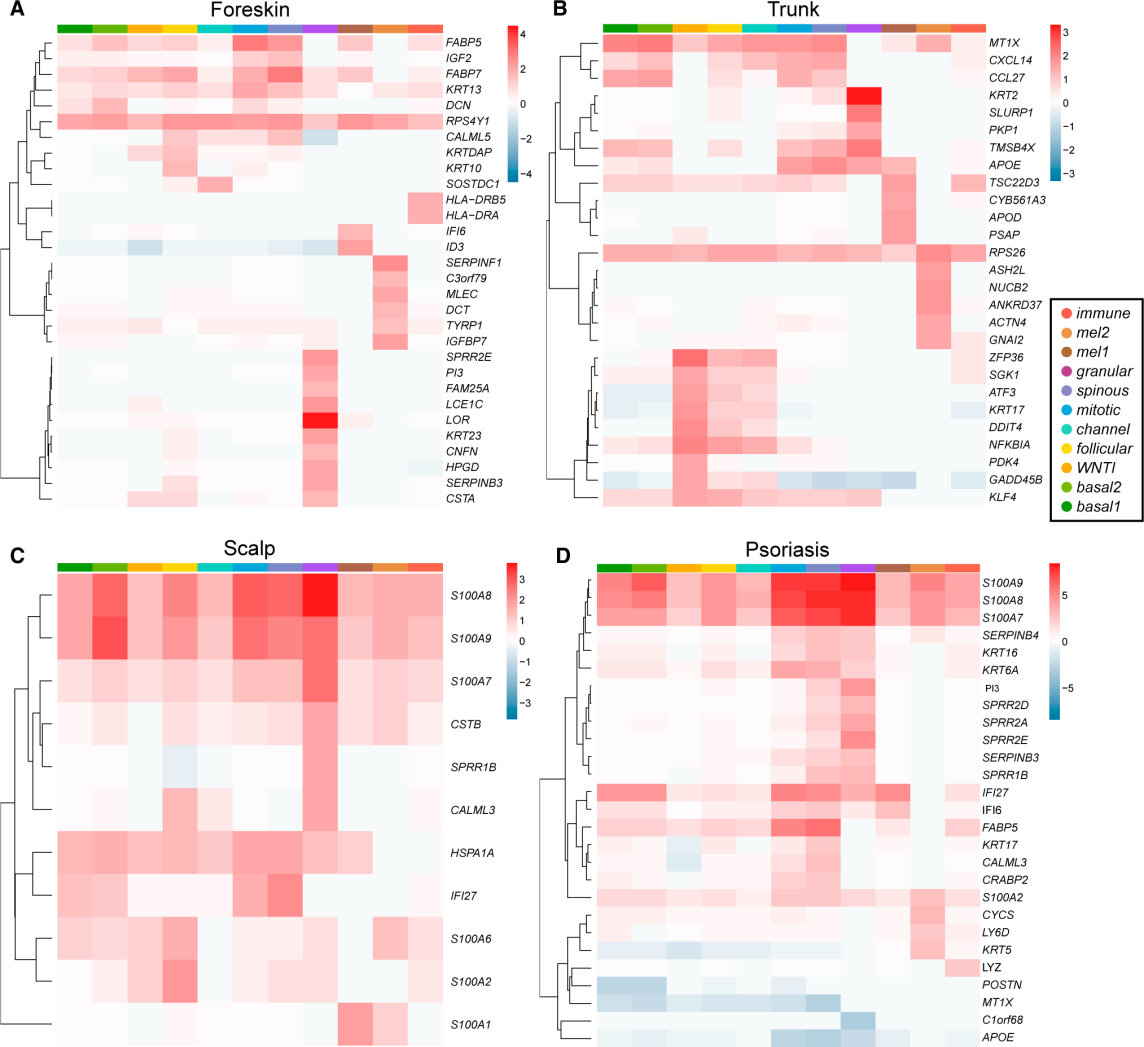
Inflammatory S100 and IFI27 Elevation in Human Scalp and Psoriatic Skin (A–D) Heatmaps showing fold change of differential gene expression, by cluster, for (A) foreskin, (B) trunk, and (C) scalp, each relative to the other anatomic sites. In (D), psoriatic epidermal subpopulations are compared only with normal trunk. In each cluster, limma-trend was used to calculate the fold change and statistical significance of the difference in the mean expression of each gene for each tissue comparison based on non-imputed UMI counts per million (Table S5). Genes with a log_2_ fold change (FC) greater than 1.5 (scalp and foreskin) or 2.5 (psoriasis) and false discovery rate (FDR) < 0.05 in at least one cluster are displayed (Experimental Procedures; Tables S5, S6, and S7). An elevated RPS4Y1 transcript in all foreskin clusters confirms Y chromosome-specific bias for sex.

When a cell subpopulation is found at multiple anatomic sites, transcriptional differences at the different sites may arise within this classification, revealing distinct functional specialization. We therefore directly compared our original 11 aggregate normal tissue epidermal subpopulations between anatomic sites, utilizing differential gene expression analysis (Figure 5; Experimental Procedures; Tables S5 and S6). The most dramatic disparity was detected in scalp, where inflammatory genes were enriched in the set of all transcripts showing scalp-specific upregulation (Fisher’s exact test, p = 2.1 × 10^—3^; Experimental Procedures). Specifically, inflammation-related transcripts of the *S100* family (*S100A7*, *S100A8*, and *S100A9*) as well as *IFI27* were generally elevated compared with other tissues. Sub-clustering of scalp cells identified an IFE subpopulation particularly enriched for these transcripts (Table S4).

Aside from the immunosecretory and proliferative differences in basal keratinocytes discussed above, truncal skin was elevated for *CXCL14*, *CCL27*, and *NFKBIA*, suggesting that the innate immune repertoire of keratinocytes may more generally vary with anatomic site.

We also performed gene ontology (GO) analysis with the Database for Annotation, Visualization and Integrated Discovery (DAVID) (Huang et al., 2009a, 2009b) to assess whether keratinocytes in aggregate from different anatomic sites showed functional enrichment. Foreskin keratinocytes were enriched for cell division, mitotic nuclear division, and RNA splicing and processing terms, consistent with the known greater proliferative capacity of neonatal keratinocytes (Table S7; Gilchrest, 1983).

### Psoriatic Epidermis Is Enriched for *Mitotic* and *Channel* Keratinocytes and *CD1C*^+^ *CD301A*^*+*^ Myeloid Dendritic Cells

We assessed how inflamed epidermis is transcriptionally altered on a single-cell level. Psoriatic keratinocytes were enriched for the *mitotic* and *channel* subpopulations, showing the plasticity of cell transcriptional identities in disease states (Figure 2; p_adj_ < 10^—309^ and p_ad_ = 1.4 × 10^—74^, respectively; Pearson’s chi-square test with Bonferroni correction). Transcripts of *S100* genes were generally elevated in psoriatic epidermis, most markedly in the superficial *spinous* and *granular* cell clusters (Figure 5D). Notably, *S100* transcripts were also elevated in melanocytes and immune cells of psoriatic skin, revealing a multi-lineage response to epidermal inflammation.

**Figure 6.**
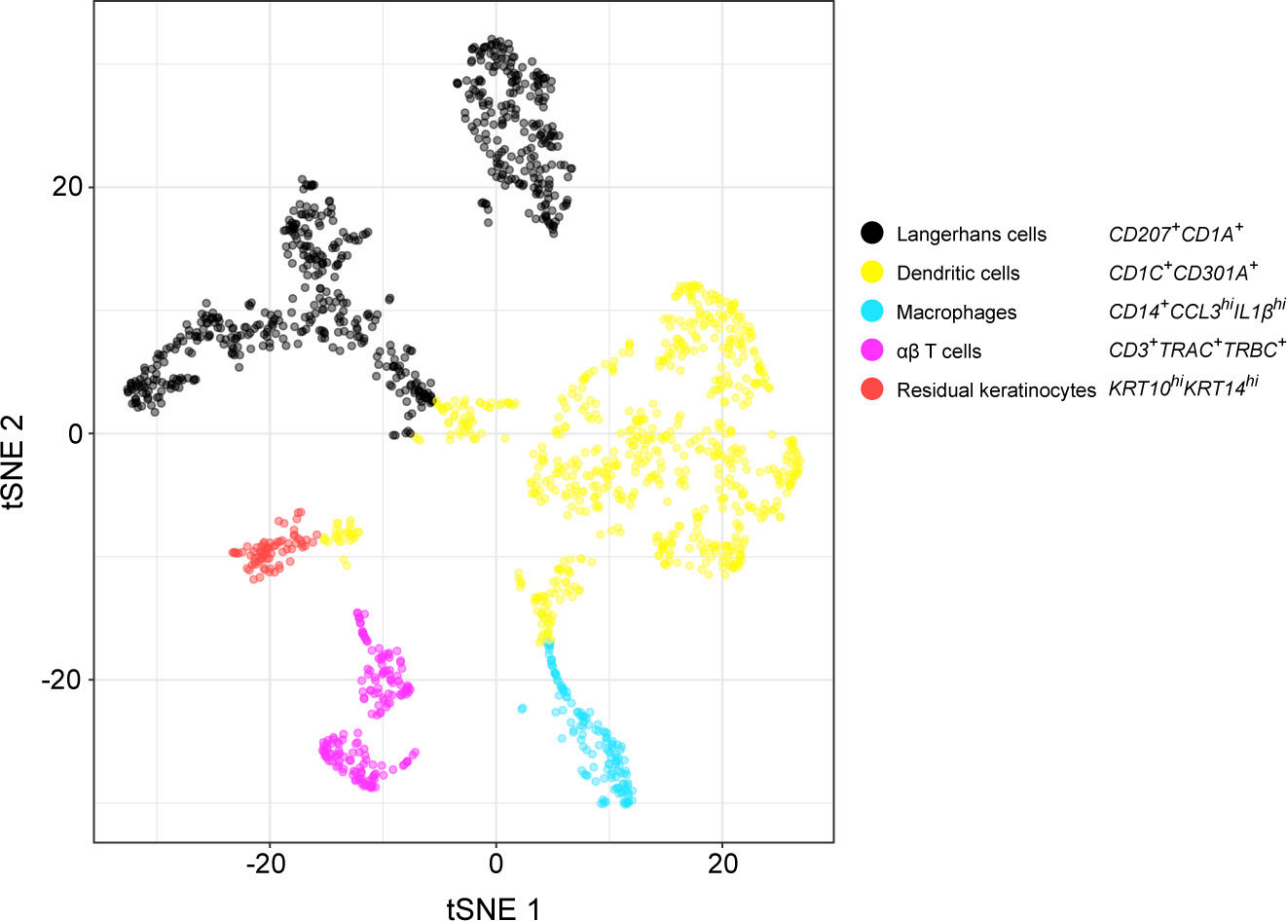
Unbiased Detection of T Cells, Macrophages, and Dendritic Cells in Normal and Inflamed Epidermis The t-SNE map shows spectrally clustered hematopoietic cell populations from all 12 of our normal and inflamed epidermal samples. The spatial adjacency of Langerhans cells, dendritic cells, and macrophages illustrates their relatedness compared with T cells (purple) and a small number of keratinocytes expressing inflammatory transcripts and, thus, misclassified with immune cells (red). See also Table S8 and Data S1.

We further partitioned the immune cell epidermal subpopulation from our normal and psoriatic samples into 5 clusters (Supplemental Experimental Procedures) and identified CD3^+^ αβT cells as well as three clusters representing major histocompatibility complex (MHC) class II^+^ antigen-presenting cells of the myeloid lineage: *CD207*^+^*CD1A*^+^ Langerhans cells, *CD1C*^+^*CD301A*^+^ myeloid dendritic cells (DCs), and *CD14*^+^*CCL3*^hi^*IL1β*^hi^ monocyte-derived macrophages (Figure 6). We did not identify distinct hematopoietic clusters representing other lymphoid or myeloid subsets— e.g., γδ T cells or neutrophils.

**Figure 7.**
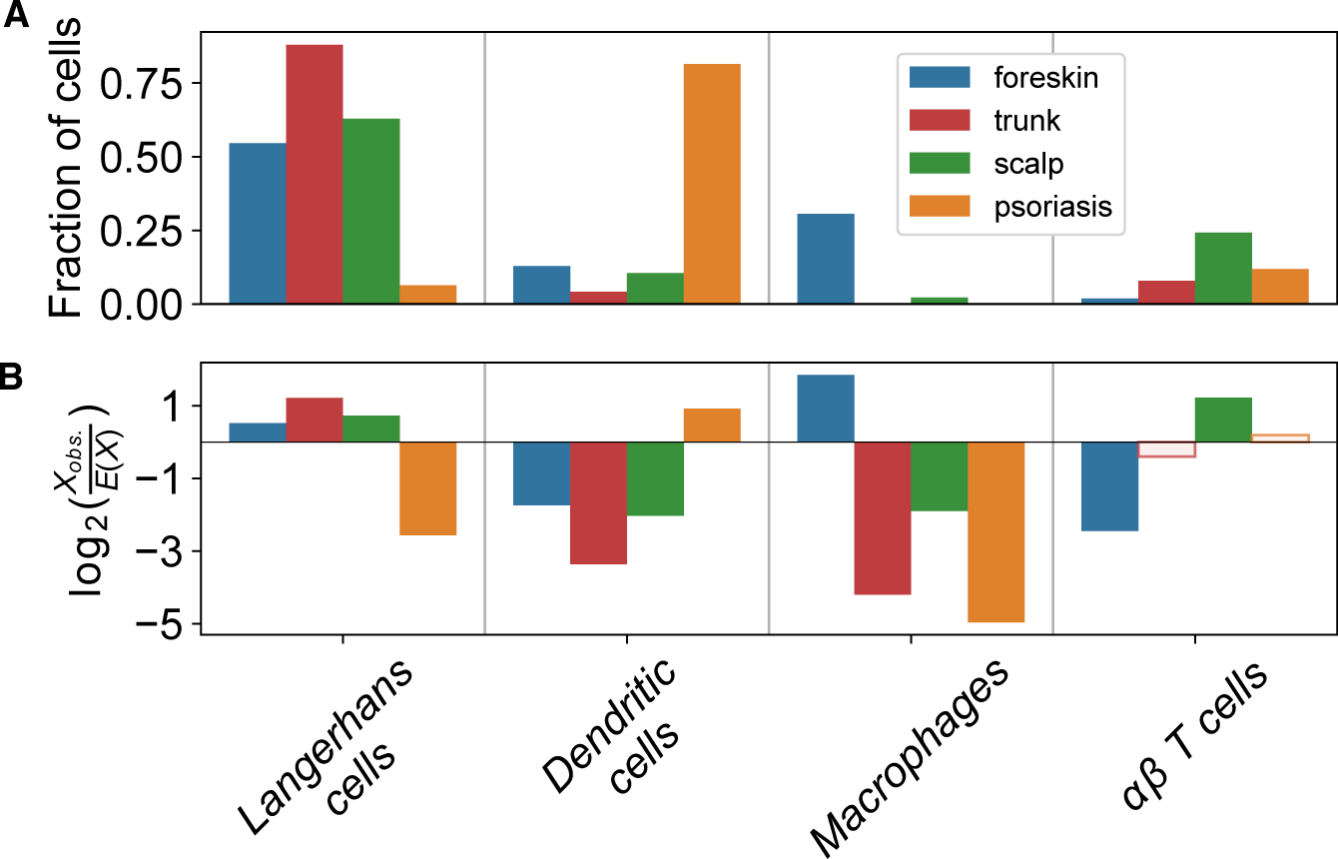
Enriched Antigen-Presenting Cells in Psoriatic Epidermis and Macrophages in Foreskin (A) A fraction of cells from each anatomic site or psoriatic skin belonging to each immune cluster. (B) Log ratio of the observed number of cells from anatomic site or psoriatic skin in cluster to the expected number when sampling cells in the cluster uniformly without replacement. Positive and negative log ratios indicate cluster enrichment and depletion for anatomic site or psoriatic skin. All tissue and cluster associations with solid fill bars are significant (p_adj_ < 0.05, Pearson’s chi-square test with Bonferroni adjustment). No trunk cells occurred in the macrophage cluster, so a pseudo-count of 1 cell was added to allow illustration of the log_2_ fold depletion.

Mapping our 12 samples onto these 5 immune cell clusters revealed biases related to anatomic site and inflammatory state. *CD1C*^+^ *CD301A*^*+*^ myeloid DCs were enriched in psoriatic epidermis, present in scalp skin and foreskin, and sparse in truncal epidermis (Table S8; Figure 7). In contrast, *CD14*^+^ *CCL3*^hi^*IL1β*^hi^ macrophages were generally restricted to foreskin. *CD207*^+^*CD1A*^+^ Langerhans cells were detected across all anatomic sites but underrepresented in psoriatic skin.

There was a trend toward increased CD3^+^ αβ T cells in psoriatic trunk and scalp samples. Although the T cell lineage markers *CD4*, *CD8*, and *FOXP3* were not detectable within the CD3^+^ αβ T cell cluster, specific genes suggested a heterogeneous population of activated, antigen-experienced T cells (*IL2RG*, *CD69*, and *CD44*), cytotoxic T cells (*GZMA* and *GZMB*), and regulatory T cells (*IL2RG* and *TNFRSF18*). Notably, the only CD4 T helper cell lineage-defining transcription factor identified was *GATA3* (low positive). We did not detect *TBET* or *RORC*.

## DISCUSSION

The human epidermis epitomizes the complexity of a multi-lineage tissue. Not only does its function rely on exquisite, stratified programming of keratinocytes spatially co-organized with immune and nerve cells, but the tissue is also epigenetically repurposed in distinct skin locations. Furthermore, the epidermis evolves multi-dimensionally to counter challenges such as infection or wounding. Here we report the first single-cell transcriptional profiling of human epidermis from multiple anatomic sites based on matched transcriptomes of keratinocytes, melanocytes, and immune cells. The data underlying this study, which also includes three samples of psoriatic epidermis, represent approximately 8.4×10^8^ unique molecular identifiers of transcript abundance in 92,889 cells, a vast compendium available for analysis in diverse biological contexts, most beyond the scope of this paper. However, these foundational analyses already present fascinating insights into epidermal composition and function.

The algorithmic partitioning of transcriptomes from keratinocytes, the most abundant cell in the epidermis, partially recapitulates known differentiation strata such as the basal epidermis, *stratum spinosum*, and *stratum granulosum*. We were able to further subclassify populations to identify subpopulations such as *basal3* in foreskin, whose enrichment for the EGFR ligand *AREG* may help explain the higher proliferative potential of neonatal skin (Gilchrest, 1983). As in the Joost et al. (2016) mouse epidermal scRNA study, a specific progenitor population was not clearly resolved by current partitioning approaches.

However, our analysis also demonstrates the presence of previously undescribed subcategories that are not a linear correlate of stratified differentiation. The *channel*, *follicular*, and *WNTI* classes are identified independently both by spectral and k-means clustering of the transcriptomics data (Tables S2 and S3), suggesting a discrete, reproducible nature. In a previous study (Joost et al., 2016), 25 distinct mouse epidermal subpopulations were reported, including 5 subpopulations from the interfollicular epidermis (Joost et al., 2016). Despite a degree of arbitrariness in clustering, this number of groups is within range of the 15 identified in our high-resolution analysis of human scalp (Figure 4; Table S4). The mouse study established layer specificity for numerous genes that also show such patterning in the human epidermis, including *POSTN*, *LY6D*, and *NUPR1*.

The enrichment of *WNTI* and *follicular* cells in scalp, low levels in trunk, and virtual absence from foreskin suggest that they represent elements of terminal hairs. We examined both gene expression and *in situ* staining to map our finer (15 class) division of scalp keratinocytes to follicular structures. The *HR-WNTI* cluster retains elevated levels of *SFRP1*, *DKK3*, *KRT15*, *WIF1*, *PHLDA1*, *DIO2*, *DPYSL2*, *DCN*, and *DCT*, all previously identified as selectively upregulated in the human hair bulge (Lim et al., 2016; Ohyama et al., 2006). *In situ* localization of *SFRP1* to the outer root sheath of the hair bulb suggests that this bulge-related population suppresses Wnt signaling, perhaps in regressing telogen hair follicles (Geyfman et al., 2015).

The scalp-specific sebaceous population contains elevated levels of *MGST1*, a glutathione S-transferase recently observed in mouse sebaceous glands (Joost et al., 2016). *MGST1* diffusely stains the central portion of sebaceous glands harboring mature cells preceding holocrine secretion. However, the *TKT* transcript is sharply delimited to the peripheral germinative layer, suggesting that transketolase correlates with sebocyte proliferation and renewal (Hinde et al., 2013). Further *in situ* staining for differentially expressed transcripts in our scRNA data may identify additional novel, spatial markers in epidermis.

Intriguingly, clinically normal scalp epidermis consistently shows upregulation of inflammatory *S100* transcripts in the IFE. Uninflamed scalp keratinocytes also express greater *IFI27* levels, particularly in the *granular* cluster, another marker typifying psoriatic epidermis (Suomela et al., 2004) and functionally capable of driving keratinocyte proliferation (Hsieh et al., 2015; Figure 5C). Elevation of *S100A9* has been detected in clinically normal scalp of psoriatic individuals (Ruano et al., 2016). However, this is the first report, to our knowledge, that establishes high levels of these transcripts in normal scalp, suggesting a cause for the inflammation, pruritus, and scale often observed at this site. In foreskin keratinocytes, we instead find upregulation of proliferation-related transcripts. Foreskin also expresses a different suite of inflammatory transcripts, perhaps reflecting distinct immunosurveillance at this site. Because of the challenges in obtaining female neonatal or adult genital tissue, it remains to be determined whether these molecular characteristics are associated primarily with genital or neonatal skin.

The striking induction of pore and intercellular communication transcripts in the putative *channel* cluster describes a potentially novel keratinocyte subpopulation. Its relatively consistent abundance in foreskin, trunk, and scalp samples suggests a universal cell identity. Although *GJB2* is found in both follicular and inter-follicular keratinocytes, our *in situ* staining of *ATP1A1*, highly specific and a potential marker for this cluster, displays punctate localization to basal and suprabasal interfollicular epidermis, suggestive of a role as a specialized subpopulation. In the higher-resolution clustering of scalp epidermis, the *channel* subpopulation segregates away from hair follicle markers such as *KRT17*, also supporting its position in interfollicular epidermis. Combinatorial staining of additional *channel* markers should help more precisely localize these cells in normal and diseased epidermis.

Psoriatic epidermis is enriched for *channel* cells (Figure 2B), which show elevated levels of the psoriasis-associated keratins *KRT6A* and *KRT16*. Gene expression of the established psoriasis risk gene *GJB6* (connexin 30.3) is also increased (Sun et al., 2010). Thus, the increased expression of these genes in inflamed skin may be at least partly explained by the expansion of rare *channel* cells in normal skin. Peak expression of *DEFB1* in this cluster, and the known association between *KRT16* and innate immunity (Lessard et al., 2013), support an inherent inflammatory nature of these cells. Germline variants in *GJB6* and *GJB2* (connexin 26), which are similarly enhanced in this subpopulation, cause keratitis-ichthyosis-deafness syndrome, typified by transient inflammatory erythrokeratoderma (Richard et al., 2002). How the expansion of *channel* cells contributes to the behavior of psoriatic or inflamed epidermis remains to be explored.

In addition to enrichment for *channel* cells, psoriatic epidermis is remarkable for expansion of the *mitotic* subfraction, (p_adj_ < 10^—309^, Pearson’s chi-square test with Bonferroni correction), revealing its proliferative nature. Although the dividing fraction of keratinocytes is commonly assigned to the *stratum basale*, charting these cell cycle-related transcripts against *KRT10* expression in our data suggests that they remain active well into suprabasal layers (Figure S3). Our *in situ* staining of *KI67* supports this interpretation (Figure S2). Our single-cell expression data also show enhancement of inflammatory transcripts peaking in the suprabasal layers of psoriatic skin. Some have been identified previously, including *S100A7* (Wolf et al., 2008) and *S100A8* and *S100A9* (Benoit et al., 2006), but some appear novel, as for *IFI27* and *PI3.*

Our immune cell analysis is limited by our focus on the epidermis, excluding the diverse, important subpopulations that reside primarily in the dermis. Furthermore, in this study, we profile immune cells in true proportion to keratinocytes of the epidermis without the CD45 enrichment required for higher-resolution analysis. Therefore, important immune cell populations are represented at very low abundance (for example CD3^+^ αβ T cells; Figure 6), making it challenging to partition them into their constituent CD4 or CD8 components. However, even at this relatively low resolution, we discern previously undescribed patterns of immune cell activation, primarily in the DCs abundant in the epidermis.

We find psoriatic epidermis to be surprisingly enriched for *CD1C*^+^*CD301A*^+^ myeloid DCs relative to normal epidermis from all anatomic sites, with fewer macrophages and Langerhans cells. Although dermal DC populations have been established to expand in psoriatic skin (Zaba et al., 2009), our data suggest that this specific subset of DCs also markedly expands in psoriatic epidermis. Thus, epidermal DC proliferation and/or recruitment may further fuel activation of effector lymphoid and myeloid cells to produce the clinical features of psoriatic skin. We also report that macrophages are highly enriched in foreskin, possibly involved in antigen presentation from neonatal or urogenital pathogens.

Even in our relatively sparse CD3^+^ αβ T cells, we detect elevated transcripts representative of *GZMA*^+^*GZMB*^+^ cytotoxic T cells and *CD44*^+^ memory cells, not previously considered primary residents of normal skin. Whether this population includes the recently described CD8^+^CD49^—^ resident memory T cells enriched in psoriatic skin is unclear (Cheuk et al., 2017). CD3^+^ αβ T cells are also notably elevated in normal scalp skin. Skin-resident T cells are known to cluster around terminal hair follicles, with diverse functions including hair follicle cycling and immune response to microbiota (Ali et al., 2017). T cell enrichment in scalp epidermis may simply reflect high hair density in this site. However, the cytotoxic T cell subpopulations suggested by our data may be relevant for the pathogenesis of lymphocyte-mediated alopecias (Bolduc et al., 2016; Xing et al., 2014). Future studies in which hematopoietic lineage cells are enriched after epidermal dissociation should greatly enhance resolution when examining T cell compartments.

The keratinocyte and immune cell transcriptional programs we report in psoriatic epidermis demonstrate how scRNA-seq rapidly provides a multi-dimensional fingerprint of inflammatory disease. This approach may classify rashes whose origins could not previously be determined and match treatments to rashes in specific anatomic regions.

The expansive dataset published here lends itself to many additional analyses. Protein or mRNA expression of some genes has been associated previously with specific epidermal layers. However, the closely related gene expression patterns in Figure 3A can be exploited to detect new regulatory elements coordinating their activity. For example, suprabasal expression of the keratinocyte differentiation factor *ZNF750* shows kinetics precisely related to epithelial regulators but also poorly characterized genes such as *NOTCH3*, *PVRL4*, *CHP2*, and *KCNK7* (Figure 3A). Sequence-based and functional studies may now elucidate the regulatory mechanisms effecting their parallel control.

## EXPERIMENTAL PROCEDURES

### Tissue Isolation

For each of the three anatomic sites examined, normal surgical tissue discards from circumcisions, reduction abdominoplasties or mammoplasties, or scalp excisions were obtained. Written informed consent for the samples was obtained using protocols approved by the University of California, San Francisco (UCSF) institutional review board. Fresh tissue was initially placed in medium 154 with human keratinocyte growth supplement and 0.07 mM CaCl_2_ (Thermo Scientific) and stored at 4^○^C for 1–2 days prior to single cell isolation. The epidermis was enzymatically dissociated from the dermis with dispase (Corning) incubation for 2 hr at 37^○^C. Epidermal sheets were then manually separated from the dermis and then dissociated into single cells with trypsin (Thermo Scientific) incubation at 37^○^C for 15 min. The dissociated cell suspension was strained with a 40-μM filter, washed with medium 154, and then underwent FACS to exclude DAPI-positive dead cells, doublets, and debris. Sorted cells were centrifuged and resuspended in 0.04% BSA (Sigma) in PBS (Thermo Scientific). Chromium Single Cell 3^ʹ^ v2 (10X Genomics) library preparation was then performed by the Genomics Core Facility, UCSF Institute for Human Genetics, according to the manufacturer’s protocol.

### Sequencing

Chromium Single Cell 3^ʹ^ v2 libraries were sequenced with either an lllumina HiSeq 2500, HiSeq 4000, or NovaSeq 6000 following the manufacturer’s protocol. For libraries sequenced using Illumina HiSeq 2500 (high output mode) or HiSeq 4000, the following sequencing parameters were used: read 1, 26 cycles; i7 index, 8 cycles; i5 index, 0 cycles; and read 2, 98 cycles. For libraries sequenced using a NovaSeq 6000 S2 reagent kit, the following paired-end sequencing parameters were used: read 1, 26 cycles; i7 index, 8 cycles; i5 index, 0 cycles; and read 2, 91 cycles.

### Primary Computational Analysis

Primary computational analysis started from raw Illumina sequencing data and culminated in cell clusters. Raw data were processed using Cellranger (10X Genomics version 2.0.2) and filtered using Seurat (Macosko et al., 2015; Supplemental Experimental Procedures, Data Processing and QC Filtering). We used zero-inflated negative binomial-based wanted variation extraction (ZINB-WaVE) (Risso et al., 2018) to obtain a low dimensional representation of cells, removing variations attributable to library size, mitochondrial read composition, and batch effect. We used the Markov affinity-based graph imputation of cells (MAGIC) imputation algorithm (van Dijk et al., 2017) with cell-cell similarity measured by the ZINB-WaVE low-dimensional representation to mitigate effects of dropout (Supplemental Experimental Procedures, Imputation, Choice of Magic t Parameter). Imputed expression values were used to cluster cells by applying principal component analysis (PCA), followed by k-means-based approximate spectral clustering (Yan et al., 2009; Supplemental Experimental Procedures, Principal Component Analysis, Spectral Clustering). Finally, we used Slingshot (Street et al., 2018) to assign developmental pseudotime to the scalp cells and demonstrated that the clustering results are robust (Supplemental Experimental Procedures, Pseudotime, t-SNE Mapping, Processing Time, and Sex-Specific Bias Analysis).

### Differential Expression

We used limma-trend version 3.34.8 (Law et al., 2014) based on the application of this method to scRNA data from Soneson and Robinson (2018). For this analysis, we considered a set of 6,337 genes that had at least 3 unique molecular identifiers (UMIs) in at least 100 cells across all samples. UMI count data are converted to log-scaled counts per million (log-CPM) with an offset of 1. Linear models are fit to the log-CPM profiles of each transcript with membership of each cluster as a binary covariate. To evaluate differential gene expression between clusters, the mean log-CPM of each gene in the cells in each cluster is compared with the mean log-CPM of the gene in all other cells. To evaluate the differential expression of transcripts in each tissue on a percluster basis, we fit independent linear models for the cells in each cluster with tissue membership as a binary covariate. For each transcript, the percluster mean log-CPM for each healthy tissue was compared with the mean across the other two healthy tissues. The mean log-CPMs in psoriatic cells were compared with the means in truncal cells. Moderated t statistics for the differences in means are calculated using an empirical Bayes approach. False discovery rate is calculated from p values associated with the t statistics to evaluate statistical significance.

### GO Analysis

We performed two-part GO analysis on lists of genes with consistent differential expression between foreskin, scalp, and trunk across keratinocyte subpopulations. Lists were constructed by first restricting attention to the set of genes differentially expressed between tissues in any keratinocyte subpopulation (Table S5; p_adj_ < 0.05); each gene was then assigned to a tissue, along with its direction of differential expression, when the gene was differentially expressed in that tissue in at least 1 subpopulation and not differentially expressed in the same direction for any other tissue across all subpopulations. A single gene, *HACD1*, was assigned as having tissue-specific expression in trunk tissue in both directions and was excluded from downstream analysis. Table S7 provides the resulting gene lists with direction of differential expression.

Using these gene lists, we first investigated whether the apparent enrichment for inflammatory function in scalp-specific genes, depicted in Figure 5, was significant when extended to all genes upregulated in scalp keratinocytes. Significance was assessed with Fisher’s exact test applied to the intersection of this gene list with a set of genes annotated for inflammatory response in Uniprot or as positive regulators of inflammatory response in AmiGO. The background gene set consisted of all genes with raw expression measurements.

Second, we used DAVID (Huang et al., 2009a, 2009b) to search more broadly for functional enrichment in each gene list. Table S7 provides GO analysis output by DAVID for each gene list using all genes with raw expression measurements as background.

### Subpopulation Enrichment and Depletion Analysis

Under the null hypothesis that there is no association between cluster and anatomic site or psoriatic condition, the number of cells from a given site in a given cluster is a hypergeometric random variable. Mathematically, if *X* is this number, then *X* is distributed as follows:

$$P(X=k)=\frac{\left(\begin{array}{c} K\\ k \end{array}\right)\left(\begin{array}{c} N-K\\ n-k \end{array}\right)}{\left(\begin{array}{c} N\\ n \end{array}\right)},$$

where *N* is the total number of cells, *K* is the number of cells belonging to the anatomic site or psoriatic condition, and *n* is the cluster size. We measure the relative enrichment and depletion of a particular site (anatomic or psoriasis) in a particular cluster by the log ratio of the number of observed cells in this cluster to the number expected under the null (hypergeometric) distribution. Significance of the association between each tissue and cluster pair is assessed using Pearson’s chi-square test with Bonferroni correction. See also Figures 2 and 7.

### RNA Fluorescence *In Situ* Hybridization

For RNA fluorescence *in situ* hybridization (FISH) on formalin-fixed, paraffin-embedded (FFPE) tissue sections, 5-mm sections on glass slides were baked for 1 hr at 60^○^C, deparaffinized, treated for target retrieval, and applied with protease. Then the FFPE sections were incubated with RNAscope FISH probes (Advanced Cell Diagnostics) and hybridized sequentially to visualize target RNA signals, according to the RNAscope Fluorescent Multiplex Kit user manual (ACD). Images were obtained using a Zeiss Axio Imager.M2 with a Plan-Apochromat 20×, numerical aperture (NA) = 0.8 objective.

## Supplementary Material

1

9

10

2

3

4

5

6

7

8

## Highlights

- Stereotyped keratinocyte subpopulations modularly comprise human epidermis
- Scalp keratinocytes exhibit an inherent inflammatory transcriptional program
- Myeloid dendritic cells predominate APCs in psoriatic epidermis
- Macrophages represent major APCs in foreskin epidermis
