# Supplementary material for: Transcriptional Programming of Normal and Inflamed Human Epidermis at Single-Cell Resolution
Source: Cell Rep. Author manuscript; Available in PMC 2019 Feb 7. (PMC6367716; doi:10.1016/j.celrep.2018.09.006)

Cell Reports, Volume *25*

Supplemental Information

Transcriptional Programming of Normal and Inflamed Human Epidermis at Single-Cell Resolution

Jeffrey B. Cheng, Andrew J. Sedgewick, Alex I. Finnegan, Paymann Harirchian, Jerry Lee, Sunjong Kwon, Marlys S. Fassett, Justin Golovato, Matthew Gray, Ruby Ghadially, Wilson Liao, Bethany E. Perez White, Theodora M. Mauro, Thaddeus Mully, Esther A. Kim, Hani Sbitany, Isaac M. Neuhaus, Roy C. Grekin, Siegrid S. Yu, Joe W. Gray, Elizabeth Purdom, Ralf Paus, Charles J. Vaske, Stephen C. Benz, Jun S. Song, and Raymond J. Cho

Supplemental Figures

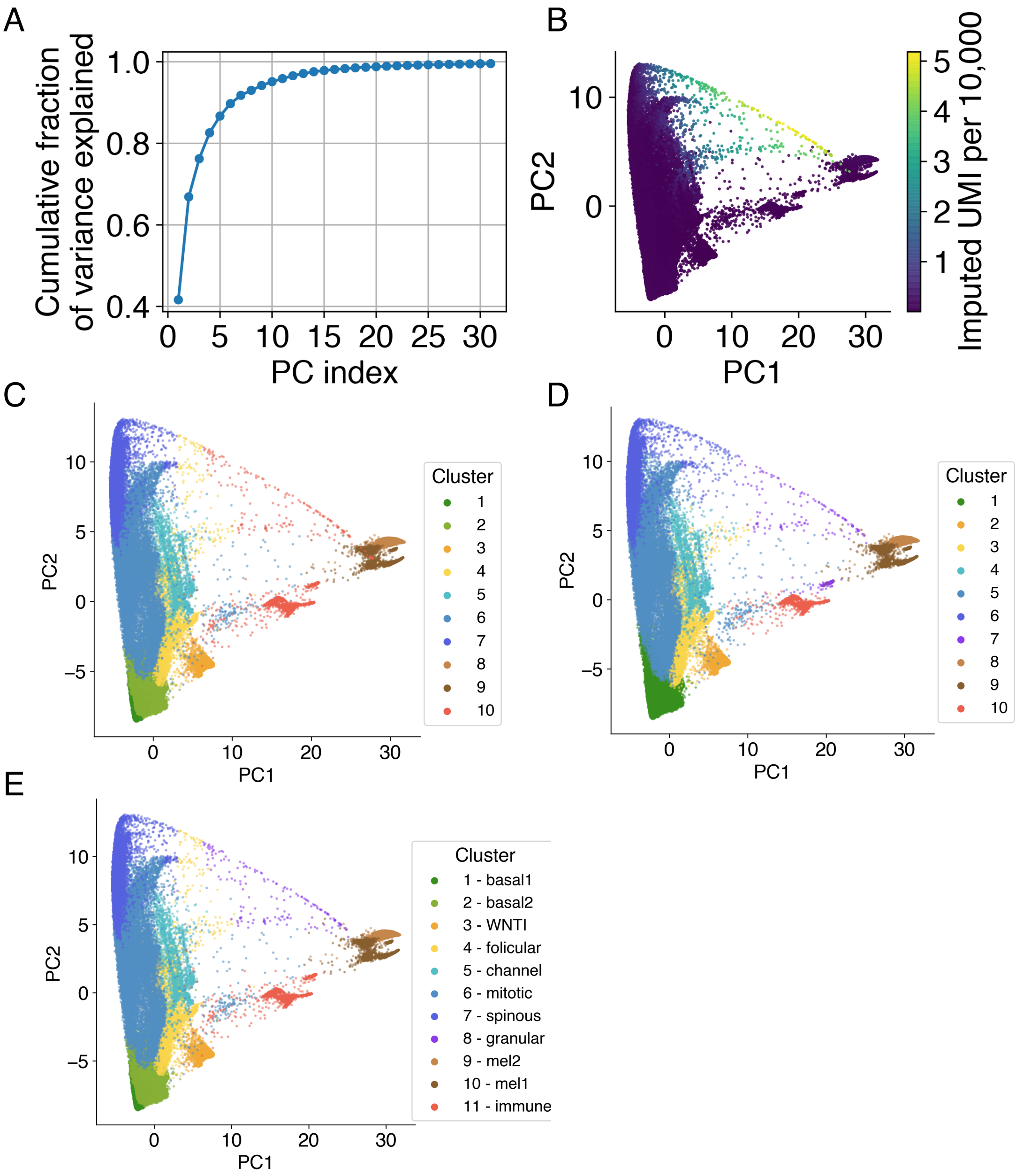

Figure S1 Spectral Clustering Details, Related to Figure 1
Details on spectral clustering of imputed expression. (A) Cumulative fraction of variance explained in PCA analysis of log transformed expression of robustly expressed genes in healthy tissue. (B) Principal components plot colored by imputed expression of late keratinocyte differentiation marker LOR. (C) Approximate spectral clustering with adaptive distance parameters ka=10 and k=30 combines late keratinocytes and immune cells in cluster 10. (D) Approximate spectral clustering with adaptive distance parameters ka=3 and k=10, favoring a more local notion of cell similarity, separates late keratinocytes and immune cells. (E) Principal components plot colored by cluster after separating panel C cluster 10 with second round of spectral clustering.

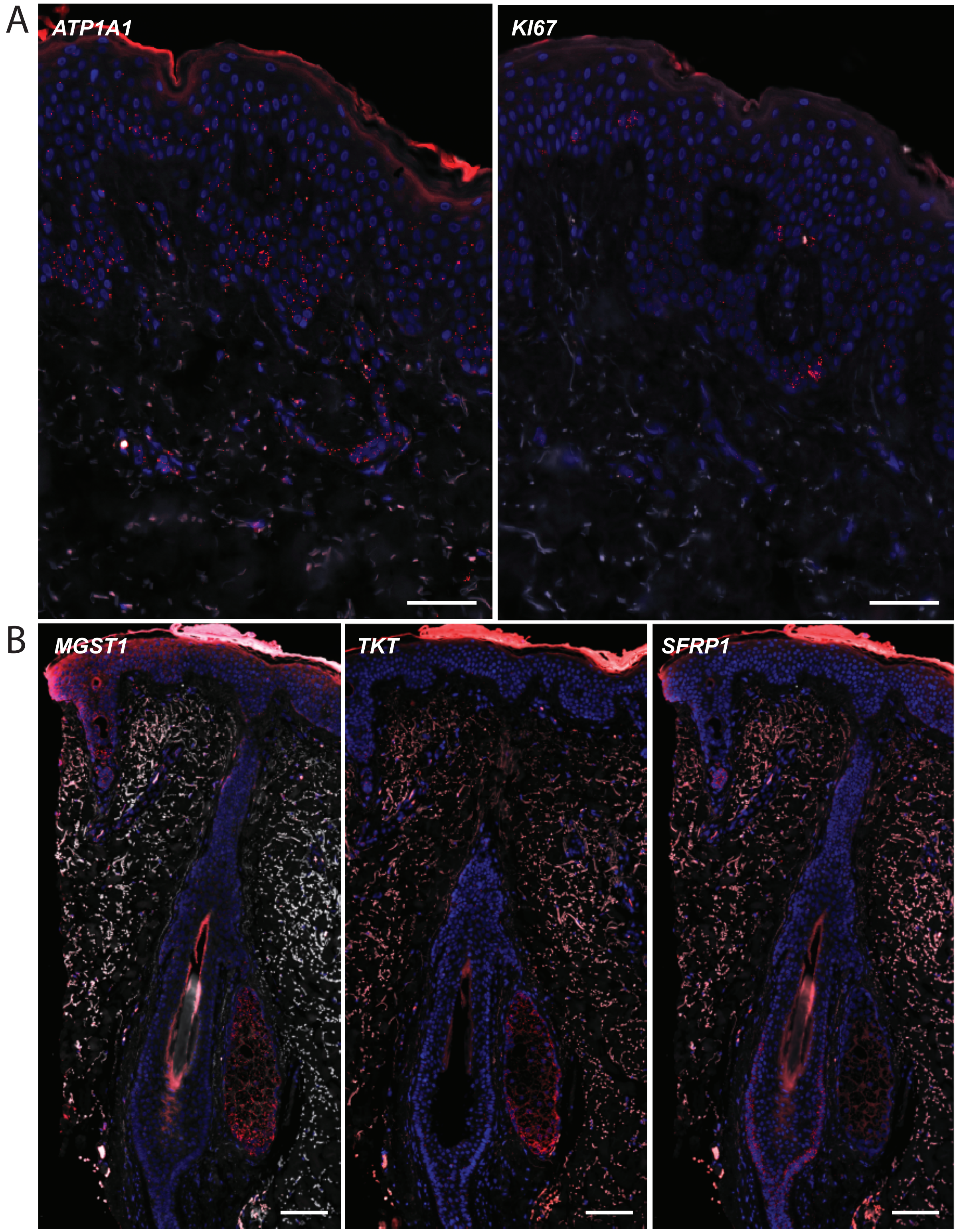

Figure S2 RNA *in situ* hybridization, Related to Figure 1, 3, and 4
(A) RNA *in situ hybridization* staining of trunk skin for *KI67* and *ATP1A1* (red channel). (B) RNA *in situ hybridization* staining (red channel) of scalp skin for hair subpopulation markers *MGST1, TKT,* and *SFRP1.* Blue channel is DAPI staining. Scale bars, 50 µM for A and 100 µM for B.

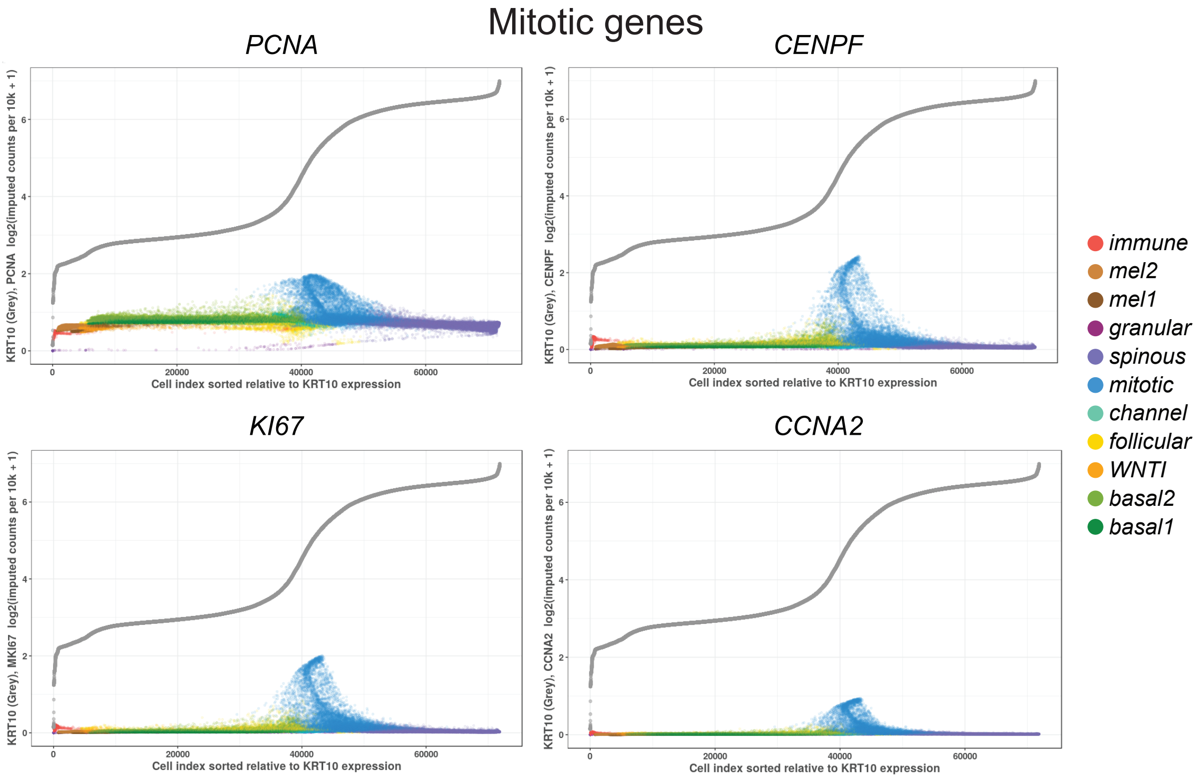

Figure S3 Variance of mitotic and potential progenitor genes with *KRT10*, Related to Figure 3
Imputed expression of mitotic cell cycle genes (*PCNA*, *CENPF*, *KI67*, and *CCNA2*) pseudocolored by cluster and plotted with *KRT10* imputed expression in grey (both expression values on y-axis). Cells are indexed along the x-axis by low to high *KRT10* expression.

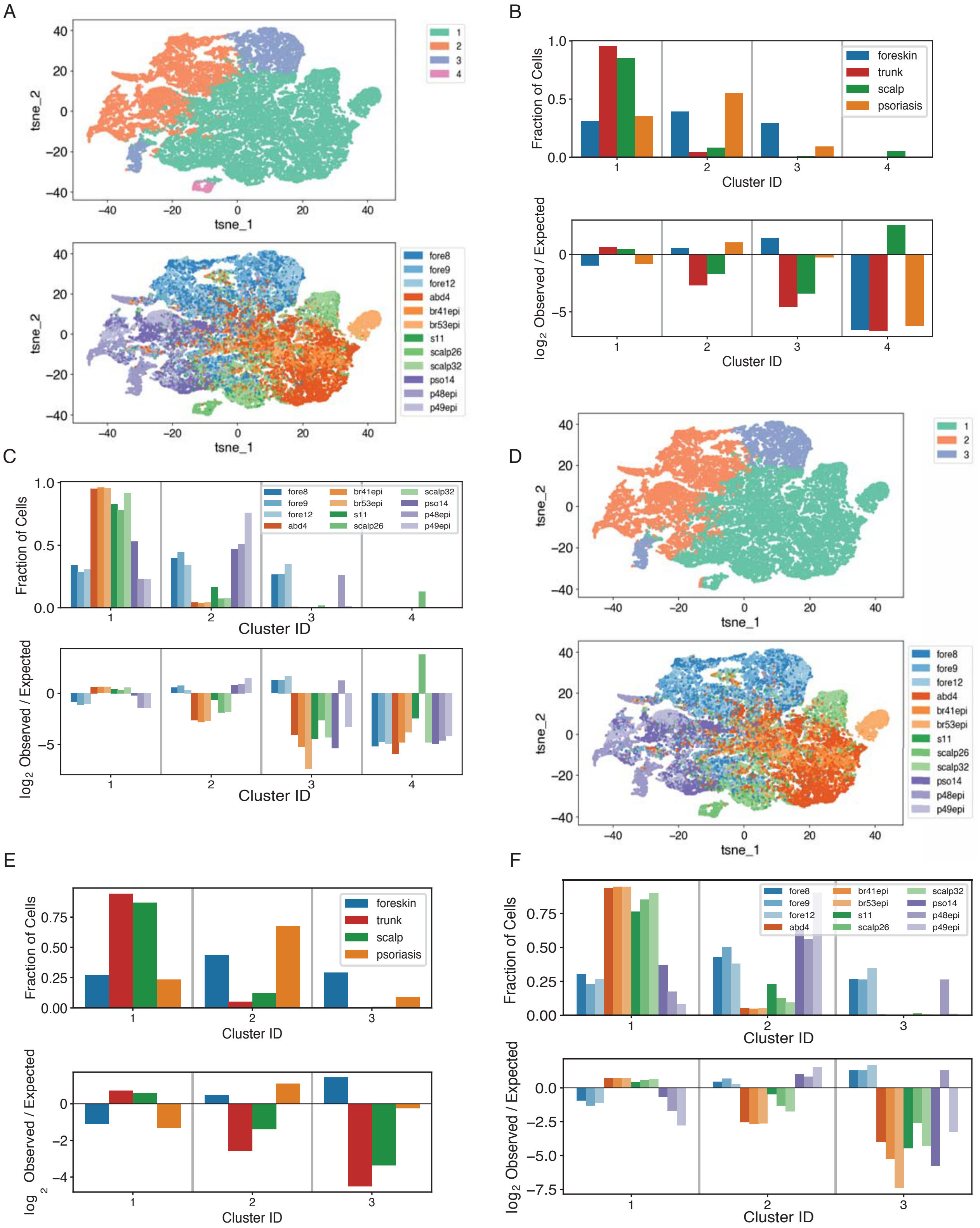
A
B
C
D
E
F
tsne_1
tsne_2
1
2
3
4
fore8
fore9
fore12
abd4
br41epi
br53epi
s11
scalp26
scalp32
pso14
p48epi
p49epi
Fraction of Cells
log2 Observed / Expected
Cluster ID
foreskin
trunk
scalp
psoriasis

Figure S4 Re-clustering of basal keratinocytes into 3 and 4 clusters, Related to Figure 1
(A) tSNE plots showing subclustering of 2 basal clusters (basal1 and basal2) into 4 total clusters (top pseudocolored by cluster, bottom pseudocolored by sample). (B) Fraction of cells from each anatomic site belonging to each cluster by tissue (top) or Log ratio of observed number of cells from anatomic site in cluster to expected number if sampling cells in cluster uniformly without replacement (bottom). Positive/negative log ratios indicate cluster enrichment/depletion for anatomic site or sample. Pseudo-counts of 1 are added to numerator of log-ratios when observed co-occurence is zero. All tissue and cluster associations are significant (padj < 0.05, Person chi square test with Bonferroni adjustment). (C). Same as B except for each individual sample, rather than anatomic site. Note that cluster 4 is almost entirely composed of cells from *scalp26* sample. (D-F) same as (A-C) except showing subclustering into 3 total clusters.

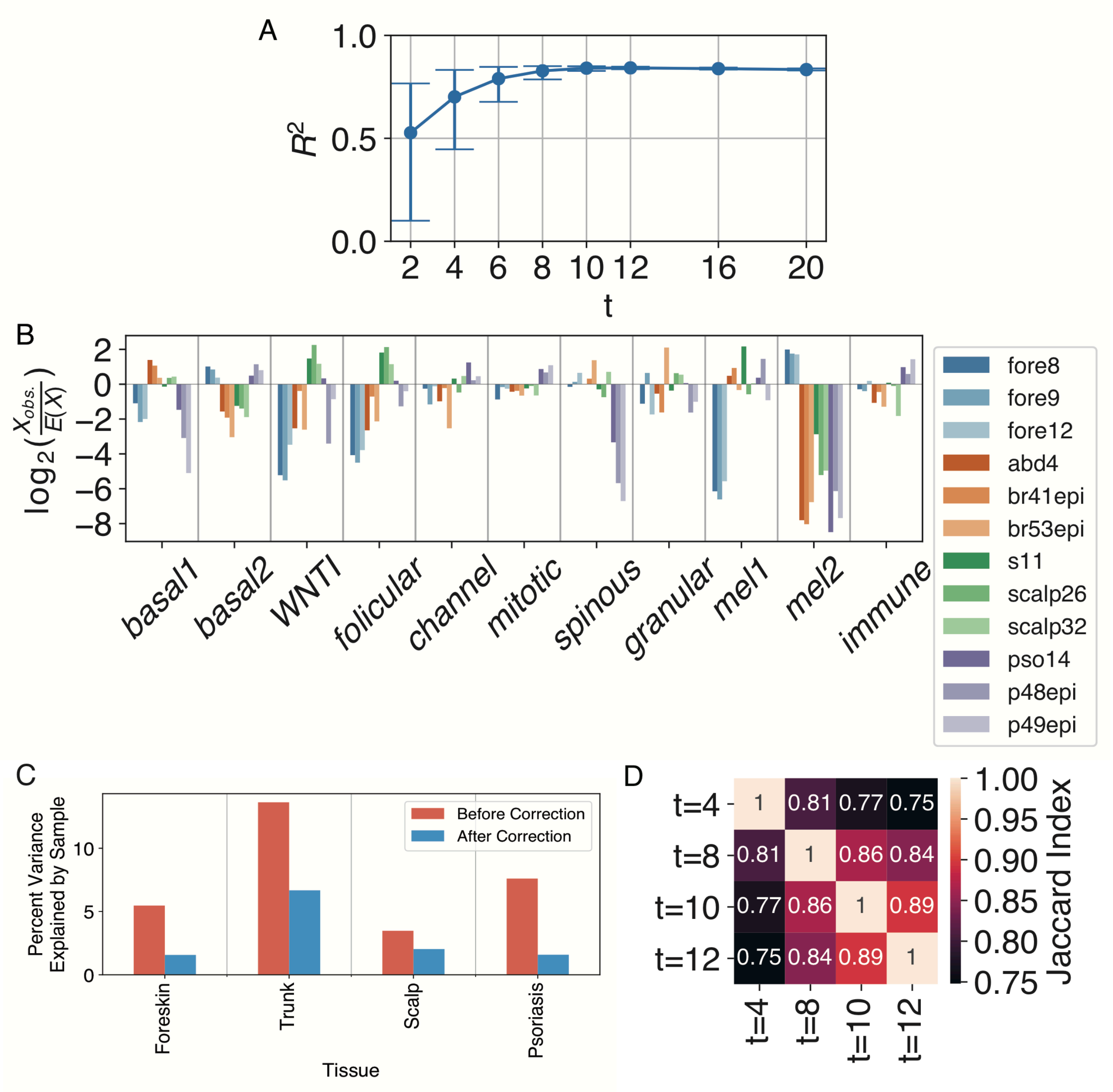

Figure S5 Choice of MAGIC t parameter and variance explained by batch, Related to Figure 1
Choice of MAGIC t parameter and variance explained by batch before and after correction. (A) Square of Pearson correlation coefficient between artificial dropout targets and imputation results for MAGIC algorithm with PCA based similarity. Each t value is described by min, max and mean for 3 replicates of the artificial dropout procedure. (B) Batches are enriched or depleted in clusters according to their anatomical origin. A pseudo-count of 1 is added to allow illustration of depletion when a batch has zero occurrence in a cluster. (C) Percent of variance in gene expression explained by sample within each tissue type. Percentage is calculated as $R^2 \times 100$, summing total and residual sums of squares over 12,783 genes expressed in more than one percent of cells. We used a similar method to quantify biological variation after batch correction and imputation and found that 10.5% of variation in expression is explained by tissue/disease state. (D) Jaccard Indices measuring agreement between KASP clustering (10 clusters ka=10 , k=30) for range of t values.

Supplemental Experimental Procedures

Data processing and QC filtering

Cellranger (10X genomics version 2.0.2) was used to de-multiplex the raw Illumina data, quantify UMIs and aggregate data from our 12 samples. We used the GRCh38 version 1.2.0 reference transcriptome provided by 10X genomics for quantification. We used the default read depth normalization mode for the aggregation step which subsamples reads from higher-depth samples to match the number of confidently mapped reads per cell across samples. We used Seurat (version 2.2.0) (Macosko et al., 2015) to manage and interact with scRNA expression profiles from 116,497 cells from the 12 libraries produced by our sequencing experiments. We performed filtering on the data using percent mitochondrial UMI and number of transcripts with UMI > 0 as quality control metrics. A high proportion of mitochondrial expression is indicative of cells damaged during isolation so we filtered out cells in the top 5th percentile of proportion of mitochondrial UMI, which corresponded to cells with greater than 9.5% of total UMI accounted for by mitochondrial transcripts. To filter on number of detected transcripts, we removed cells in the bottom 0.5% (fewer than 928 transcripts detected) and top 15% (more than 3441 detected) of cells based on this metric, in order to avoid including partial cells and doublets respectively in our analysis. These filters left 92,889 cells to include in our primary analyses.

Imputation

We used the low dimensional representation of cell expression profiles output by ZINB-WaVE (Risso et al., 2018) (version 1.0.0). This representation was used in a modified version of the MAGIC algorithm (van Dijk et al., 2018), described below, to mitigate the effects of scRNA-seq dropout by sharing expression information among cells with similar corrected expression profiles.

Specifically, we used ZINB-WaVE (version 1.0.0) to model, for each gene in each cell, the mean expression and probability of dropout as functions of cell-level covariates: percent mitochondrial UMI, total UMI, batch, and 20 latent cell-level features learned from the raw expression matrix. With this choice of covariates, ZINB-WaVE fits the model:

$$\begin{aligned} \ln(\mu_{i,j}) &= X\beta_u + \left(V\gamma_\mu\right)^T + W\alpha_\mu \\ \text{logit}(\pi_{i,j}) &= X\beta_\pi + (V\gamma_\pi)^T + W\alpha_\pi \\ \ln\left(\theta_{i,j}\right) &= \zeta_j, \end{aligned}$$

where, adopting the notation of Risso et al. (2018), $\mu_{i,j}$ and $\pi_{i,j}$ are, respectively, the mean of the negative binomial distribution and the inflated probability of zero expression for the jth gene ($j \in \{1, \ldots, J\}$) in the ith cell ($i \in \{1, \ldots, n\}$), X is an (n , 14) matrix encoding the known cell level covariates and including a column of 1's, V is a (J, 1) matrix of 1's, and W is a (n, 20) matrix of latent cell-level features. The matrices β_μ , β_π , γ_μ and γ_π with shapes (14, J), (14, J), (1, n), (1, n), respectively, are regression parameters and, α_μ and α_π are (20, J) loading matrices associated with W. Finally, $\theta_{i,j}$ is the inverse dispersion parameter for the negative binomial distribution described by the single parameter ζ_j for each gene.

Covariates in matrices X and V explained unwanted sources of variation in measured gene expression across cells. The matrix W captured the remaining low dimensional variation of interest, with each row providing a 20-dimensional description of a cell's expression state. These rows were used to calculate cell-cell similarities needed to impute expression using the MAGIC algorithm.

Our version of MAGIC, modified to include only wanted sources of variation captured by ZINB-WaVE, is as follows. First, we constructed the MAGIC affinity matrix, using adaptive distance parameters ka = 10 and k = 30 and measuring cell-cell distances with a Euclidean metric on the corresponding rows of the ZINB-WaVE W matrix. That is, we simply replaced the principal component coordinates in the typical MAGIC affinity matrix construction with entries of the ZINB-WaVE W matrix. This custom affinity matrix and the (n, J) matrix R of raw counts were then input to a protocol exactly following the MAGIC algorithm (version 0.0).

Specifically, we calculated the library size normalized raw expression matrix D as

$$D = \frac{R_{i,j}}{\sum_{j=1}^{J} R_{i,j}} \operatorname*{median}_i \left(\sum_{j=1}^{J} R_{i,j}\right)$$

and formed the Markov affinity matrix M by row normalizing the affinity matrix constructed from W. From these matrices, imputed expression is given by

$$D_{imputed} = M^t D$$

where the “diffusion time” t is an integer chosen to yield good recovery of simulated drop-out events on our data set (see Supplementary Experimental Procedures: Choice of MAGIC t parameter”). Finally, the MAGIC protocol dictates rescaling the imputed expression values via:

$$(D_{rescale})_{i,j} = (D_{imputed})_{i,j} \frac{\underset{i}{\text{percentile}}(D_{i,j}, 99)}{\max\limits_{i}((D_{imputed})_{i,j})};$$

that is, imputed expression values were rescaled so that the max imputed expression for each gene matched the 99th percentile of the pre-imputed expression. The matrix $D_{rescale}$ is the imputed result of the MAGIC protocol; however, because it is not guaranteed to be normalized to a common cell library size, we renormalize each row of $D_{rescale}$ to units of imputed expression per 10,000. The resulting expression matrix E, with genes in columns and cells in rows, is the output of our imputation procedure.

Principal component analysis

We used principal component analysis (PCA) on imputed expression of robustly expressed genes to obtain a low dimensional representation of cells according to coordinated variation in gene expression. Specifically, we first restricted to columns of E corresponding to genes robustly expressed in the raw data (>= 5 UMI in >= 100 cells). We then partitioned the resulting matrix, with 2468 genes, into sub-matrices E_{health} corresponding to cells from foreskin, scalp and trunk samples, and E_{pso} corresponding to cells from psoriasis samples. We $\log_2$ transformed entries of E_{health} (with pseudo-count 1) mean centered genes and performed PCA, describing healthy cells by their coordinates along the first 20 principal components (PCs). These PCs sufficed to capture nearly all the variation in our imputed data (Figure S1A). To represent psoriasis cells in this space, we applied the same $\log_2$ transformation and constant shift and projected the transformed psoriasis cell expression vectors onto the 20 PCs. The resulting 20-dimensional cell representations were used as input for t-SNE and multi-tissue clustering of cells.

Spectral clustering

We used spectral clustering of healthy cells represented in the aforementioned 20 PC space to identify similar phenotypic states. Spectral clustering uses eigen-decomposition of a cell-cell similarity matrix to group cells in a manner that depends locally on distance but globally on the distribution of cells in the ambient expression space. Thus, spectral clustering is less biased towards ellipsoidal clusters often identified by purely distance-based clustering algorithms. To reduce the computational cost of diagonalizing a large cell-cell similarity matrix, we employed a modified form of the k-means-based approximate spectral clustering (KASP) algorithm (Yan et al., 2009). Given a matrix $\mathcal{E}_{health}$, with PC coordinates of cells in rows and shape $(71864, 20)$, the number n_{clust} of clusters, and a data reduction factor, alpha, of 10 -- so that spectral clustering is performed on $\text{floor}(71864/10) = 7186$ observations -- the KASP algorithm is:

1. Select $n_{k-means}$ rows from $\mathcal{E}_{health}$, randomly and without replacement, and perform k-means clustering of theses rows identifying 7186 centroids. (Our implementation used $n_{k-means} = 0.5 \times 71864 = 35932$ and took the best k-means results from 20 centroid initializations)
2. Perform a second round of k-means clustering of all 71864 cells initializing centroids at the best result from step 1.
3. Perform spectral clustering of the 7186 centroids from step 2 and identify n_{clust} clusters.
4. Assign each cell to the spectral cluster label of its corresponding k-means centroid from step 2.

Step 3 requires specification of a similarity matrix and type of graph Laplacian constructed from this matrix. We obtained the similarity matrix among k-means centroids using the same construction as for the MAGIC affinity matrix with Euclidean distance measured between centroid representations in 20-dimensional PC space and adaptive similarity parameters ka = 10 and k = 30. From this similarity matrix we construct the random-walk graph Laplacian and perform the Shi and Malik version of spectral clustering (Shi and Malik, 2000) as described by von Luxburg (2007). After assigning cluster labels to all healthy cells, psoriasis cells are assigned to clusters via scikit-learn’s (version 0.19.0) KNeighborsClassifier fit on healthy cells represented in the 20 PC space and using 10 nearest neighbors with other parameters set to default values (Pedregosa et al., 2011).

We observed that the resulting clustering mixed a small number (approximately 200) of late keratinocytes with the primary immune cell cluster (Figure S1B and C). In addition to this important biological distinction, we observed that making the adaptive cell-cell similarity parameters used for clustering more local (decreasing ka and k from 10 to 3 and from 30 to 10, respectively) yielded clustering that successfully partitioned the problematic cluster (Figure S1D). Given these biological and data-driven motivations, we isolated the cells in the immune cell cluster (Figure

S1C, cluster 10) and used spectral clustering (without the k-means based approximation) to split this cluster in two. The resulting clusters are illustrated in Figure S1E and used throughout the main text.

Secondary clustering of basal keratinocytes, scalp keratinocytes, and immune cells
Secondary spectral clustering was performed on three subsets of cells. The first subset consisted of cells assigned to *basal1* and *basal2* in primary spectral clustering; the second subset consisted of cells from the three scalp samples not assigned to the *mel1*, *mel2*, and *immune* cell clusters; and, the third subset consisted of immune cells from primary spectral clustering. We used the method of Supplemental Experimental Procedures: Principal component analysis, restricted to each of these subsets, to obtain 20-dimensional cell representations. Representations of data in each subset were clustered using the KASP algorithm with parameters alpha=2, ka=10, k=20. Secondary clustering of the *basal1* and *basal2* subset tested n_{clust} between 2 and 10. Secondary clustering of scalp and immune subsets tested n_{clust} = 10, 15, and 20.

Choice of MAGIC t parameter
MAGIC's diffusion time parameter *t* controls the extent of neighbors in the weighted cell-cell similarity graph over which cell expression vectors are averaged to yield imputed expression. Increasing *t* decreases locality and increases the number of similar cells used in the average. Our modified version of the MAGIC algorithm used this averaging method to: first, reduce the effect of dropout in raw data and, second, remove patterns of variation in raw data attributable to unwanted sources. The second objective was achieved by using ZINB-WaVE's W matrix in our construction of the cell-cell similarity graph, so that MAGIC removes potential batch effects by averaging raw expression vectors over cells having similar corrected low-dimensional representations in W.
To choose t, we simulated MAGIC's ability to recover dropout values in our raw expression matrix R. Because the cell-cell similarity information contained in ZINB-WaVE's W matrix was based on the entire data matrix R without simulated dropouts, using the W matrix as an input would over-estimate the true recovery rate. Thus, we instead used the original MAGIC algorithm to choose a value of t yielding good simulated dropout recovery. We used this t in our modified version of MAGIC and verified the absence of batch effect in clustering of the resulting imputed data.
Specifically, we sampled (for computational efficiency) 8,000 foreskin cells at random and formed the raw expression matrix R_{sub} for these cells and for genes with at least 1 UMI in 1% of these cells. We randomly sampled 20% (~3,800,000) of the non-zero entries of R_{sub} as dropout events. Denoting the set of selected (row, column) dropout pairs by S, the targets for imputation recovery were

$$T = \left((D_{sub})_{i,j} : (i,j) \in S\right)$$

where D_{sub} is the library normalized raw expression matrix

$$D_{sub} = \frac{(R_{sub})_{i,j}}{\sum_{j=1}^{J}(R_{sub})_{i,j}} \operatorname*{median}_{i}\left(\sum_{j=1}^{J}(R_{sub})_{i,j}\right).$$

We then set $(R_{sub})_{i,j} = 0$ for all $(i,j) \in S$ and called the resulting corrupted matrix $\mathcal{R}$. We ran the standard MAGIC algorithm on $\mathcal{R}$, calculating the library normalized matrix $\mathcal{D}$ from $\mathcal{R}$ and then constructing the MAGIC affinity matrix from Euclidean distances between cells represented in the space spanned by the 20 highest-variance PCs, where PCA was performed on the $\log_2$ transformed (with pseudo-count 1) and mean centered $\mathcal{D}$ matrix. We measured recovery using R^2 (the square of Pearson correlation coefficient) between the sequences T and $((\mathcal{D}_{rescale})_{i,j}: (i,j) \in S)$, where $\mathcal{D}_{rescale}$ is the output of the MAGIC algorithm (described in Supplemental Experimental Procedures: Imputation). Based on the results in Figure S5A, we identified $t = 10$ as a candidate parameter with good recovery.
To verify that $t = 10$ removed unwanted batch effects in imputed expression, we performed imputation on the full data set using our modified MAGIC algorithm and examined the enrichment/depletion of independent samples in each of the KASP clusters (adaptive distance parameters ka=10, k=30). The samples were enriched or depleted in clusters according to their anatomical origin, rather than experimental batch, implying that the choice of diffusion time $t = 10$ successfully removed unwanted batch artifacts (Figure S5B).
We quantified the batch correction performance by calculating the percentage of variance in gene expression ($R^2 \times 100$) explained by sample within each tissue type, before and after application of the ZINB-WaVE/MAGIC

algorithm (Figure S5C). Quantification via R^2 used total and residual sums of squares taken over 12,783 genes expressed in more than one percent of cells. We used a similar method to quantify biological variation after batch correction and found that 10.5% of variation in expression is explained by tissue/disease state.

Finally, we demonstrated the stability of clustering results against changes in t. We ran our modified MAGIC algorithm on the full data set with $t = 4, 8, 10, 12$, clustered each imputed result into 10 clusters with KASP (same parameters as in Supplemental Experimental Procedures: Spectral clustering) and measured the clustering concordance using the Jaccard index. The Jaccard Index between clusterings A and B ranges between 0 (no agreement) and 1 (perfect agreement) and is the ratio of the number of cell pairs co-clustered in both A and B to the number of cell pairs co-clustered in either A or B. The clustering results did not change appreciably as t was varied between 8 and 12 (Figure S5D).

Pseudotime

We used Slingshot (Street et al., 2018) (version 0.1.2-1) to infer developmental lineages of scalp keratinocytes. Coordinates of scalp cells in the basis of the first 10 PCs obtained in Supplemental Experimental Methods: Principal component analysis were used as input to the Slingshot algorithm. We specified the basal1 cluster as the starting state, and the granular cluster as a terminal state. We then used the pseudotime prediction to order all cells in all three scalp samples along the predicted differentiation trajectory to each terminal cluster.

tSNE mapping

We used Rtsne (Krijthe, 2015; Maaten, 2008) (version 0.13) to perform T-distributed stochastic neighbor embedding (tSNE) on the 20-dimensional representation of cells described in Experimental Procedures: Principal component analysis. tSNE used parameters theta = .5 and perplexity = 40.

Processing time and sex-specific bias analysis

To assess for possible confounding effects of processing time and male/female gene expression differences, we compared 4 samples to our 9 core normal epidermis samples (abd4, br41epi, br53epi, fore12, fore8, fore9, s11, scalp26, and scalp32). Two additional trunk samples were processed in less than 2 hours (vs. median of 22 hours for samples in the 9 core samples), in order to control for variances in processing time. Two additional male trunk samples were also collected, rather than the female trunk samples in our core series. These 4 samples were normalized and imputed together with cells from our 9 core normal samples. The additional 4 samples were then projected onto the 11 clusters identified from the core 9 samples using the same PCA-based nearest neighbor method and settings (scikit-learn's KNeighborsClassifier) used to map the psoriasis samples.

The silhouette statistic (Rousseeuw, 1987) was then used to assess whether cells in these four new samples clustered differently from our original samples. The silhouette statistic compares the average principal component distance between a cell and A) other cells of its same group vs. B) cells of any other group. Tissue type and cluster represent types of groups. Lower average distance within cells of the same group, relative to cells of the closest other group, corresponds to a positive silhouette, with a maximum of 1. Higher average distance within cells of the same group, relative to cells of the closest other group, corresponds to a negative silhouette, with a minimum of -1 (and indicates that a cell would fit better in the other group). A silhouette near 0 means that a cell has a similar average distance to two groups. Averaging the silhouette statistic across cells can give an overall sense of whether the assessed groupings represents tight, well separated sets (average silhouette approaching 1) or heterogeneous groups where cells don't clearly fall within a single group (average silhouette around 0).

In column 2 of the table below (labelled tissue), we show the silhouette statistic calculated on the tissue groups within each cluster, averaged across all the cells of each tissue**.** Here, the silhouette statistic shows that in clusters, cells demonstrate similar distance between other cells in their tissue of origin and the next closest tissue (averaged values are neither close to 1 or -1 but close to 0). This indicates that cells in a given cluster are not highly related by tissue type. For the 2-hour and male samples, this indicates that time or sex-specific transcriptional signal causes relatively minor confounding segregation, even within discrete clusters.

In column 3 of the table below (labelled cluster), we show the silhouette statistic calculated on the cluster groups within each tissue, averaged across the cells of each tissue. The relatively high values across this analysis show that cells of a given cluster are tightly related, regardless of tissue source. The 2-hour and male samples show similar average silhouettes to the originally analyzed tissue types, indicating that any time or sex-specific transcriptional signal does not substantially impair how robustly the clusters segregate cells.

	tissue	cluster
2 hour	-0.08617	0.374327
male	-0.08449	0.382997
foreskin	0.075526	0.297912
trunk	0.031249	0.408574
scalp	-0.01125	0.335295

Supplemental References

Krijthe, J.H. (2015). Rtsne: T-Distributed Stochastic Neighbor Embedding using a Barnes-Hut Implementation.

Maaten, L. (2008). Visualizing data using t-SNE. The Journal of Machine Learning Research *9*, 2579.

Macosko, E.Z., Basu, A., Satija, R., Nemesh, J., Shekhar, K., Goldman, M., Tirosh, I., Bialas, A.R., Kamitaki, N., Martersteck, E.M.*, et al.* (2015). Highly Parallel Genome-wide Expression Profiling of Individual Cells Using Nanoliter Droplets. Cell *161*, 1202-1214.

Pedregosa, F., Varoquaux, G., Gramfort, A., Michel, V., Thirion, B., Grisel, O., Blondel, M., Prettenhofer, P., Weiss, R., Dubourg, V.*, et al.* (2011). Scikit-learn: Machine Learning in Python. J Mach Learn Res *12*, 2825-2830.

Risso, D., Perraudeau, F., Gribkova, S., Dudoit, S., and Vert, J.P. (2018). A general and flexible method for signal extraction from single-cell RNA-seq data. Nat Commun *9*, 284.

Rousseeuw, P.J. (1987). Silhouettes: A graphical aid to the interpretation and validation of cluster analysis. Journal of Computational and Applied Mathematics *20*, 53-65.

Shi, J.B., and Malik, J. (2000). Normalized cuts and image segmentation. Ieee T Pattern Anal *22*, 888-905.

Street, K., Risso, D., Fletcher, R.B., Das, D., Ngai, J., Yosef, N., Purdom, E., and Dudoit, S. (2018). Slingshot: cell lineage and pseudotime inference for single-cell transcriptomics. BMC Genomics *19*, 477.

van Dijk, D., Sharma, R., Nainys, J., Yim, K., Kathail, P., Carr, A.J., Burdziak, C., Moon, K.R., Chaffer, C.L., Pattabiraman, D.*, et al.* (2018). Recovering Gene Interactions from Single-Cell Data Using Data Diffusion. Cell *174*, 716-729 e727.

von Luxburg, U. (2007). A tutorial on spectral clustering. Statistics and Computing *17*, 395-416.

Yan, D.H., Huang, L., and Jordan, M.I. (2009). Fast Approximate Spectral Clustering. Kdd-09: 15th Acm Sigkdd Conference on Knowledge Discovery and Data Mining, 907-915.

Supplemental Data

Data S1 Per sample representation and marker gene expression in t-SNE plot epidermal clusters, Related to Figure 1, 4, and 6

(A) Trunk-specific tSNE plot (as in Figure 1A) pseudocolored by sample. (B) Foreskin-specific tSNE plot (as in Figure 1A) pseudocolored by sample. (C) Scalp-specific tSNE plot (as in Figure 1A) pseudocolored by sample. (D) 15 scalp-specific cluster tSNE plot (as in Figure 4) pseudocolored by sample. (E). Immune cluster tSNE plot (as in Figure 6) pseudocolored by sample. (F) tSNE plot incorporating all 9 normal samples (G) Expression pattern of epidermal subpopulation marker gene expression across cell clusters in anatomic site-specific tSNE plots.

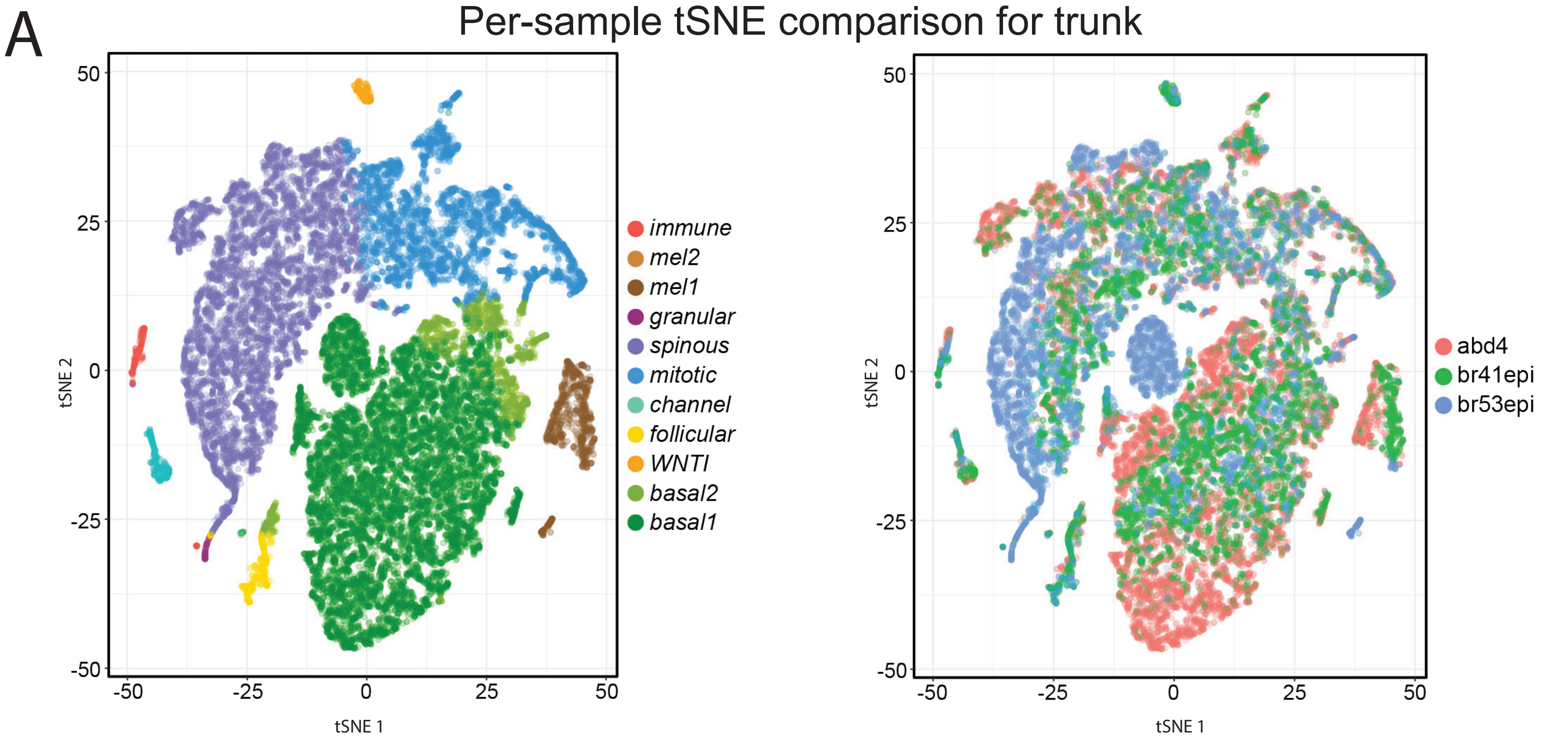

Color-coded by cluster (Fig 1A)

Color-coded by sample

B

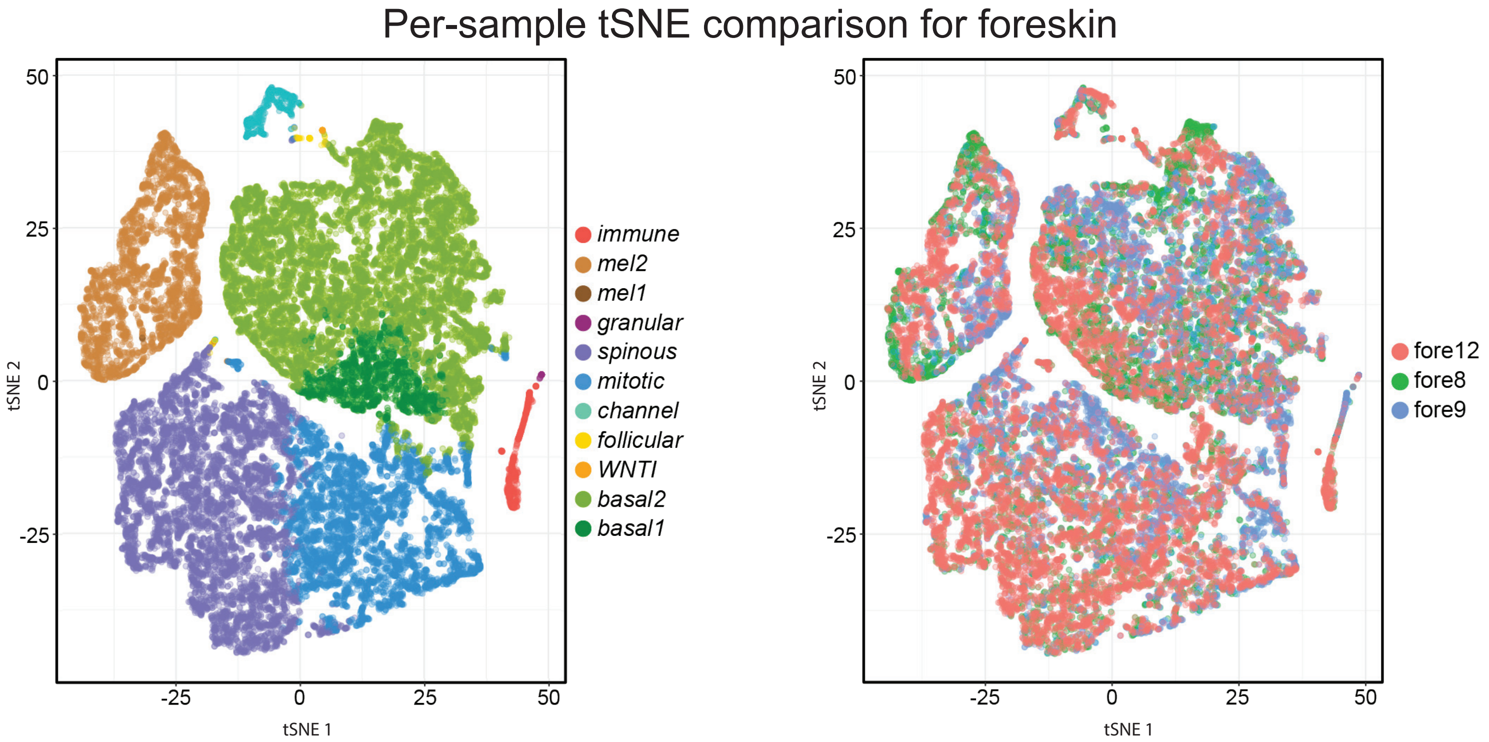

Color-coded by cluster (Fig 1A)

Color-coded by sample

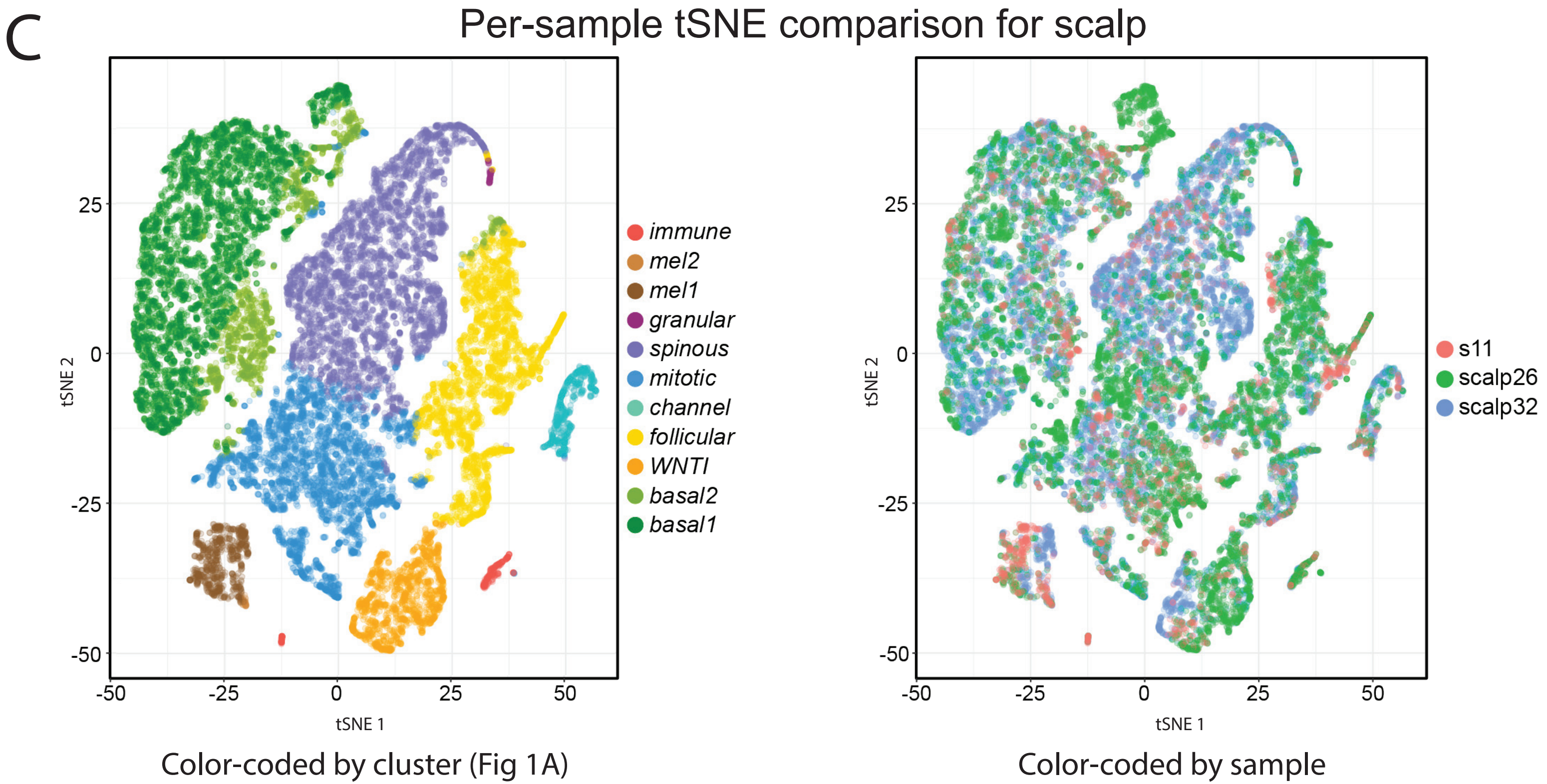
C
Per-sample tSNE comparison for scalp
tSNE 2
tSNE 1
25
0
-25
-50
-50
-25
0
25
50
immune
mel2
mel1
granular
spinous
mitotic
channel
follicular
WNTI
basal2
basal1
s11
scalp26
scalp32
Color-coded by cluster (Fig 1A)
Color-coded by sample

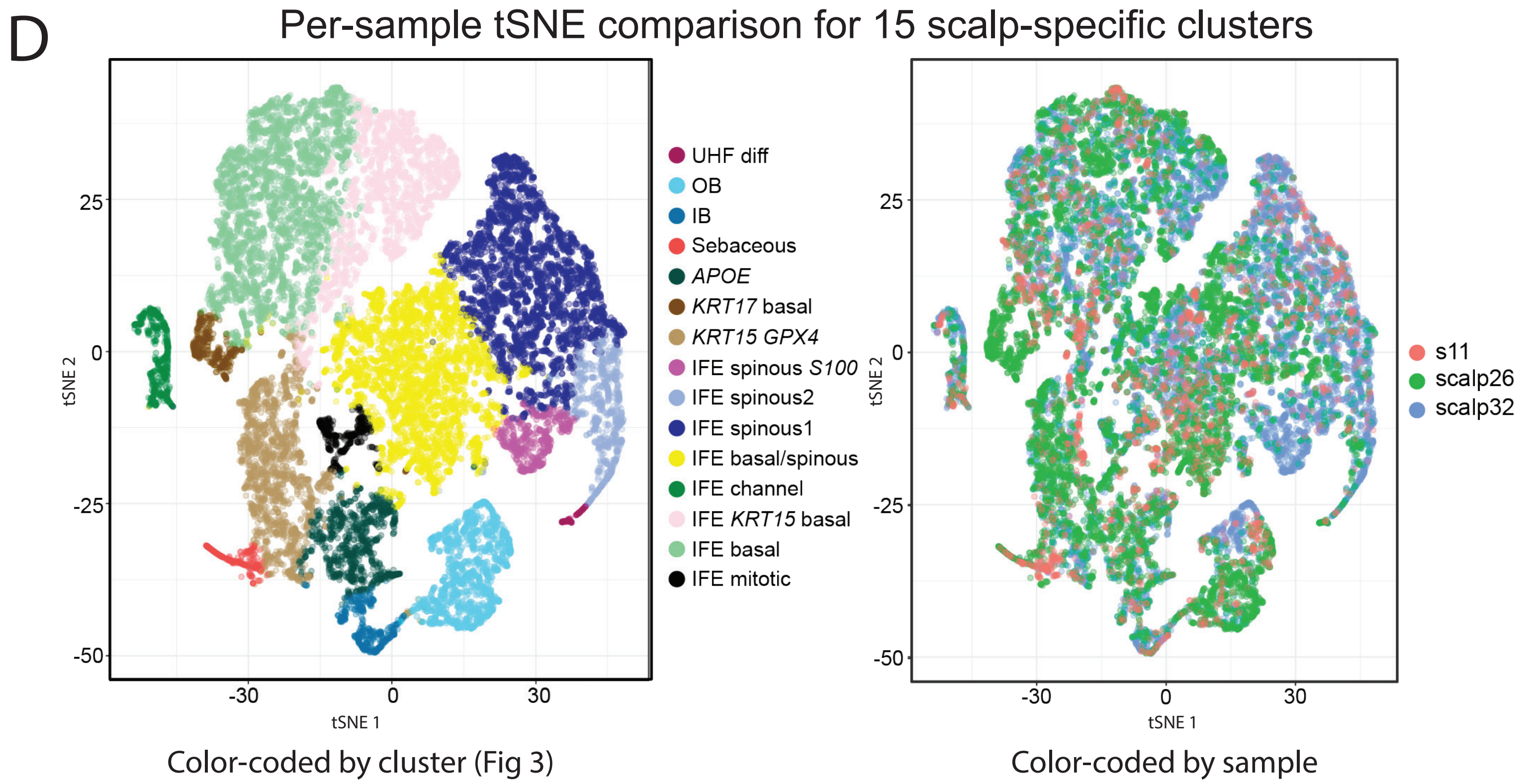
D
Per-sample tSNE comparison for 15 scalp-specific clusters
25
0
-25
-50
tSNE 2
-30
0
30
tSNE 1
UHF diff
OB
IB
Sebaceous
APOE
KRT17 basal
KRT15 GPX4
IFE spinous S100
IFE spinous2
IFE spinous1
IFE basal/spinous
IFE channel
IFE KRT15 basal
IFE basal
IFE mitotic
s11
scalp26
scalp32
Color-coded by cluster (Fig 3)
Color-coded by sample

E

Per-sample tSNE comparison for immune clusters

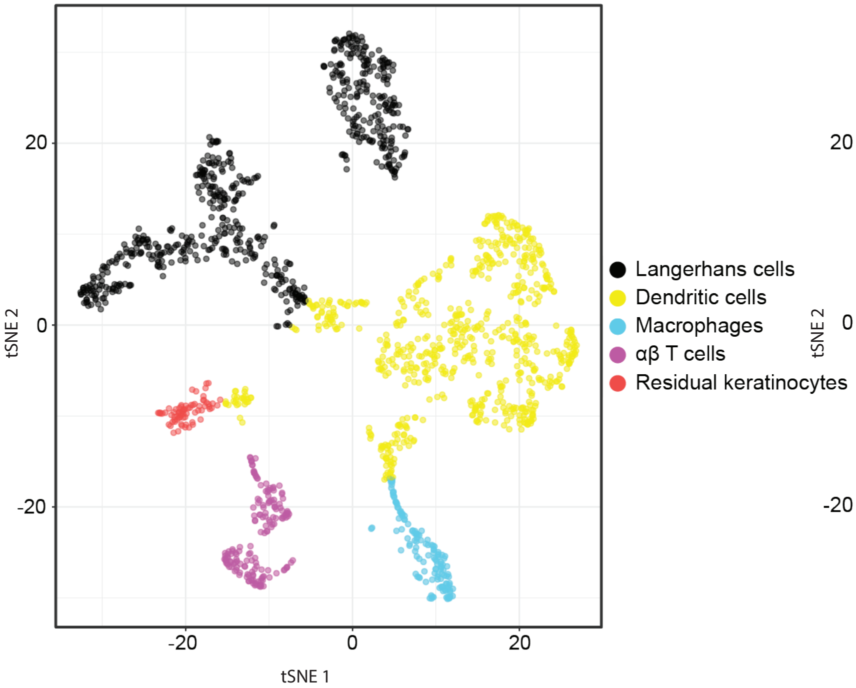

Color-coded by cluster (Fig 6)

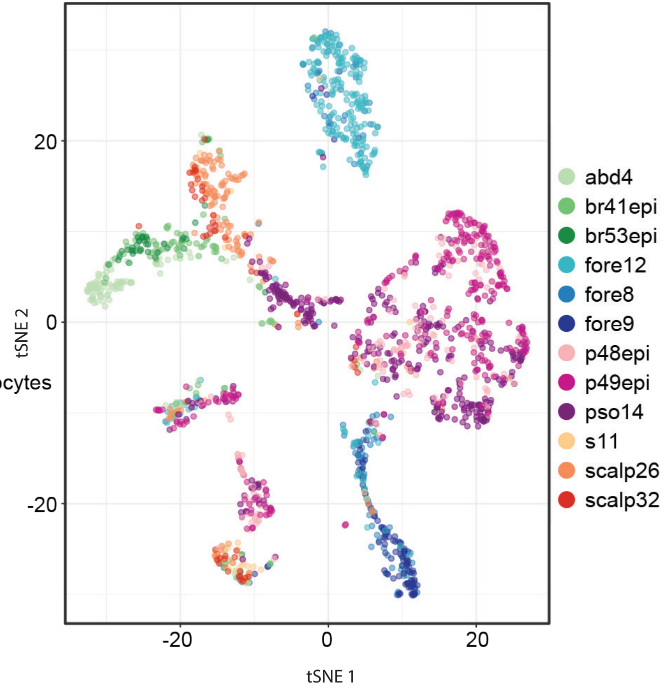

Color-coded by sample

F

Aggregate tSNE of clustering of 9 normal samples

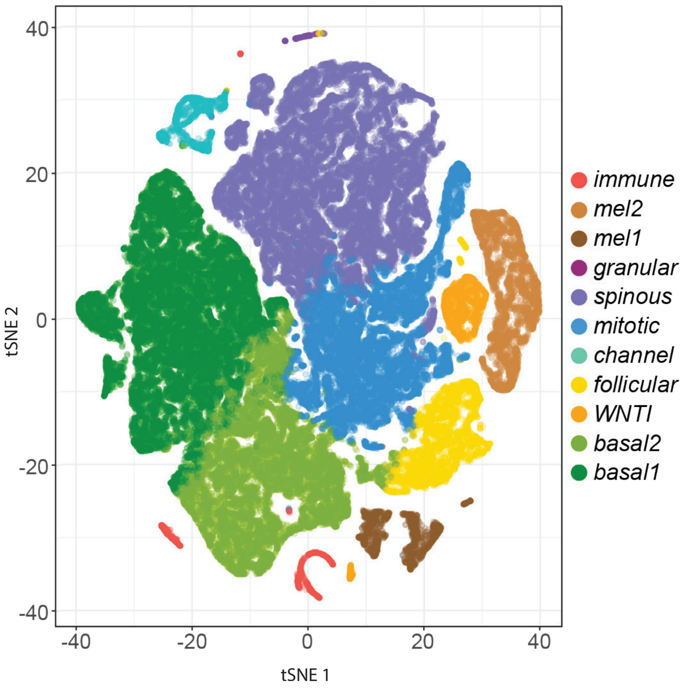

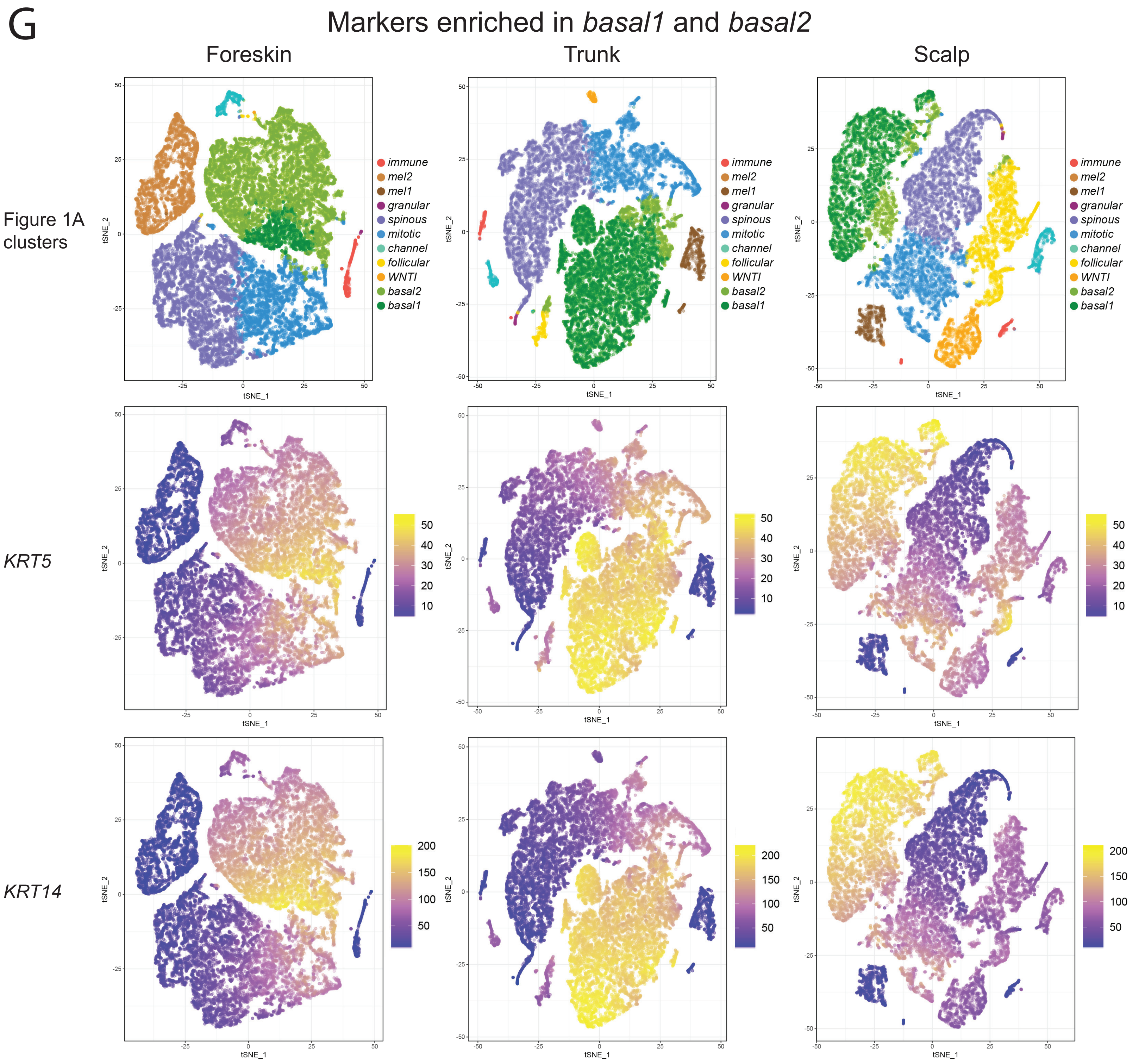
G
Markers enriched in basal1 and basal2
Foreskin
Trunk
Scalp
Figure 1A clusters
KRT5
KRT14
immune
mel2
mel1
granular
spinous
mitotic
channel
follicular
WNT1
basal2
basal1
tSNE_1
tSNE_2

Markers enriched in *spinous*

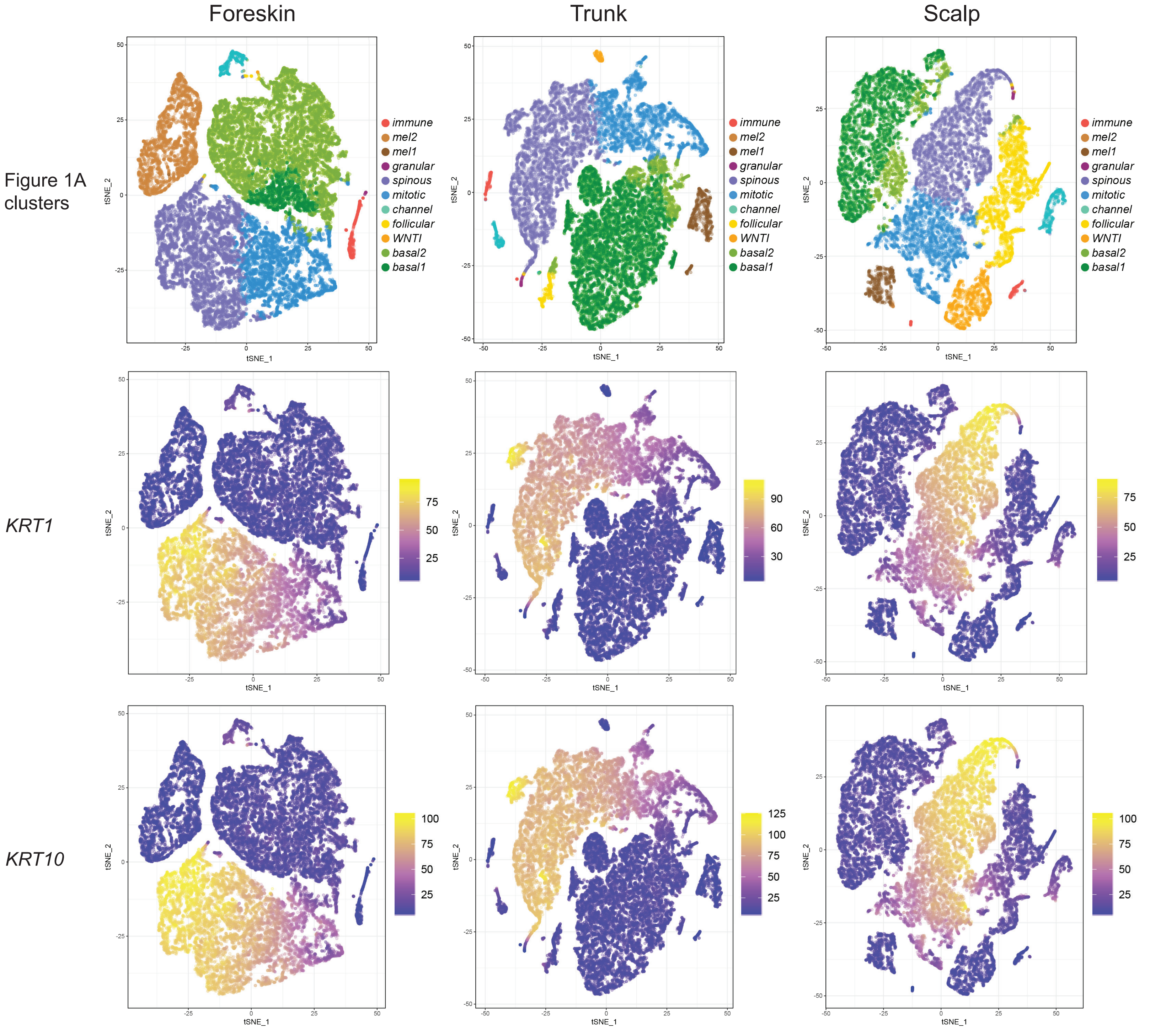

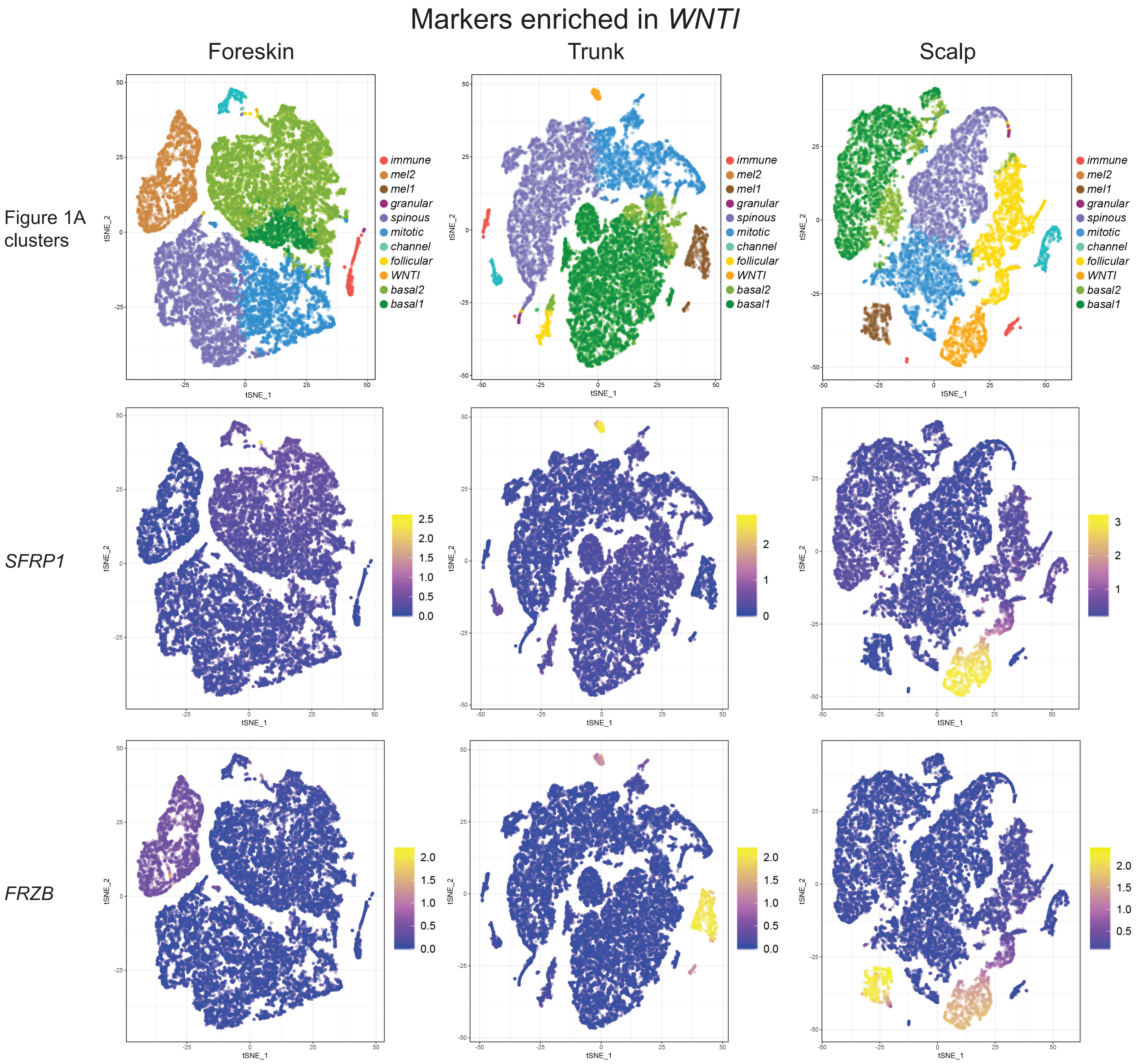

Markers enriched in WNTI
Foreskin
Trunk
Scalp
Figure 1A clusters
SFRP1
FRZB
immune
mel2
mel1
granular
spinous
mitotic
channel
follicular
WNTI
basal2
basal1
tSNE_1
tSNE_2

Markers enriched in *follicular*

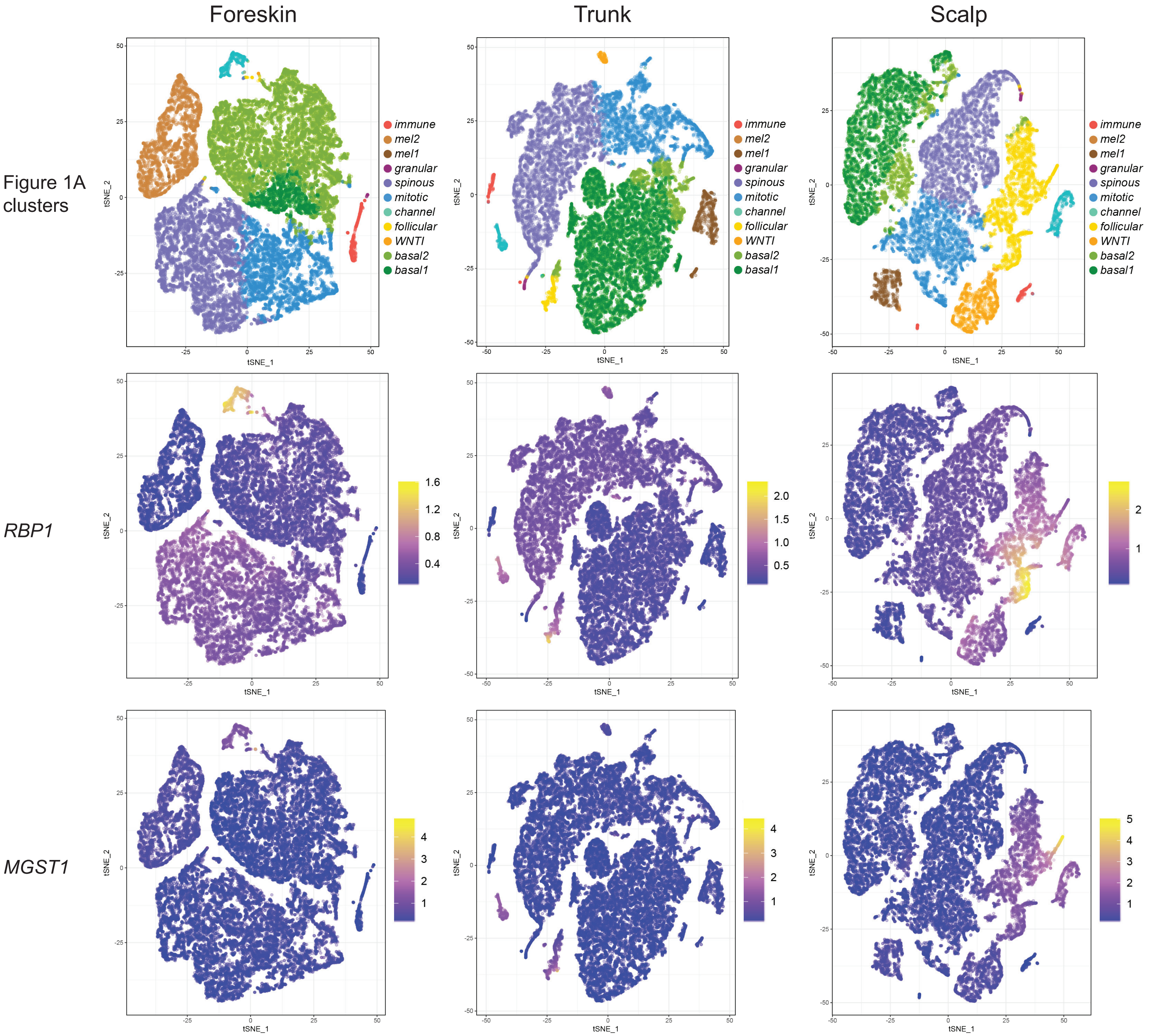

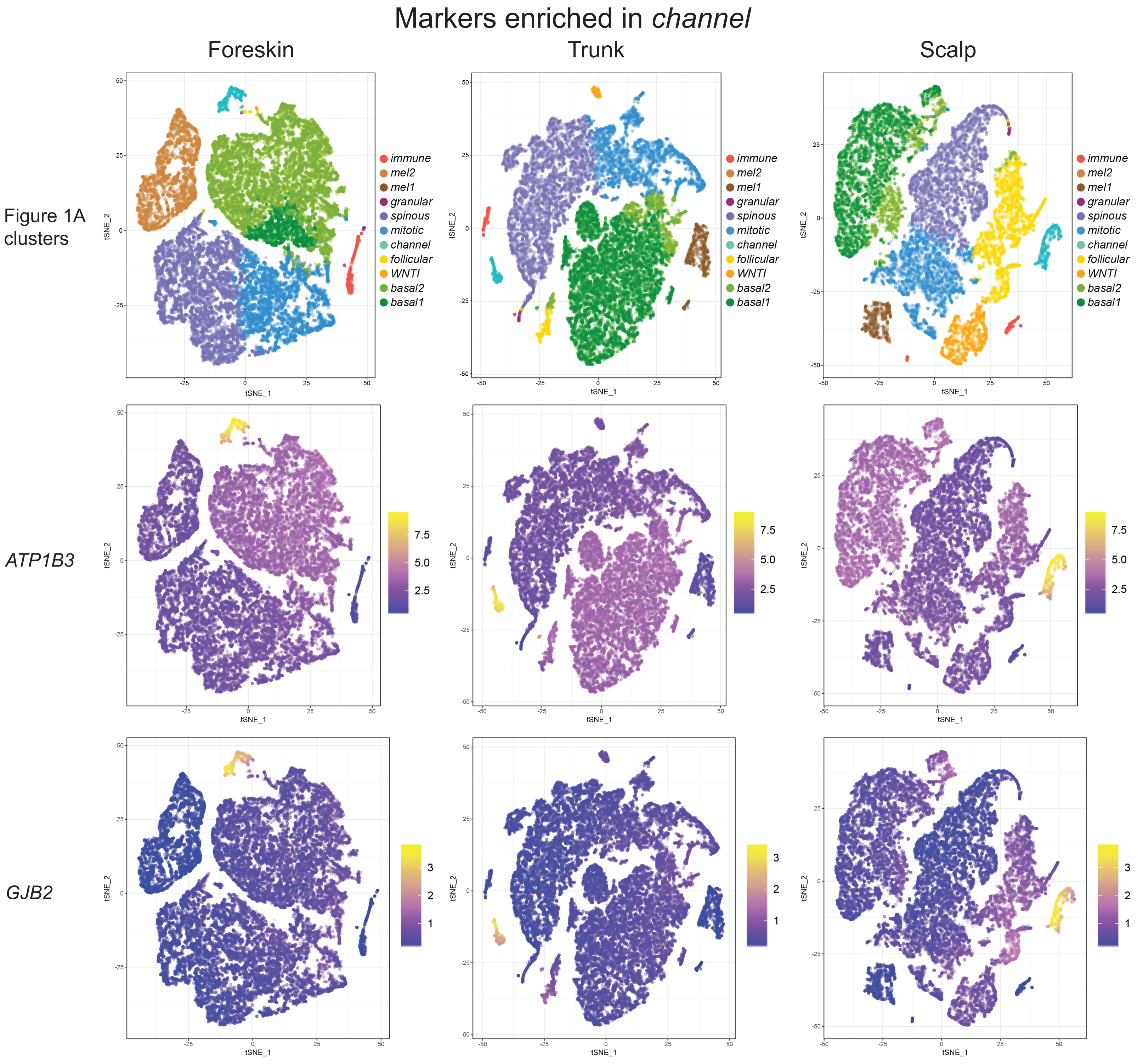

Markers enriched in *channel*
Foreskin
Trunk
Scalp
Figure 1A clusters
ATP1B3
GJB2
immune
mel2
mel1
granular
spinous
mitotic
channel
follicular
WNTI
basal2
basal1
tSNE_1
tSNE_2

Markers enriched in *mitotic*

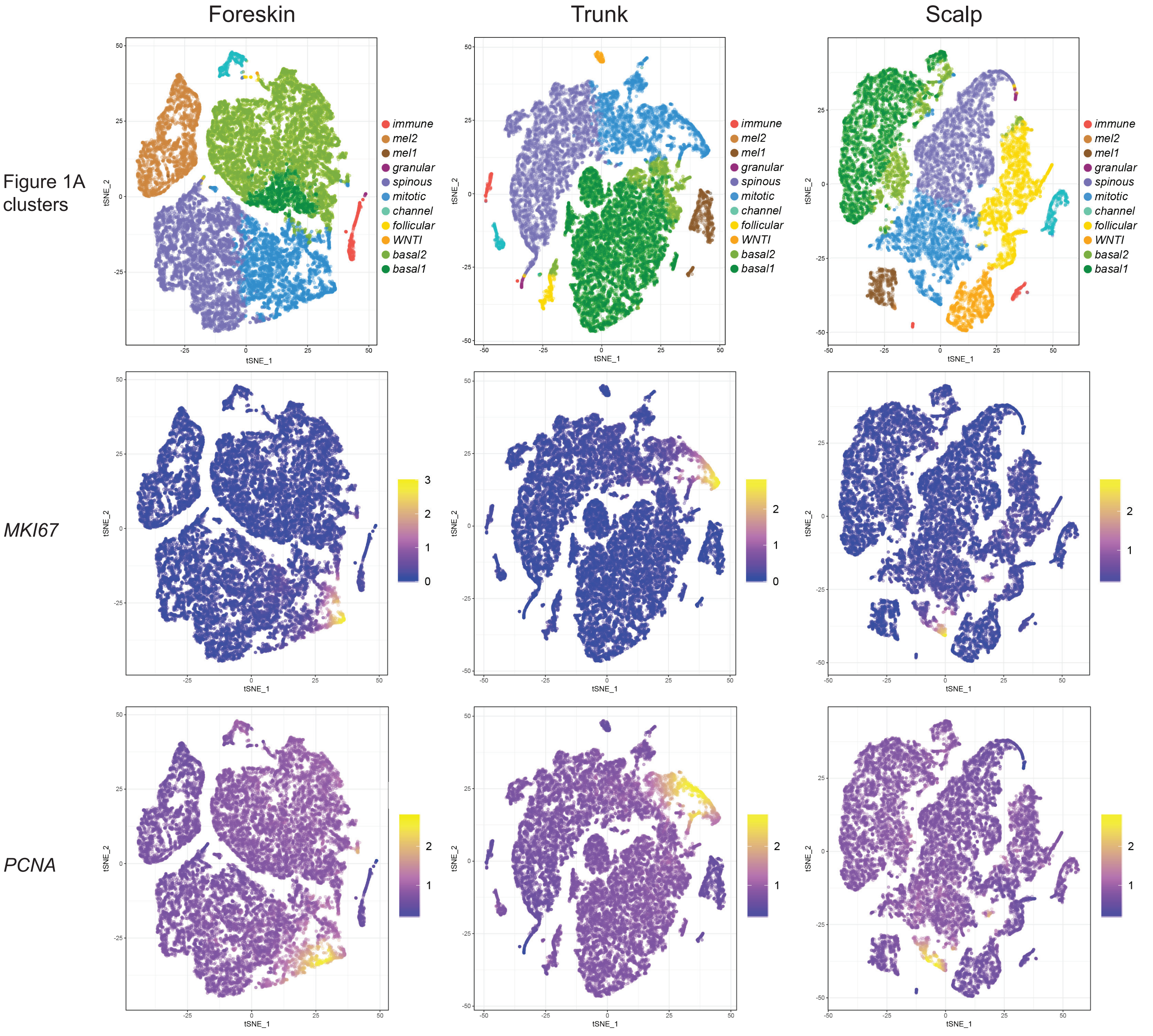

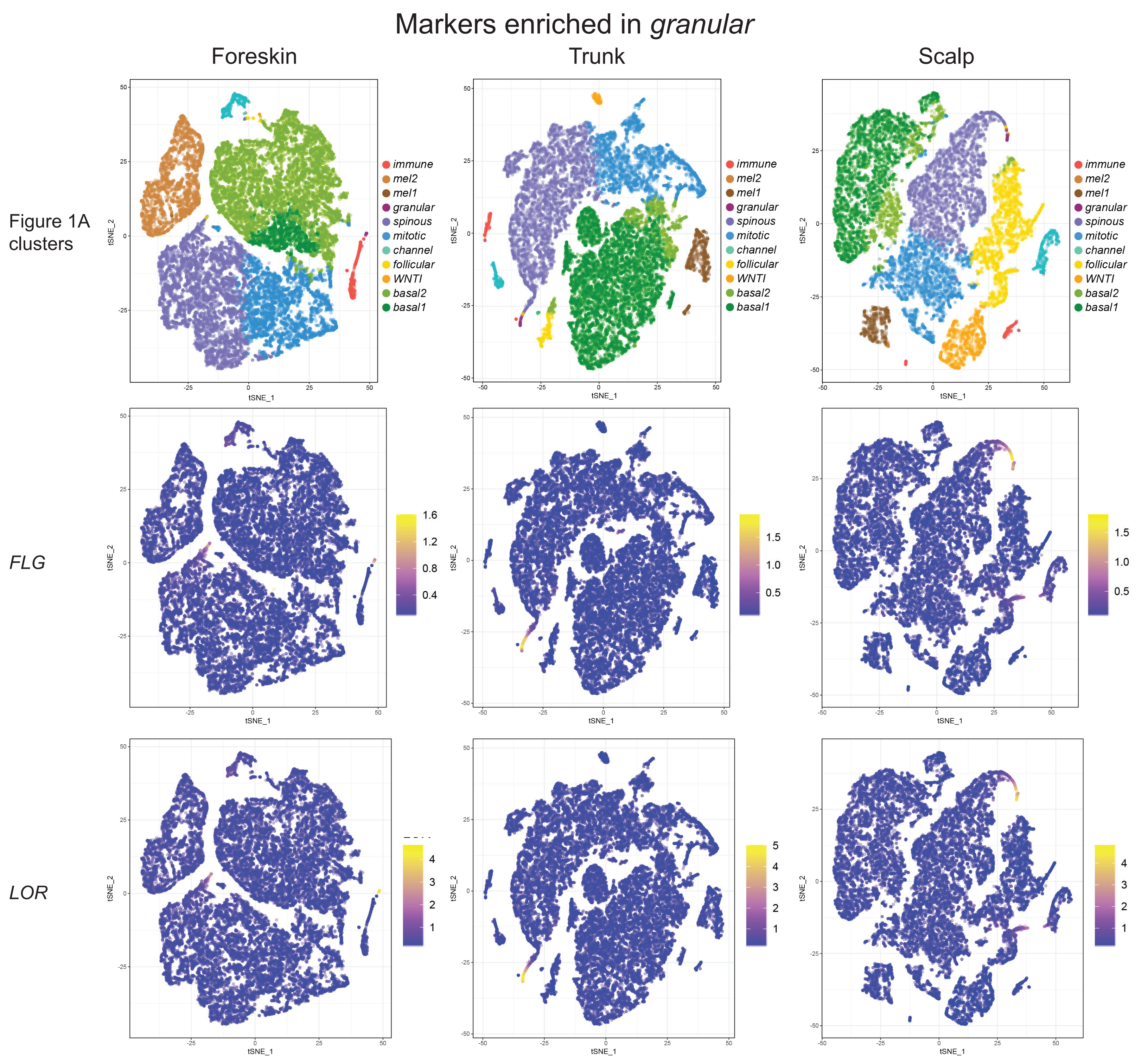
Markers enriched in granular
Foreskin
Trunk
Scalp
Figure 1A clusters
FLG
LOR
immune
mel2
mel1
granular
spinous
mitotic
channel
follicular
WNTI
basal2
basal1
tSNE_1
tSNE_2

Markers enriched in *mel1* and *mel2*

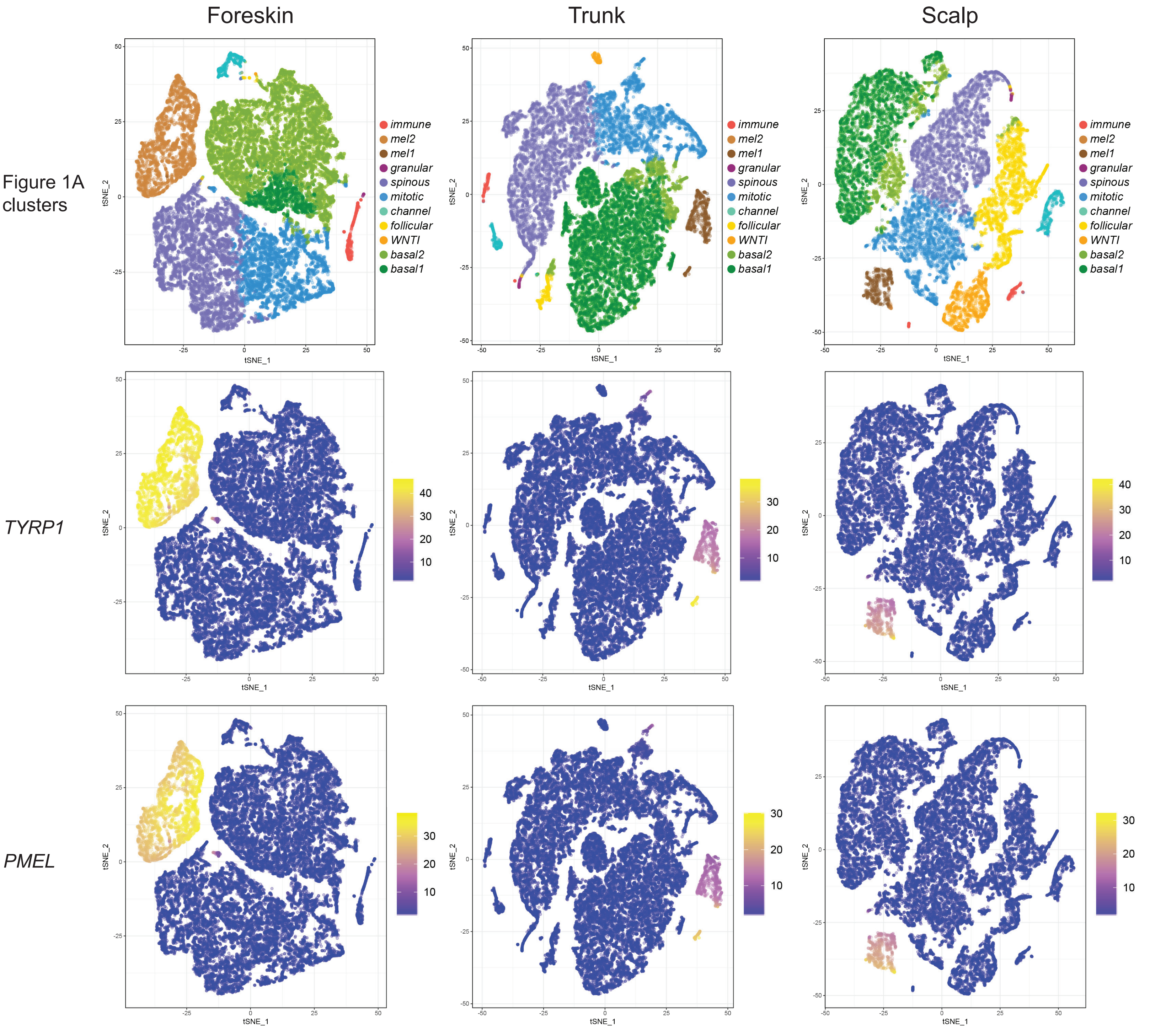

Markers enriched in *immune*

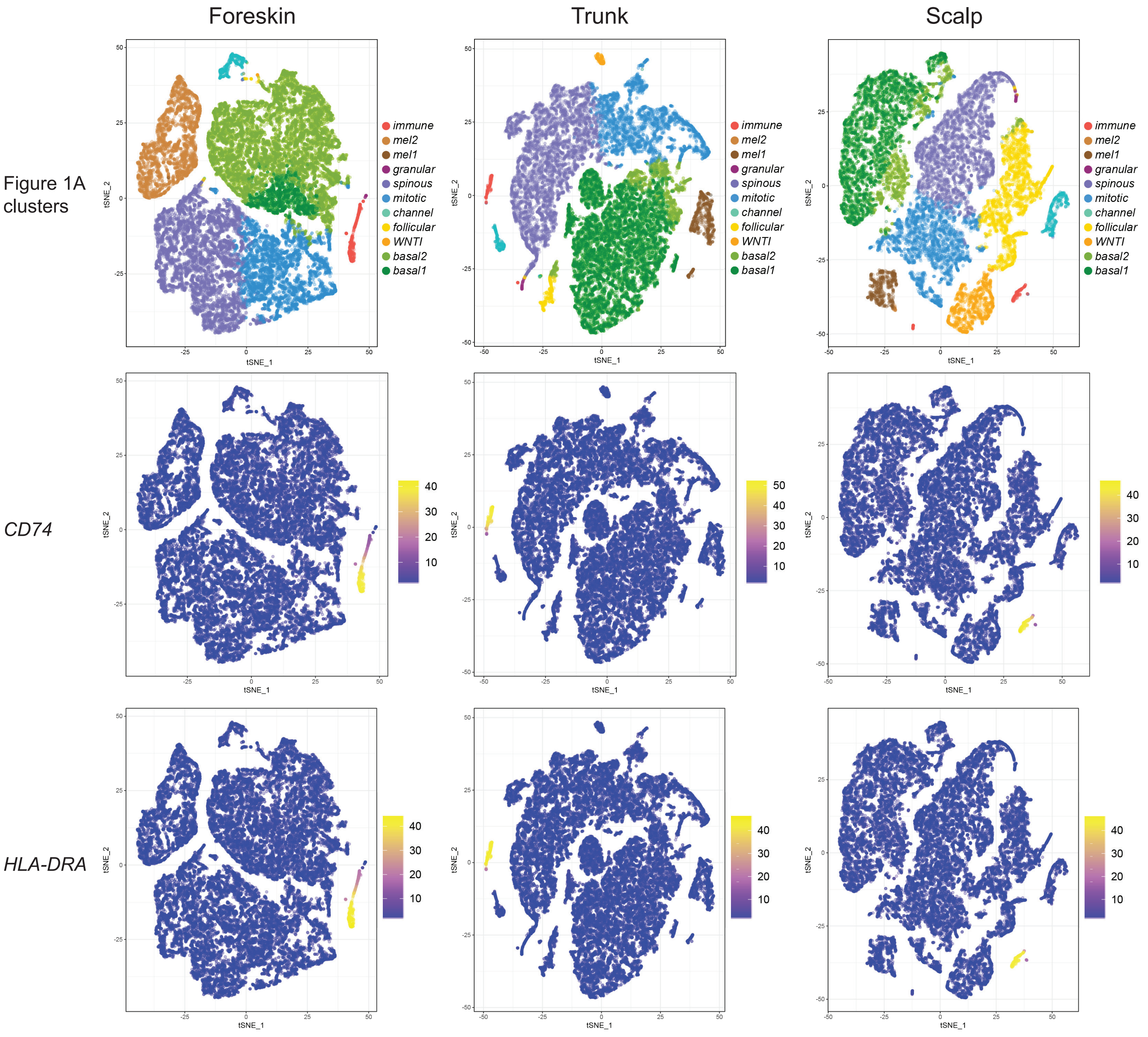

Supplement: 1 [file NIHMS1516330-supplement-1.pdf]
