# Supplementary material for: Transcriptional Programming of Normal and Inflamed Human Epidermis at Single-Cell Resolution
Source: Cell Rep. Author manuscript; Available in PMC 2019 Feb 7. (PMC6367716; doi:10.1016/j.celrep.2018.09.006)

Cell Reports

Article

Transcriptional Programming of Normal and Inflamed Human Epidermis at Single-Cell Resolution

Graphical Abstract

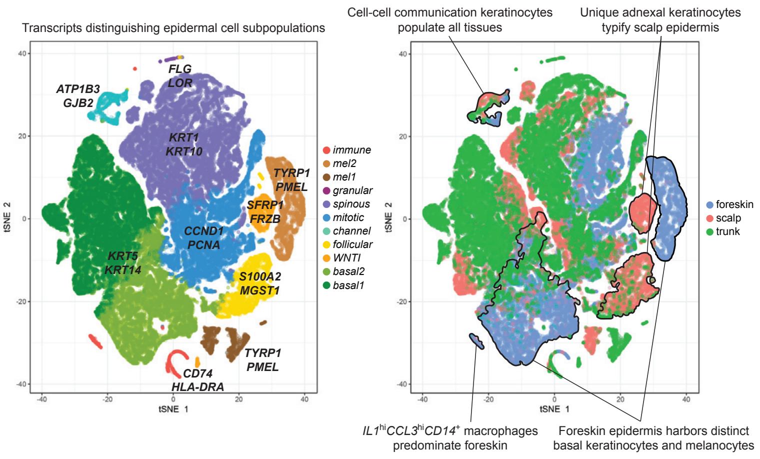

Highlights

- Stereotyped keratinocyte subpopulations modularly comprise human epidermis
- Scalp keratinocytes exhibit an inherent inflammatory transcriptional program
- Myeloid dendritic cells predominate APCs in psoriatic epidermis
- Macrophages represent major APCs in foreskin epidermis

Authors

Jeffrey B. Cheng, Andrew J. Sedgewick, Alex I. Finnegan, ..., Stephen C. Benz, Jun S. Song, Raymond J. Cho

Correspondence

raymond.cho@ucsf.edu

In Brief

Cheng et al. report single-cell RNA sequencing of normal and inflamed human epidermis, revealing a discrete set of specialized keratinocytes that exhibit a distinct composition at different anatomic sites. Myeloid dendritic cells and macrophages also vary sharply with epidermal anatomic site and inflammation, indicating dynamic programming of antigen-presenting cells.

Cheng et al., 2018, Cell Reports *25*, 871–883
October 23, 2018 © 2018 The Authors.
https://doi.org/10.1016/j.celrep.2018.09.006

CellPress

OPEN ACCESS
CellPress

Transcriptional Programming of Normal and Inflamed Human Epidermis at Single-Cell Resolution

Jeffrey B. Cheng,[1,12] Andrew J. Sedgewick,[2,12] Alex I. Finnegan,[3,12] Paymann Harirchian,[1] Jerry Lee,[1] Sunjong Kwon,[4] Marlys S. Fassett,[5] Justin Golovato,[2] Matthew Gray,[2] Ruby Ghadially,[1] Wilson Liao,[5] Bethany E. Perez White,[6] Theodora M. Mauro,[1] Thaddeus Mully,[7] Esther A. Kim,[8] Hani Sbitany,[8] Isaac M. Neuhaus,[5] Roy C. Grekin,[5] Siegrid S. Yu,[5] Joe W. Gray,[4] Elizabeth Purdom,[9] Ralf Paus,[10,11] Charles J. Vaske,[2] Stephen C. Benz,[2] Jun S. Song,[3,13] and Raymond J. Cho[5,13,14,*]

[1]Department of Dermatology, University of California, San Francisco and Veterans Affairs Medical Center, San Francisco, CA, USA
[2]Nantomics, LLC, Culver City, CA, USA
[3]Department of Physics, Carl R. Woese Institute of Genomic Biology, University of Illinois at Urbana-Champaign, Champaign, IL, USA
[4]Department of Biomedical Engineering, OHSU Center for Spatial Systems Biomedicine, Portland, OR, USA
[5]Department of Dermatology, University of California, San Francisco, San Francisco, CA, USA
[6]Department of Dermatology and Skin Tissue Engineering Core, Northwestern University, Chicago, IL, USA
[7]Department of Pathology, University of California, San Francisco, San Francisco, CA, USA
[8]Department of Plastic Surgery, University of California, San Francisco, San Francisco, CA, USA
[9]Department of Statistics, University of California, Berkeley, Berkeley, CA, USA
[10]Centre for Dermatology Research, University of Manchester, Manchester Academic Health Science Centre and NIHR Manchester Biomedical Research Centre, Manchester, UK
[11]Department of Dermatology and Cutaneous Surgery, University of Miami Miller School of Medicine, Miami, FL, USA
[12]These authors contributed equally
[13]Senior author
[14]Lead Contact
*Correspondence: raymond.cho@ucsf.edu
https://doi.org/10.1016/j.celrep.2018.09.006

SUMMARY

Perturbations in the transcriptional programs specifying epidermal differentiation cause diverse skin pathologies ranging from impaired barrier function to inflammatory skin disease. However, the global scope and organization of this complex cellular program remain undefined. Here we report single-cell RNA sequencing profiles of 92,889 human epidermal cells from 9 normal and 3 inflamed skin samples. Transcriptomics-derived keratinocyte subpopulations reflect classic epidermal strata but also sharply compartmentalize epithelial functions such as cell-cell communication, inflammation, and *WNT* pathway modulation. In keratinocytes, ~12% of assessed transcript expression varies in coordinate patterns, revealing undescribed gene expression programs governing epidermal homeostasis. We also identify molecular fingerprints of inflammatory skin states, including *S100* activation in the interfollicular epidermis of normal scalp, enrichment of a *CD1C*$^{+}$*CD301A*$^{+}$ myeloid dendritic cell population in psoriatic epidermis, and *IL1β*^{hi} *CCL3*^{hi}*CD14*$^{+}$ monocyte-derived macrophages enriched in foreskin. This compendium of RNA profiles provides a critical step toward elucidating epidermal diseases of development, differentiation, and inflammation.

INTRODUCTION

Epidermal cells of the skin functionally specialize by altering transcriptional identity. Keratinocytes differentiate from a single lineage to form proliferative basal cells, terminally differentiating cells, a cornified barrier layer, and complex appendages such as hair follicles and sebaceous glands (Goldsmith et al., 2012). Keratinocytes also vary dramatically between anatomic sites, generating protective, hyperkeratotic surfaces on volar surfaces but also thin, permeable mucosae. This remarkable functional diversity reflects a heterogeneous and plastic cell identity dependent on transcription of thousands of genes. However, historically, keratinocytes have been classified based only on morphology and selected molecular markers.

Few single-cell expression studies have previously examined the mammalian epidermis. Published data suggest that the transcript abundances of many genes fluctuate during differentiation and murine hair follicle stem cells and transit-amplifying cells possess distinct transcriptional identities (Joost et al., 2016; Yang et al., 2017). The most comprehensive single-cell transcriptional study of the epidermis utilized microfluidics (i.e., the Fluidigm C1 system) to generate a 1,422-cell composite epidermal sample derived from 19 mice (Joost et al., 2016). Although informative, aggregating independent samplings introduces epigenetic and biological variation, reducing the power to distinguish related groups of cells.

We hypothesized that more completely parsing the molecular heterogeneity of a tissue could advance translational science in two important ways. First, it might identify cell populations enriched in specific diseases, making it possible to assess their

This is an open access article under the CC BY license (http://creativecommons.org/licenses/by/4.0/).

OPEN ACCESS
CellPress

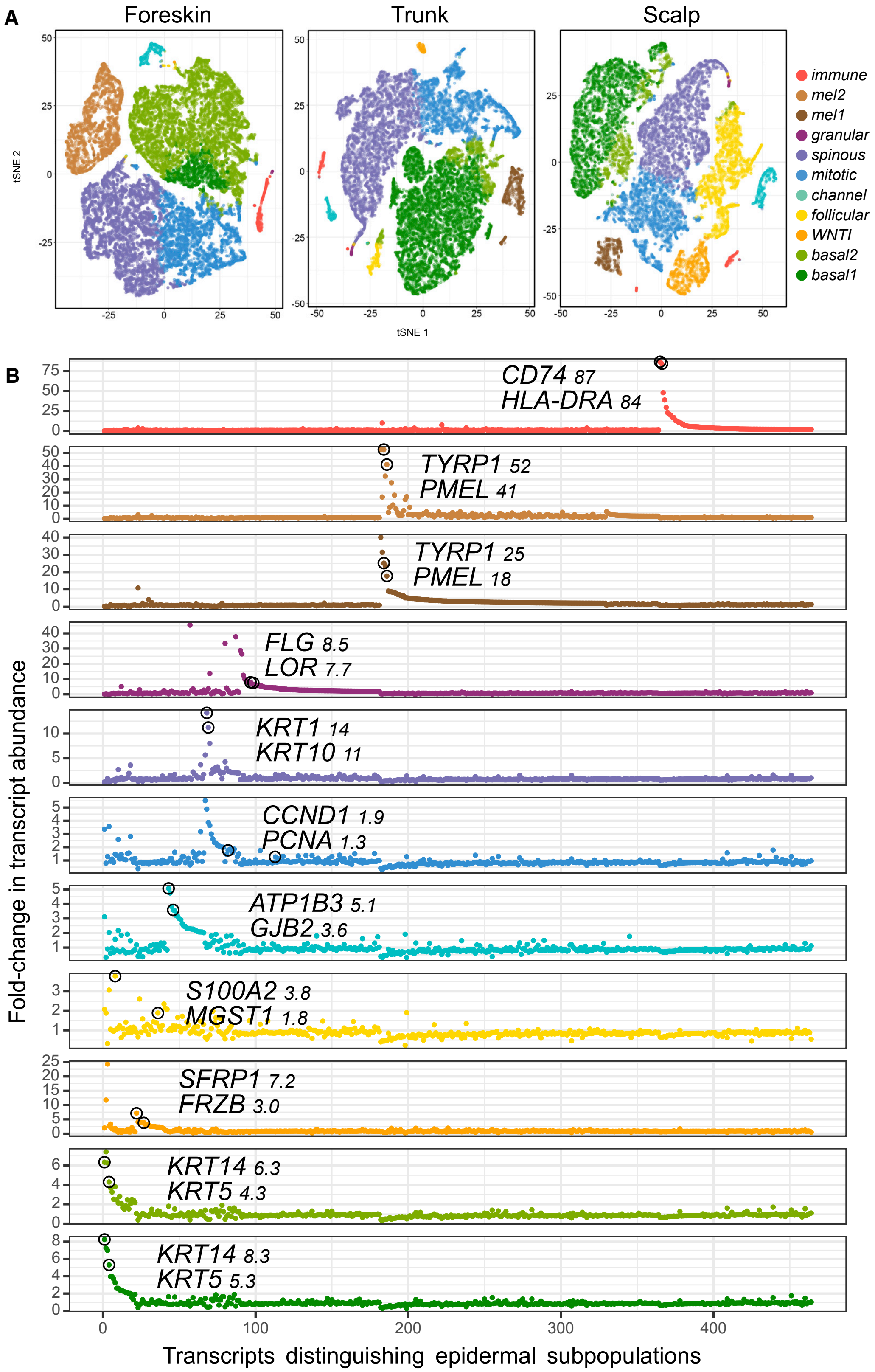

(legend on next page)

pathogenic role and nominate them as therapeutic targets. Second, cataloging disease-related variance between individuals or anatomic sites should help classify pathologic states and personalize treatment.

To finely stratify single-cell subpopulations (e.g., hair follicle or immune cells), large numbers of samples must be studied at each anatomic site. We sought here to initially define normal epidermal molecular heterogeneity, laying the foundation required to place future, higher-resolution studies into context. We present an analysis of single-cell RNA sequencing (scRNA-seq) of three human epidermal samples each from adult scalp, adult truncal skin, and neonatal foreskin (a total of 9 samples). We also compared this dataset with that obtained from three additional samples of psoriatic truncal epidermis. We found that the expression of ~12% of assayed transcripts is regulated in stereotyped patterns during epidermal differentiation, most without a known mechanism. Discrete subpopulations of keratinocytes compartmentalize crucial molecular activities, revealing new functional lineages in the human epidermis.

RESULTS

To interrogate single-cell gene expression at each of these three anatomic sites, normal surgical tissue discards from circumcisions, reduction abdominoplasties and mammoplasties, and scalp excisions were obtained. Three samples of truncal psoriatic skin, obtained from patients not receiving topical or systemic treatment, received a confirmatory histopathologic evaluation from a board-certified dermatopathologist. After sample collection, the epidermis was enzymatically separated from the dermis and dissociated to single cells. Dissociated cell suspensions underwent fluorescence-activated cell sorting to exclude dead cells and cellular debris. Chromium Single Cell 3′ v2 libraries were then generated, followed by next-generation sequencing (Illumina; Experimental Procedures).

Sequencing data were analyzed by treating reads from a single droplet as arising from a single cell, using an identifying bar code (Zheng et al., 2017). All reads were thus organized on a per-transcript and per-cell basis. Factors that can reduce the quality of this approach include cell lysis, which raises non-specific background noise, and incorporation of multiple cells per droplet, resulting in profiling of cell doublets. We first performed fluorescence-activated cell sorting (FACS) to exclude dead cells. To minimize doublets, we loaded limiting numbers of cells for each reaction. For this study, we profiled approximately 2,000–12,000 cells per sample with a range of approximately 40,000–115,000 reads per cell (Table S1). On average, 2,334 genes per cell were detected, similar to previous Chromium single cell RNA-seq studies (Skelly et al., 2018; Tsang et al., 2017). A freely available, browsable collection of gene expressions for these datasets is available at http://scarab-research.nantomics.com/.

Spectral Clustering of Epidermal Single Cells Robustly Identifies Distinct Cell States

By enzymatically dissociating intact epidermis, we expected to generate a heterogeneous mixture of keratinocytes, melanocytes, Langerhans cells, and hematopoietic cells. We discriminated these cell populations by applying spectral clustering (Yan et al., 2009) to the normalized, imputed transcription data (Experimental Procedures; Figure S1). Single cells from multiple tissue samples can be classified in different ways. For example, all cell profiles from multiple specimens can be segregated in a single analysis, favoring the classification of similar cell types shared across samples (Joost et al., 2016). Alternatively, each specimen can be analyzed independently, enabling high-resolution discovery of cell subpopulations specific to that sample.

We employed the former approach to detect recurrent patterns across our independent samples. To avoid conflating related but distinct groups, we partitioned epidermal cells into different numbers of clusters, with n ranging from 8 to 12. For the remainder of our analyses, we utilized 11 clusters, where many established, important cell populations are clearly distinguished (t-distributed stochastic neighbor embedding [t-SNE] representations of these data are shown in Figure 1A). Pseudocoloration of these plots by individual sample shows similar representation in each cluster per individual sample (Data S1A–S1C). A single t-SNE plot incorporating all 9 normal samples reproduces the 11 main clusters (Data S1F). For each cluster, genes with high relative expression distinguishing that subpopulation of cells (compared with all other clusters) are depicted in Figure 1B, projected onto t-SNE plots (Data S1G), and cataloged in Table S2. We also tested *k*-means clustering to partition cell profiles. The resulting subpopulations showed close similarity to those from spectral clustering, as judged by differential expression analysis (Table S3), reflecting the robustness of our approach.

Keratinocyte Subpopulations Demarcate WNT Inhibition and Cell-Cell Communication

We named the two cell populations expressing the highest levels of *KRT5* and *KRT14* (Table S2) '*basal1*' and '*basal2*,' respectively, in accordance with their predicted position in the epidermis. The highest *KRT1*- and *KRT10*-expressing cells also displayed high *DSG1* and *DSP* levels and were termed '*spinous*.' A cluster of cells ('*granular*') expressing a suite of late differentiation markers, including *LOR*, *FLG*, and *SPINK5*, was also identified, although at consistently low cell numbers. Because keratinocyte differentiation is a terminal process that

Figure 1. Unbiased Classification of Epidermal Cell Subpopulations in Human Skin

(A) t-SNE maps show the relatedness of epidermal cell groups distinguished by spectral clustering from three aggregated samples each from foreskin (26,174 cells), trunk (25,129 cells), and scalp (20,561 cells) (see also Figures S1, S4, and S5 and Data S1). Cells in eight of the 11 clusters express high levels of keratins, identifying them as keratinocytes. Two groups representing melanocytes are coded in brown, and the initial immune cell cluster is depicted in red.

(B) Genes with log fold change > 1 in at least 1 cluster ordered by cluster (x axis) and fold change (y axis) (Tables S1, S2, S3, and S6). Genes only appear once, in the first cluster (from bottom to top), where their log fold change passes the threshold. Two genes with specificity for each cluster are named, and their respective coordinates are highlighted with black circles.

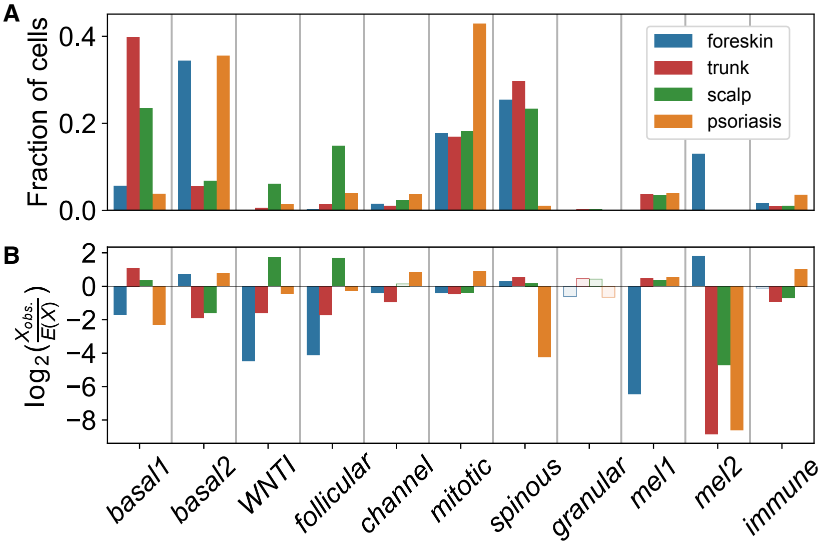

Figure 2. Enrichment of WNTI and Follicular Clusters in Scalp Epidermis

(A) Fraction of cells from each anatomic site or psoriatic skin belonging to each cluster.

(B) Log ratio of the observed number of cells from an anatomic site or psoriatic skin in the cluster to the expected number when sampling cells in cluster uniformly without replacement. Positive and negative log ratios indicate cluster enrichment and depletion for anatomic site or psoriatic skin. All tissue and cluster associations with solid fill bars are significant (p_{adj} < 0.05, Pearson's chi-square test with Bonferroni adjustment).

culminates with nuclear and organelle loss and cell death (Eckhart et al., 2013), we deduced that more differentiated keratinocytes died during preparation or were excluded by DAPI-negative gating during FACS.

The four remaining keratinocyte clusters all showed intermediate levels of *KRT5* and *KRT14*. One showed coordinate elevation of more than a dozen well-recognized DNA synthesis and cell division transcripts, such as *PCNA* and *KI67*. We therefore termed this cluster '*mitotic*.' Another showed high levels of transcripts whose gene products are secreted and antagonize WNT signaling, including *SFRP1*, *FRZB*, *DKK3* (Cruciat and Niehrs, 2013), and *WIF1* (Malinauskas et al., 2011). The third was elevated for transcripts known to be expressed in human follicular root sheaths (*S100A2*; Mitoma et al., 2014), sebaceous gland and root sheath hair follicles (mouse, *APOE*; Grehan et al., 2001; human, *KRT17*; Troyanovsky et al., 1989), and mouse sebaceous glands (*MGST1*; Joost et al., 2016; *APOC1*; Jong et al., 1998). Finally, a fourth cluster was distinguished by coordinate upregulation of ion channel and cell-cell communication transcripts, including *GJB2*, *GJB6*, *ATP1B3*, *ATP1A1*, *ATP1B1*, *ATP5B*, and *FXYD3*, and also mitochondrial channel proteins such as *VDAC2* and *SLC25A5*. These latter three cell populations were named, respectively, '*WNTI*,' '*follicular*,' and '*channel*.'

Two clusters ('*mel1*' and '*mel2*') showed markedly higher expression of the *PMEL*, *TYRP1*, and *MLANA* components of the melanocyte pigment synthesis pathway (Hoashi et al., 2005), identifying them as melanocytes. The final cluster was characterized by the high *HLA* levels associated with immune cells.

We next asked whether our newly identified keratinocyte subpopulations reflect the gross phenotypic variation in epidermis from different anatomic sites. Large disparities in anatomic distribution were immediately apparent (Figures 1 and 2). The *WNTI* and *follicular* subpopulations were significantly enriched in scalp tissue ($p_{adj} < 10^{-309}$, Pearson's chi-square test with Bonferroni correction), more sparse in trunk tissue, and almost absent in foreskin tissue, suggesting that they represent components of hair follicles. In other cases, subpopulations appeared to represent distinct versions of a single cell type in different tissues. For example, the *basal1* and *mel1* subpopulations appear to represent the main basal keratinocytes and melanocytes in scalp and trunk cells. In contrast, *basal2* and *mel2* cells predominate in foreskin.

Temporal Tracing Reveals the Keratinocyte Differentiation Program at Single-Cell Resolution

Keratinocytes undergo a scripted transcriptional program as they travel from a basal, proliferative layer to terminal corneocytes, with ~12% of transcripts differentially expressed between keratinocyte subpopulations (Table S1). We evaluated our eight keratinocyte clusters from normal skin in the context of this progression. We first placed each scalp keratinocyte on a linear spectrum of differentiation based on the expression patterns of established markers: *KRT5*, *KRT14*, *KRT1*, *KRT10*, *IVL*, and *FLG* (Supplemental Experimental Procedures, Pseudotime). As expected, this trajectory partially recapitulated the spectral clustering of keratinocytes, easily visualized by color-coding cells (Figure 3A).

We next used this linear order of cells to inspect both novel and previously undescribed groups of transcripts showing differentiation-related expression. *KRT14* and *COL17A1* show basal-specific expression, reflective of their function at the basement membrane. However, we also discovered a broad array of genes that show closely related patterns of expression; for example, *WNT10A*, *PDLIM1*, *NRG4*, and *RAPGEF1* (Figure 3A). This sort of gene discovery was readily reproduced for other stereotyped expression patterns. The superficial desmoglein *DSG1* predictably shows maximal expression in the granular cluster. However, similar kinetics were seen not only for other cell membrane components (e.g., galectin *LGALS7B*), but also cytoskeletal and cell polarity regulators such as *OSBPL2* (Kentala et al., 2018), *CRB3* (Tapia et al., 2017), and *ESRP1* and *ESRP2* (Warzecha et al., 2010). Notably, genes helping to distinguish the *mitotic*, *follicular*, and *channel* cell clusters did not show linear covariance, indicating that a classic differentiation model of the epidermis fails to distinguish some subpopulations. These data thus highlight the importance of single-cell analysis in discerning cell identities within a heterogeneous population.

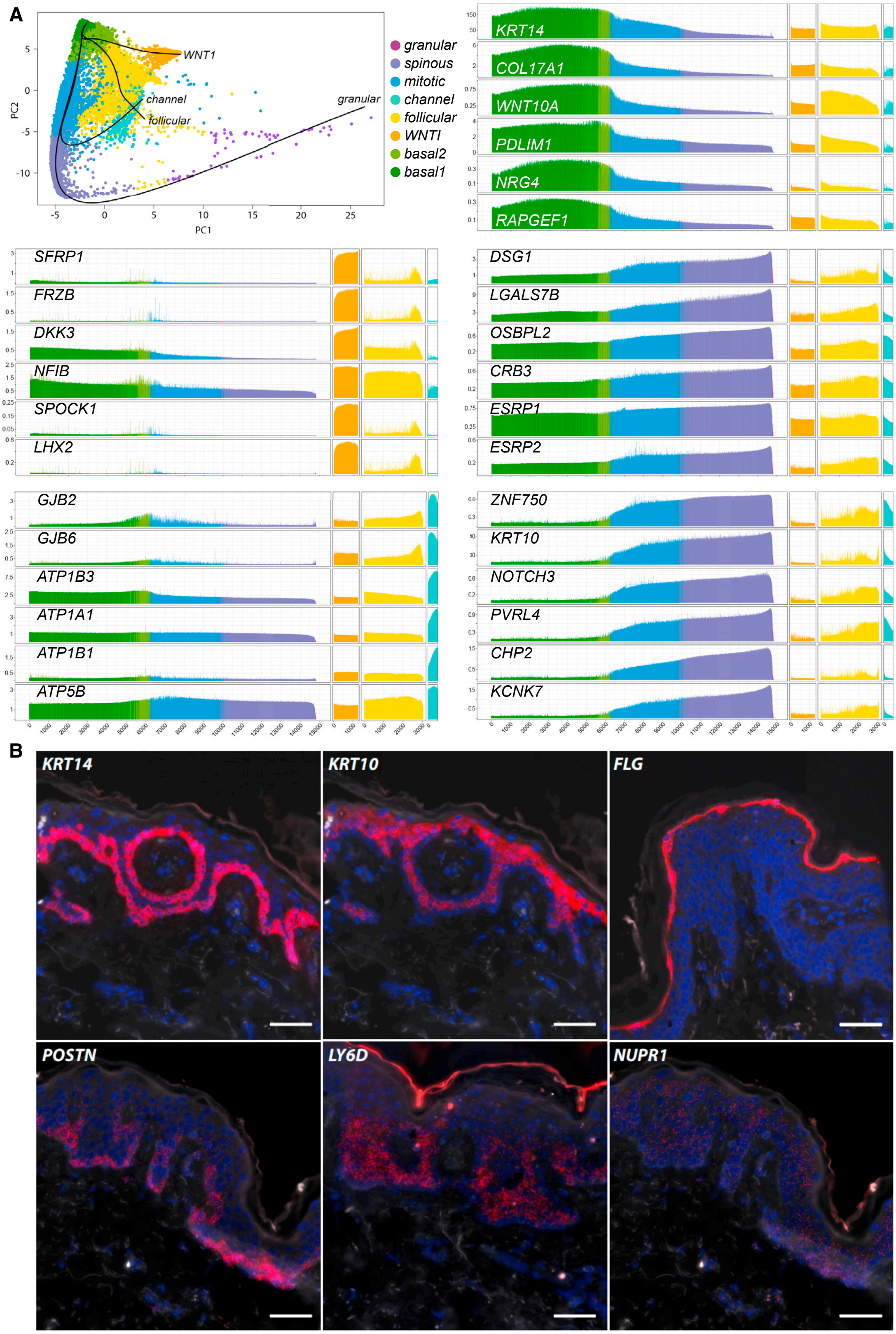

(legend on next page)

We sought to understand the positional specificity of expression patterns in our data. We performed RNA *in situ* hybridization (Kwon et al., 2017) of cluster-specific transcripts alongside genes known to vary with differentiation (*KRT14*, *KRT10*, and *FLG*), as shown in Figure 3B. These data biologically validate our assignments of basal layer expression for *POSTN*, spinous layer expression for *LY6D*, and spinous and granular layer for *NUPR1*. For validation of marker genes for clusters that did not show linear covariance in pseudotime, we performed *in situ* hybridization for *ATP1A1* (which showed a punctate basal and suprabasal pattern that may be representative of the channel cluster) and *KI67* (which displayed a basal and suprabasal pattern characteristic of the *mitotic* cluster; Figure S2). Additionally, we plotted transcript expression of the *mitotic* cluster cell cycle genes (*PCNA*, *CENPF*, *KI67*, and *CCNA2*) against *KRT10* abundance to show that their expression peaks at an intermediate *KRT10* level (Figure S3).

Amphiregulin Enrichment Distinguishes a Subpopulation of Foreskin Basal Keratinocytes

Coarse clustering of cells from heterogenous tissues, as in our initial approach, may lack the discriminative power to identify finer subpopulations of cell types. To search for such classes, we re-analyzed the largest cluster in our original analysis (basal keratinocytes comprising *basal1* and *basal2*) in all 9 normal samples, performing spectral clustering at n = 3–10 subdivisions. To avoid finer subdivisions arising from batch artifacts (Figures S4A–S4C), we required that at least 5% of cells in each new cluster derive from each of the three samples of a contributing tissue type.

We focused on n = 3, where all samples from a contributing tissue were robustly represented in each cluster (Figures S4D–S4F). This analysis reproduces the trunk and scalp (*basal1*) and foreskin and psoriasis (*basal2*) dominant subpopulations from the original analysis. In addition, we also identify a new, clearly demarcated cluster specific to foreskin ('*basal3*'). Limma-based differential gene expression analysis (Ritchie et al., 2015) between these three clusters shows that *basal1* is particularly enriched for *CXCL14* and *DMKN*, whereas *basal2* is enriched for *CCL2* and *IL1R2*, suggesting immunosecretory distinctions among basal keratinocytes. In contrast, *basal3* is highly enriched for amphiregulin (*AREG*), an epidermal growth factor receptor (EGFR) ligand that promotes keratinocyte proliferation (Stoll et al., 2016; Table S2).

Scalp Keratinocyte Transcriptomes Identify *MGST1* and *TKT1* as Human Follicle Markers and Localize *S100* Overexpression to Interfollicular Epidermis

Our successful secondary partitioning of basal epidermis suggested that we also focus this approach on scalp keratinocytes to identify the specialized cell subpopulations characteristic of hair follicles. We re-clustered the keratinocyte populations in our three scalp samples at n = 10–20. At 15 clusters, putative subpopulations in human hair follicles resolved further without duplicating subcategories (Figure 4; Table S4). WNT-inhibitory transcripts found in the initial multi-anatomic site *WNTI* cluster (*SFRP1*, *FRZB*, and *DKK3*) again localized to a discrete cellular population in this scalp-specific analysis (named *high-resolution WNTI*, or '*HR-WNTI*'). These cells may represent outer bulge cells, which, in mice, have been shown to secrete WNT inhibitors, influencing differentiation of the inner bulge (Lim et al., 2016). The elevated *MGST1* and *APOE* transcripts of the *follicular* cluster were also identified in a scalp subpopulation we named '*sebaceous*'. Our higher-resolution clustering additionally identified '*UHF diff*,' a cluster potentially analogous to mouse differentiated upper hair follicles (*CST6* [Veniaminova et al., 2013]; *KRT17* [Joost et al., 2016]; *KRT79* [Joost et al., 2016]).

To validate the spatial specificity of markers from these clusters, we used *in situ* staining to localize RNA for *MGST1*, *TKT*, and *SFRP1* (Figure S2). *MGST1* (previously reported as a mouse sebaceous gland marker; Joost et al., 2016) and *TKT* are prominently expressed in the sebaceous gland epithelium, with *TKT* expression most pronounced at the periphery. *SFRP1* shows strong expression in the cuboidal cells of the outer root sheath at the base of the hair follicle.

Distinct interfollicular epidermis (IFE) clusters identified in the multisite analysis also appeared in our scalp analysis, including analogs of the *basal*, *spinous*, *mitotic*, and *channel* subpopulations. Notably, elevated *S100A7*, *S1008*, and *S100A9* expression was restricted to a distinct IFE cluster, a class not resolved in the lower-resolution multi-site analysis.

Epidermal Subpopulations Shift Immunological and Proliferative Programs between Anatomic Sites

When a cell subpopulation is found at multiple anatomic sites, transcriptional differences at the different sites may arise within this classification, revealing distinct functional specialization. We therefore directly compared our original 11 aggregate normal tissue epidermal subpopulations between anatomic sites, utilizing differential gene expression analysis (Figure 5; Experimental Procedures; Tables S5 and S6). The most dramatic disparity was detected in scalp, where inflammatory genes were enriched in the set of all transcripts showing scalp-specific upregulation (Fisher's exact test, $p = 2.1 \times 10^{-3}$; Experimental Procedures). Specifically, inflammation-related transcripts of the *S100* family (*S100A7*, *S100A8*, and *S100A9*) as well as *IFI27* were generally elevated compared with other tissues. Sub-clustering of scalp

Figure 3. Coordinate, Finely Distinguished Kinetics of Gene Expression in Differentiating Scalp Keratinocytes
(A) Top left: the longest pseudotime reconstruction of differentiation (line ending in purple granular cells) defines basic keratinocyte differentiation used in the other panels. Other pseudotime lines show distinct differentiation pathways from basal cells to WNTI, follicular, and channel cells. In the remaining five panels, the leftmost section shows transcript abundance (in imputed counts/10,000, y axis) in about 21,000 pseudotime-ordered differentiating scalp keratinocytes on the x axis, from left to right. Also charted are transcript levels in WNTI, follicular, and channel cells in the remaining 3 sections. Left center and left bottom: genes distinguishing the WNTI and channel clusters, respectively. Right: distinct kinetics of differentiation-dependent transcript regulation.
(B) RNA *in situ* hybridization staining (red channel) confirms the layer specificity of genes identified in this report: basal layer *POSTN*, spinous layer *LY6D*, and spinous and granular layer *NUPR1*. The blue channel represents DAPI staining. Scale bars, 50 μm.
See also Figure S2.

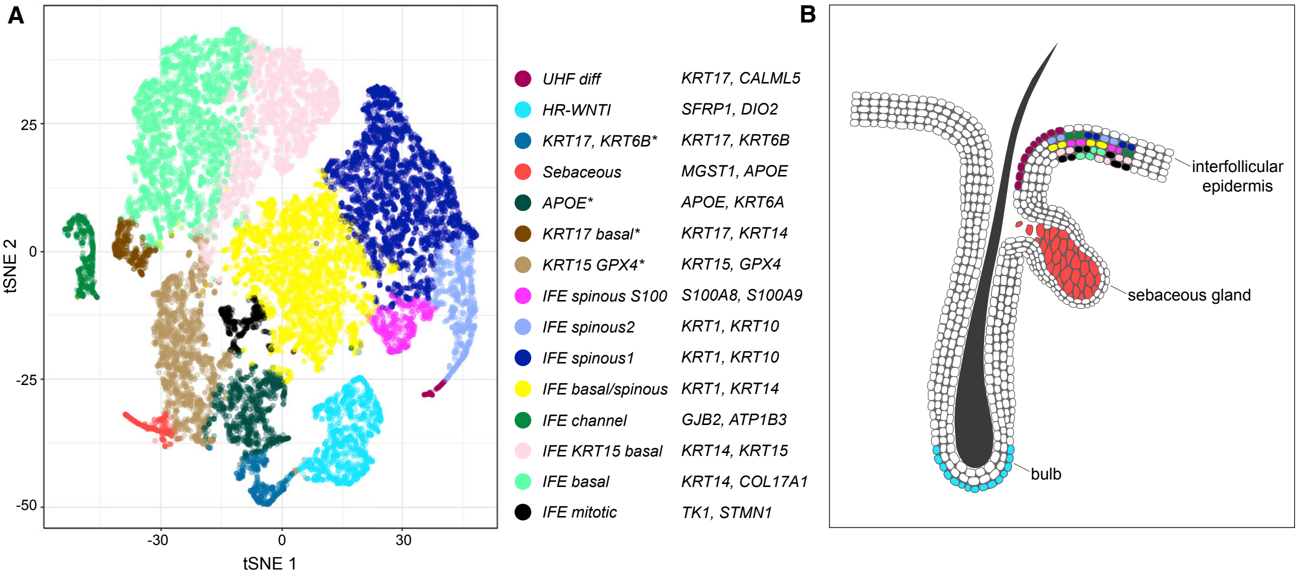

Figure 4. Distinctive Epidermal Cell Subpopulations in Human Scalp Corresponding to Follicular and Interfollicular Keratinocytes
The t-SNE map shows spectral clustering of scalp keratinocytes into 15 groups (Table S4), which reveal correlates of outer and inner bulge cells, sebaceous gland cells, upper follicular epithelium, and also recapitulation of multi-site interfollicular epithelium strata. *These cell clusters could not be confidently assigned locations and are not depicted in the follicle diagram. See also Figure S2 and Data S1.

cells identified an IFE subpopulation particularly enriched for these transcripts (Table S4).

Aside from the immunosecretory and proliferative differences in basal keratinocytes discussed above, truncal skin was elevated for *CXCL14*, *CCL27*, and *NFKBIA*, suggesting that the innate immune repertoire of keratinocytes may more generally vary with anatomic site.

We also performed gene ontology (GO) analysis with the Database for Annotation, Visualization and Integrated Discovery (DAVID) (Huang et al., 2009a, 2009b) to assess whether keratinocytes in aggregate from different anatomic sites showed functional enrichment. Foreskin keratinocytes were enriched for cell division, mitotic nuclear division, and RNA splicing and processing terms, consistent with the known greater proliferative capacity of neonatal keratinocytes (Table S7; Gilchrest, 1983).

Psoriatic Epidermis Is Enriched for *Mitotic* and *Channel* Keratinocytes and *CD1C*$^+$ *CD301A*$^+$ Myeloid Dendritic Cells

We assessed how inflamed epidermis is transcriptionally altered on a single-cell level. Psoriatic keratinocytes were enriched for the *mitotic* and *channel* subpopulations, showing the plasticity of cell transcriptional identities in disease states (Figure 2; $p_{adj} < 10^{-309}$ and $p_{ad} = 1.4 \times 10^{-74}$, respectively; Pearson's chi-square test with Bonferroni correction). Transcripts of *S100* genes were generally elevated in psoriatic epidermis, most markedly in the superficial *spinous* and *granular* cell clusters (Figure 5D). Notably, *S100* transcripts were also elevated in melanocytes and immune cells of psoriatic skin, revealing a multi-lineage response to epidermal inflammation.

We further partitioned the immune cell epidermal subpopulation from our normal and psoriatic samples into 5 clusters (Supplemental Experimental Procedures) and identified CD3$^+$ $\alpha\beta$ T cells as well as three clusters representing major histocompatibility complex (MHC) class II$^+$ antigen-presenting cells of the myeloid lineage: *CD207*$^+$*CD1A*$^+$ Langerhans cells, *CD1C*$^+$*CD301A*$^+$ myeloid dendritic cells (DCs), and *CD14*$^+$*CCL3*^{hi}*IL1β*^{hi} monocyte-derived macrophages (Figure 6). We did not identify distinct hematopoietic clusters representing other lymphoid or myeloid subsets— e.g., $\gamma\delta$ T cells or neutrophils.

Mapping our 12 samples onto these 5 immune cell clusters revealed biases related to anatomic site and inflammatory state. *CD1C*$^+$ *CD301A*$^+$ myeloid DCs were enriched in psoriatic epidermis, present in scalp skin and foreskin, and sparse in truncal epidermis (Table S8; Figure 7). In contrast, *CD14*$^+$ *CCL3*^{hi}*IL1β*^{hi} macrophages were generally restricted to foreskin. *CD207*$^+$*CD1A*$^+$ Langerhans cells were detected across all anatomic sites but underrepresented in psoriatic skin.

There was a trend toward increased CD3$^+$ $\alpha\beta$ T cells in psoriatic trunk and scalp samples. Although the T cell lineage markers *CD4*, *CD8*, and *FOXP3* were not detectable within the CD3$^+$ $\alpha\beta$ T cell cluster, specific genes suggested a heterogeneous population of activated, antigen-experienced T cells (*IL2RG*, *CD69*, and *CD44*), cytotoxic T cells (*GZMA* and *GZMB*), and regulatory T cells (*IL2RG* and *TNFRSF18*). Notably, the only CD4 T helper cell lineage-defining transcription factor identified was *GATA3* (low positive). We did not detect *TBET* or *RORC*.

DISCUSSION

The human epidermis epitomizes the complexity of a multi-lineage tissue. Not only does its function rely on exquisite, stratified programming of keratinocytes spatially co-organized with immune and nerve cells, but the tissue is also epigenetically

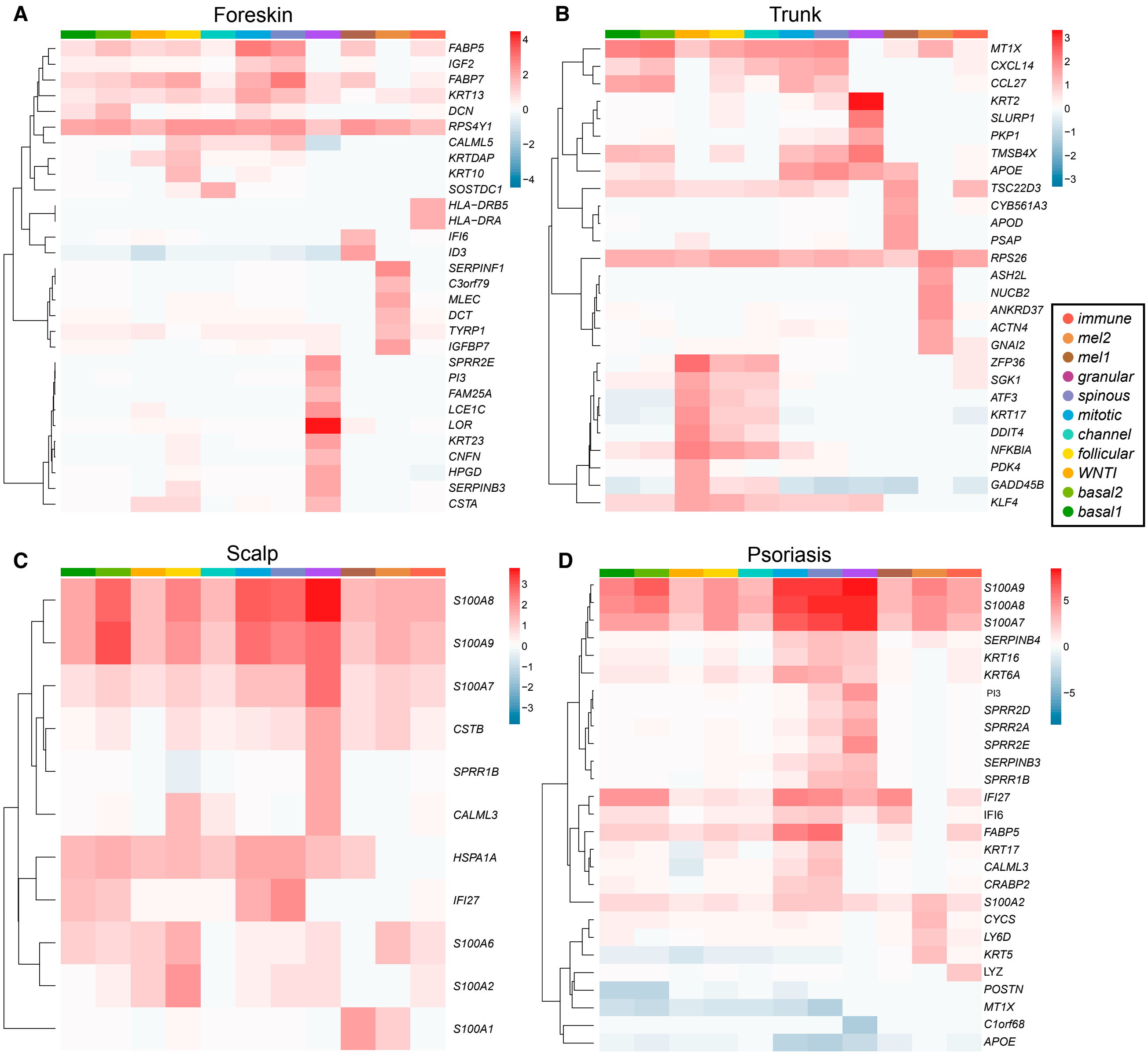

Figure 5. Inflammatory S100 and IFI27 Elevation in Human Scalp and Psoriatic Skin

(A–D) Heatmaps showing fold change of differential gene expression, by cluster, for (A) foreskin, (B) trunk, and (C) scalp, each relative to the other anatomic sites. In (D), psoriatic epidermal subpopulations are compared only with normal trunk. In each cluster, limma-trend was used to calculate the fold change and statistical significance of the difference in the mean expression of each gene for each tissue comparison based on non-imputed UMI counts per million (Table S5). Genes with a $\log_2$ fold change (FC) greater than 1.5 (scalp and foreskin) or 2.5 (psoriasis) and false discovery rate (FDR) < 0.05 in at least one cluster are displayed (Experimental Procedures; Tables S5, S6, and S7). An elevated RPS4Y1 transcript in all foreskin clusters confirms Y chromosome-specific bias for sex.

repurposed in distinct skin locations. Furthermore, the epidermis evolves multi-dimensionally to counter challenges such as infection or wounding. Here we report the first single-cell transcriptional profiling of human epidermis from multiple anatomic sites based on matched transcriptomes of keratinocytes, melanocytes, and immune cells. The data underlying this study, which also includes three samples of psoriatic epidermis, represent approximately 8.4×10^8 unique molecular identifiers of transcript abundance in 92,889 cells, a vast compendium available for analysis in diverse biological contexts, most beyond the scope of this paper. However, these foundational analyses already present fascinating insights into epidermal composition and function.

The algorithmic partitioning of transcriptomes from keratinocytes, the most abundant cell in the epidermis, partially recapitulates known differentiation strata such as the basal epidermis, *stratum spinosum*, and *stratum granulosum*. We were able to further subclassify populations to identify subpopulations such

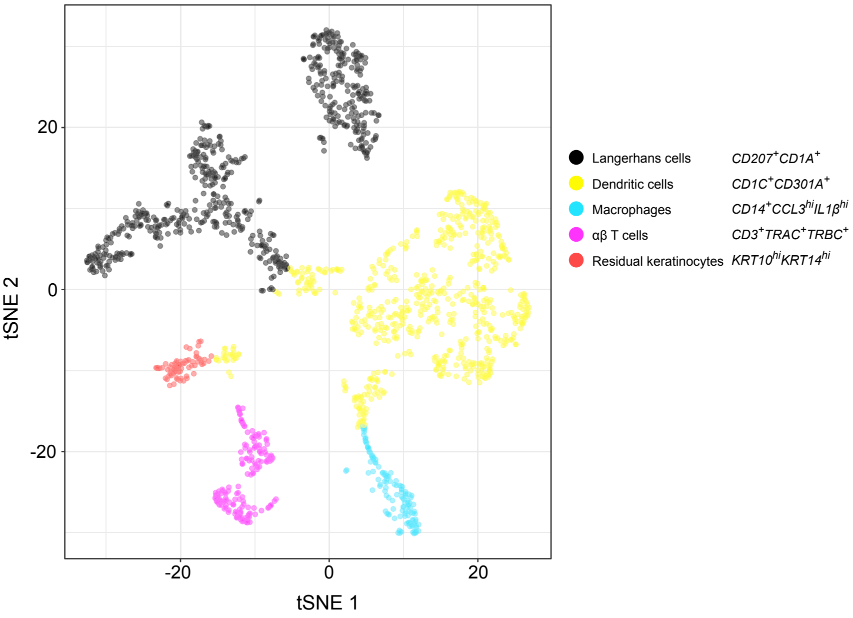

Figure 6. Unbiased Detection of T Cells, Macrophages, and Dendritic Cells in Normal and Inflamed Epidermis

The t-SNE map shows spectrally clustered hematopoietic cell populations from all 12 of our normal and inflamed epidermal samples. The spatial adjacency of Langerhans cells, dendritic cells, and macrophages illustrates their relatedness compared with T cells (purple) and a small number of keratinocytes expressing inflammatory transcripts and, thus, misclassified with immune cells (red). See also Table S8 and Data S1.

as *basal3* in foreskin, whose enrichment for the EGFR ligand *AREG* may help explain the higher proliferative potential of neonatal skin (Gilchrest, 1983). As in the Joost et al. (2016) mouse epidermal scRNA study, a specific progenitor population was not clearly resolved by current partitioning approaches.

However, our analysis also demonstrates the presence of previously undescribed subcategories that are not a linear correlate of stratified differentiation. The *channel*, *follicular*, and *WNTI* classes are identified independently both by spectral and k-means clustering of the transcriptomics data (Tables S2 and S3), suggesting a discrete, reproducible nature. In a previous study (Joost et al., 2016), 25 distinct mouse epidermal subpopulations were reported, including 5 subpopulations from the interfollicular epidermis (Joost et al., 2016). Despite a degree of arbitrariness in clustering, this number of groups is within range of the 15 identified in our high-resolution analysis of human scalp (Figure 4; Table S4). The mouse study established layer specificity for numerous genes that also show such patterning in the human epidermis, including *POSTN*, *LY6D*, and *NUPR1*.

The enrichment of *WNTI* and *follicular* cells in scalp, low levels in trunk, and virtual absence from foreskin suggest that they represent elements of terminal hairs. We examined both gene expression and *in situ* staining to map our finer (15 class) division of scalp keratinocytes to follicular structures. The *HR-WNTI* cluster retains elevated levels of *SFRP1*, *DKK3*, *KRT15*, *WIF1*, *PHLDA1*, *DIO2*, *DPYSL2*, *DCN*, and *DCT*, all previously identified as selectively upregulated in the human hair bulge (Lim et al., 2016; Ohyama et al., 2006). *In situ* localization of *SFRP1* to the outer root sheath of the hair bulb suggests that this bulge-related population suppresses Wnt signaling, perhaps in regressing telogen hair follicles (Geyfman et al., 2015).

The scalp-specific sebaceous population contains elevated levels of *MGST1*, a glutathione S-transferase recently observed in mouse sebaceous glands (Joost et al., 2016). *MGST1* diffusely stains the central portion of sebaceous glands harboring mature cells preceding holocrine secretion. However, the *TKT* transcript is sharply delimited to the peripheral germinative layer, suggesting that transketolase correlates with sebocyte proliferation and renewal (Hinde et al., 2013). Further *in situ* staining for differentially expressed transcripts in our scRNA data may identify additional novel, spatial markers in epidermis.

Intriguingly, clinically normal scalp epidermis consistently shows upregulation of inflammatory *S100* transcripts in the IFE. Uninflamed scalp keratinocytes also express greater *IFI27* levels, particularly in the *granular* cluster, another marker typifying psoriatic epidermis (Suomela et al., 2004) and functionally capable of driving keratinocyte proliferation (Hsieh et al., 2015; Figure 5C). Elevation of *S100A9* has been detected in clinically normal scalp of psoriatic individuals (Ruano et al., 2016). However, this is the first report, to our knowledge, that establishes high levels of these transcripts in normal scalp, suggesting a cause for the inflammation, pruritus, and scale often observed at this site. In foreskin keratinocytes, we instead find upregulation of proliferation-related transcripts. Foreskin also expresses a different suite of inflammatory transcripts, perhaps reflecting distinct immunosurveillance at this site. Because of the challenges in obtaining female neonatal or adult genital tissue, it remains to be determined whether these molecular characteristics are associated primarily with genital or neonatal skin.

The striking induction of pore and intercellular communication transcripts in the putative *channel* cluster describes a potentially novel keratinocyte subpopulation. Its relatively consistent abundance in foreskin, trunk, and scalp samples suggests a universal cell identity. Although *GJB2* is found in both follicular and interfollicular keratinocytes, our *in situ* staining of *ATP1A1*, highly specific and a potential marker for this cluster, displays punctate localization to basal and suprabasal interfollicular epidermis, suggestive of a role as a specialized subpopulation. In the higher-resolution clustering of scalp epidermis, the *channel* subpopulation segregates away from hair follicle markers such as *KRT17*, also supporting its position in interfollicular epidermis. Combinatorial staining of additional *channel* markers should help more precisely localize these cells in normal and diseased epidermis.

Psoriatic epidermis is enriched for *channel* cells (Figure 2B), which show elevated levels of the psoriasis-associated keratins

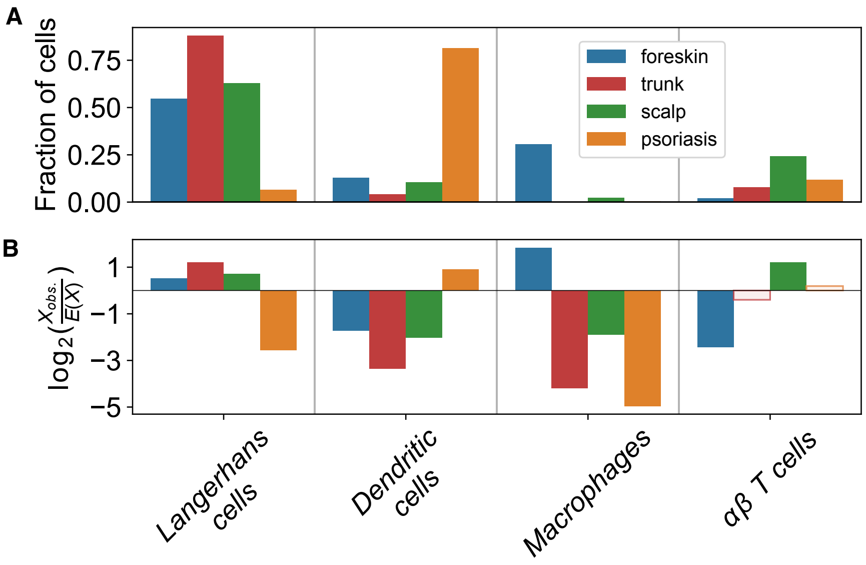

Figure 7. Enriched Antigen-Presenting Cells in Psoriatic Epidermis and Macrophages in Foreskin

(A) A fraction of cells from each anatomic site or psoriatic skin belonging to each immune cluster.

(B) Log ratio of the observed number of cells from anatomic site or psoriatic skin in cluster to the expected number when sampling cells in the cluster uniformly without replacement. Positive and negative log ratios indicate cluster enrichment and depletion for anatomic site or psoriatic skin. All tissue and cluster associations with solid fill bars are significant (p_{adj} < 0.05, Pearson's chi-square test with Bonferroni adjustment). No trunk cells occurred in the macrophage cluster, so a pseudocount of 1 cell was added to allow illustration of the $\log_2$ fold depletion.

KRT6A and *KRT16*. Gene expression of the established psoriasis risk gene *GJB6* (connexin 30.3) is also increased (Sun et al., 2010). Thus, the increased expression of these genes in inflamed skin may be at least partly explained by the expansion of rare *channel* cells in normal skin. Peak expression of *DEFB1* in this cluster, and the known association between *KRT16* and innate immunity (Lessard et al., 2013), support an inherent inflammatory nature of these cells. Germline variants in *GJB6* and *GJB2* (connexin 26), which are similarly enhanced in this subpopulation, cause keratitis-ichthyosis-deafness syndrome, typified by transient inflammatory erythrokeratoderma (Richard et al., 2002). How the expansion of *channel* cells contributes to the behavior of psoriatic or inflamed epidermis remains to be explored.

In addition to enrichment for *channel* cells, psoriatic epidermis is remarkable for expansion of the *mitotic* subfraction, (p_{adj} < 10^{-309}, Pearson's chi-square test with Bonferroni correction), revealing its proliferative nature. Although the dividing fraction of keratinocytes is commonly assigned to the *stratum basale*, charting these cell cycle-related transcripts against *KRT10* expression in our data suggests that they remain active well into suprabasal layers (Figure S3). Our *in situ* staining of *KI67* supports this interpretation (Figure S2). Our single-cell expression data also show enhancement of inflammatory transcripts peaking in the suprabasal layers of psoriatic skin. Some have been identified previously, including *S100A7* (Wolf et al., 2008) and *S100A8* and *S100A9* (Benoit et al., 2006), but some appear novel, as for *IFI27* and *PI3.*

Our immune cell analysis is limited by our focus on the epidermis, excluding the diverse, important subpopulations that reside primarily in the dermis. Furthermore, in this study, we profile immune cells in true proportion to keratinocytes of the epidermis without the CD45 enrichment required for higher-resolution analysis. Therefore, important immune cell populations are represented at very low abundance (for example $CD3^+$ αβ T cells; Figure 6), making it challenging to partition them into their constituent CD4 or CD8 components. However, even at this relatively low resolution, we discern previously undescribed patterns of immune cell activation, primarily in the DCs abundant in the epidermis.

We find psoriatic epidermis to be surprisingly enriched for *CD1C*$^+$*CD301A*$^+$ myeloid DCs relative to normal epidermis from all anatomic sites, with fewer macrophages and Langerhans cells. Although dermal DC populations have been established to expand in psoriatic skin (Zaba et al., 2009), our data suggest that this specific subset of DCs also markedly expands in psoriatic epidermis. Thus, epidermal DC proliferation and/or recruitment may further fuel activation of effector lymphoid and myeloid cells to produce the clinical features of psoriatic skin. We also report that macrophages are highly enriched in foreskin, possibly involved in antigen presentation from neonatal or urogenital pathogens.

Even in our relatively sparse $CD3^+$ αβ T cells, we detect elevated transcripts representative of *GZMA*$^+$*GZMB*$^+$ cytotoxic T cells and *CD44*$^+$ memory cells, not previously considered primary residents of normal skin. Whether this population includes the recently described $CD8^+CD49^-$ resident memory T cells enriched in psoriatic skin is unclear (Cheuk et al., 2017). $CD3^+$ αβ T cells are also notably elevated in normal scalp skin. Skin-resident T cells are known to cluster around terminal hair follicles, with diverse functions including hair follicle cycling and immune response to microbiota (Ali et al., 2017). T cell enrichment in scalp epidermis may simply reflect high hair density in this site. However, the cytotoxic T cell subpopulations suggested by our data may be relevant for the pathogenesis of lymphocyte-mediated alopecias (Bolduc et al., 2016; Xing et al., 2014). Future studies in which hematopoietic lineage cells are enriched after epidermal dissociation should greatly enhance resolution when examining T cell compartments.

The keratinocyte and immune cell transcriptional programs we report in psoriatic epidermis demonstrate how scRNA-seq rapidly provides a multi-dimensional fingerprint of inflammatory disease. This approach may classify rashes whose origins could not previously be determined and match treatments to rashes in specific anatomic regions.

The expansive dataset published here lends itself to many additional analyses. Protein or mRNA expression of some genes

has been associated previously with specific epidermal layers. However, the closely related gene expression patterns in Figure 3A can be exploited to detect new regulatory elements coordinating their activity. For example, suprabasal expression of the keratinocyte differentiation factor *ZNF750* shows kinetics precisely related to epithelial regulators but also poorly characterized genes such as *NOTCH3*, *PVRL4*, *CHP2*, and *KCNK7* (Figure 3A). Sequence-based and functional studies may now elucidate the regulatory mechanisms effecting their parallel control.

EXPERIMENTAL PROCEDURES

Tissue Isolation

For each of the three anatomic sites examined, normal surgical tissue discards from circumcisions, reduction abdominoplasties or mammoplasties, or scalp excisions were obtained. Written informed consent for the samples was obtained using protocols approved by the University of California, San Francisco (UCSF) institutional review board. Fresh tissue was initially placed in medium 154 with human keratinocyte growth supplement and 0.07 mM $CaCl_2$ (Thermo Scientific) and stored at 4°C for 1–2 days prior to single cell isolation. The epidermis was enzymatically dissociated from the dermis with dispase (Corning) incubation for 2 hr at 37°C. Epidermal sheets were then manually separated from the dermis and then dissociated into single cells with trypsin (Thermo Scientific) incubation at 37°C for 15 min. The dissociated cell suspension was strained with a 40-μM filter, washed with medium 154, and then underwent FACS to exclude DAPI-positive dead cells, doublets, and debris. Sorted cells were centrifuged and resuspended in 0.04% BSA (Sigma) in PBS (Thermo Scientific). Chromium Single Cell 3′ v2 (10X Genomics) library preparation was then performed by the Genomics Core Facility, UCSF Institute for Human Genetics, according to the manufacturer's protocol.

Sequencing

Chromium Single Cell 3′ v2 libraries were sequenced with either an Illumina HiSeq 2500, HiSeq 4000, or NovaSeq 6000 following the manufacturer's protocol. For libraries sequenced using Illumina HiSeq 2500 (high output mode) or HiSeq 4000, the following sequencing parameters were used: read 1, 26 cycles; i7 index, 8 cycles; i5 index, 0 cycles; and read 2, 98 cycles. For libraries sequenced using a NovaSeq 6000 S2 reagent kit, the following paired-end sequencing parameters were used: read 1, 26 cycles; i7 index, 8 cycles; i5 index, 0 cycles; and read 2, 91 cycles.

Primary Computational Analysis

Primary computational analysis started from raw Illumina sequencing data and culminated in cell clusters. Raw data were processed using Cellranger (10X Genomics version 2.0.2) and filtered using Seurat (Macosko et al., 2015; Supplemental Experimental Procedures, Data Processing and QC Filtering). We used zero-inflated negative binomial-based wanted variation extraction (ZINB-WaVE) (Risso et al., 2018) to obtain a low dimensional representation of cells, removing variations attributable to library size, mitochondrial read composition, and batch effect. We used the Markov affinity-based graph imputation of cells (MAGIC) imputation algorithm (van Dijk et al., 2017) with cell-cell similarity measured by the ZINB-WaVE low-dimensional representation to mitigate effects of dropout (Supplemental Experimental Procedures, Imputation, Choice of Magic t Parameter). Imputed expression values were used to cluster cells by applying principal component analysis (PCA), followed by k-means-based approximate spectral clustering (Yan et al., 2009; Supplemental Experimental Procedures, Principal Component Analysis, Spectral Clustering). Finally, we used Slingshot (Street et al., 2018) to assign developmental pseudotime to the scalp cells and demonstrated that the clustering results are robust (Supplemental Experimental Procedures, Pseudotime, t-SNE Mapping, Processing Time, and Sex-Specific Bias Analysis).

Differential Expression

We used limma-trend version 3.34.8 (Law et al., 2014) based on the application of this method to scRNA data from Soneson and Robinson (2018). For this analysis, we considered a set of 6,337 genes that had at least 3 unique molecular identifiers (UMIs) in at least 100 cells across all samples. UMI count data are converted to log-scaled counts per million (log-CPM) with an offset of 1. Linear models are fit to the log-CPM profiles of each transcript with membership of each cluster as a binary covariate. To evaluate differential gene expression between clusters, the mean log-CPM of each gene in the cells in each cluster is compared with the mean log-CPM of the gene in all other cells. To evaluate the differential expression of transcripts in each tissue on a per-cluster basis, we fit independent linear models for the cells in each cluster with tissue membership as a binary covariate. For each transcript, the per-cluster mean log-CPM for each healthy tissue was compared with the mean across the other two healthy tissues. The mean log-CPMs in psoriatic cells were compared with the means in truncal cells. Moderated t statistics for the differences in means are calculated using an empirical Bayes approach. False discovery rate is calculated from p values associated with the t statistics to evaluate statistical significance.

GO Analysis

We performed two-part GO analysis on lists of genes with consistent differential expression between foreskin, scalp, and trunk across keratinocyte subpopulations. Lists were constructed by first restricting attention to the set of genes differentially expressed between tissues in any keratinocyte subpopulation (Table S5; $p_{adj} < 0.05$); each gene was then assigned to a tissue, along with its direction of differential expression, when the gene was differentially expressed in that tissue in at least 1 subpopulation and not differentially expressed in the same direction for any other tissue across all subpopulations. A single gene, *HACD1*, was assigned as having tissue-specific expression in trunk tissue in both directions and was excluded from downstream analysis. Table S7 provides the resulting gene lists with direction of differential expression.

Using these gene lists, we first investigated whether the apparent enrichment for inflammatory function in scalp-specific genes, depicted in Figure 5, was significant when extended to all genes upregulated in scalp keratinocytes. Significance was assessed with Fisher's exact test applied to the intersection of this gene list with a set of genes annotated for inflammatory response in Uniprot or as positive regulators of inflammatory response in AmiGO. The background gene set consisted of all genes with raw expression measurements.

Second, we used DAVID (Huang et al., 2009a, 2009b) to search more broadly for functional enrichment in each gene list. Table S7 provides GO analysis output by DAVID for each gene list using all genes with raw expression measurements as background.

Subpopulation Enrichment and Depletion Analysis

Under the null hypothesis that there is no association between cluster and anatomic site or psoriatic condition, the number of cells from a given site in a given cluster is a hypergeometric random variable. Mathematically, if X is this number, then X is distributed as follows:

$$P(X=k)=\frac{\binom{K}{k}\binom{N-K}{n-k}}{\binom{N}{n}},$$

where N is the total number of cells, K is the number of cells belonging to the anatomic site or psoriatic condition, and n is the cluster size. We measure the relative enrichment and depletion of a particular site (anatomic or psoriasis) in a particular cluster by the log ratio of the number of observed cells in this cluster to the number expected under the null (hypergeometric) distribution. Significance of the association between each tissue and cluster pair is assessed using Pearson's chi-square test with Bonferroni correction. See also Figures 2 and 7.

RNA Fluorescence *In Situ* Hybridization

For RNA fluorescence *in situ* hybridization (FISH) on formalin-fixed, paraffin-embedded (FFPE) tissue sections, 5-μm sections on glass slides were baked for 1 hr at 60°C, deparaffinized, treated for target retrieval, and applied with protease. Then the FFPE sections were incubated with RNAscope FISH probes (Advanced Cell Diagnostics) and hybridized sequentially to visualize

target RNA signals, according to the RNAscope Fluorescent Multiplex Kit user manual (ACD). Images were obtained using a Zeiss Axio Imager.M2 with a Plan-Apochromat 20×, numerical aperture (NA) = 0.8 objective.

DATA AND SOFTWARE AVAILABILITY

The accession number for the sequence data reported in this paper is European Genome-phenome Archive (EGA): EGAS00001002927. Further information about EGA can be found at https://ega-archive.org.

SUPPLEMENTAL INFORMATION

Supplemental Information includes Supplemental Experimental Procedures, five figures, eight tables, and one data file and can be found with this article online at https://doi.org/10.1016/j.celrep.2018.09.006.

ACKNOWLEDGMENTS

We acknowledge Rachel Sevey and Sarah Pyle for assistance with figure generation using Adobe Illustrator CC. This work was supported in part by funds from NIH grant R01CA163336 and the Grainger Engineering Breakthroughs Initiative (to J.S.S.) and the National Institute of Arthritis and Musculoskeletal and Skin Diseases of the NIH grant K08AR067243 (to J.B.C.). The single-cell concept underlying this work was first presented at the Montagna Symposium on the Biology of Skin 2017 (Precision Dermatology). Publication made possible in part by support from the UCSF Open Access Publishing Fund.

AUTHOR CONTRIBUTIONS

J.B.C. and R.J.C. designed the study. R.C.G., W.L., T.M.M., E.A.K., H.S., I.M.N., R.G., S.S.Y., J.B.C., and R.J.C. supervised sample collection and processing. J.B.C., P.H., J.L., B.E,P.W., and T.M. performed sample preparation and analysis. J.G., M.G., S.C.B., A.J.S., C.J.V., A.I.F., J.S.S., and C.J.V. performed sequencing experiments and computational analyses. S.K. performed in situ hybridization and microscopy analyses under the supervision of J.W.G. J.B.C., A.J.S., A.I.F., M.S.F., J.S.S., and R.J.C. wrote the manuscript with contributions from P.H., J.L., S.K., J.G., M.G., R.G., W.L., B.E.P.W., T.M.M, T.M., E.A.K., H.S., I.M.N., R.C.G, S.S.Y., J.W.G., E.P., R.P., C.J.V., and S.C.B.

DECLARATION OF INTERESTS

The authors declare no competing interests.

Received: March 28, 2018
Revised: June 28, 2018
Accepted: September 4, 2018
Published: October 23, 2018

REFERENCES

Ali, N., Zirak, B., Rodriguez, R.S., Pauli, M.L., Truong, H.-A., Lai, K., Ahn, R., Corbin, K., Lowe, M.M., Scharschmidt, T.C., et al. (2017). Regulatory T Cells in Skin Facilitate Epithelial Stem Cell Differentiation. Cell *169*, 1119–1129.e11.

Benoit, S., Toksoy, A., Ahlmann, M., Schmidt, M., Sunderkötter, C., Foell, D., Pasparakis, M., Roth, J., and Goebeler, M. (2006). Elevated serum levels of calcium-binding S100 proteins A8 and A9 reflect disease activity and abnormal differentiation of keratinocytes in psoriasis. Br. J. Dermatol. *155*, 62–66.

Bolduc, C., Sperling, L.C., and Shapiro, J. (2016). Primary cicatricial alopecia: Lymphocytic primary cicatricial alopecias, including chronic cutaneous lupus erythematosus, lichen planopilaris, frontal fibrosing alopecia, and Graham-Little syndrome. J. Am. Acad. Dermatol. *75*, 1081–1099.

Cheuk, S., Schlums, H., Gallais Sérézal, I., Martini, E., Chiang, S.C., Marquardt, N., Gibbs, A., Detlofsson, E., Introini, A., Forkel, M., et al. (2017). CD49a Expression Defines Tissue-Resident CD8$^+$ T Cells Poised for Cytotoxic Function in Human Skin. Immunity *46*, 287–300.

Cruciat, C.-M., and Niehrs, C. (2013). Secreted and transmembrane wnt inhibitors and activators. Cold Spring Harb. Perspect. Biol. *5*, a015081.

Eckhart, L., Lippens, S., Tschachler, E., and Declercq, W. (2013). Cell death by cornification. Biochim. Biophys. Acta *1833*, 3471–3480.

Geyfman, M., Plikus, M.V., Treffeisen, E., Andersen, B., and Paus, R. (2015). Resting no more: re-defining telogen, the maintenance stage of the hair growth cycle. Biol. Rev. Camb. Philos. Soc. *90*, 1179–1196.

Gilchrest, B.A. (1983). In vitro assessment of keratinocyte aging. J. Invest. Dermatol. *81* (*1, Suppl*), 184s–189s.

Goldsmith, L.A., Katz, S.I., Gilchrest, B.A., Paller, A.S., Leffell, D.J., and Wolff, K. (2012). Fitzpatrick's Dermatology in General Medicine, Eighth Edition (McGraw-Hill Education/Medical).

Grehan, S., Allan, C., Tse, E., Walker, D., and Taylor, J.M. (2001). Expression of the apolipoprotein E gene in the skin is controlled by a unique downstream enhancer. J. Invest. Dermatol. *116*, 77–84.

Hinde, E., Haslam, I.S., Schneider, M.R., Langan, E.A., Kloepper, J.E., Schramm, C., Zouboulis, C.C., and Paus, R. (2013). A practical guide for the study of human and murine sebaceous glands in situ. Exp. Dermatol. *22*, 631–637.

Hoashi, T., Watabe, H., Muller, J., Yamaguchi, Y., Vieira, W.D., and Hearing, V.J. (2005). MART-1 is required for the function of the melanosomal matrix protein PMEL17/GP100 and the maturation of melanosomes. J. Biol. Chem. *280*, 14006–14016.

Hsieh, W.-L., Huang, Y.-H., Wang, T.-M., Ming, Y.-C., Tsai, C.-N., and Pang, J.-H.S. (2015). IFI27, a novel epidermal growth factor-stabilized protein, is functionally involved in proliferation and cell cycling of human epidermal keratinocytes. Cell Prolif. *48*, 187–197.

Huang, W., Sherman, B.T., and Lempicki, R.A. (2009a). Bioinformatics enrichment tools: paths toward the comprehensive functional analysis of large gene lists. Nucleic Acids Res. *37*, 1–13.

Huang, W., Sherman, B.T., and Lempicki, R.A. (2009b). Systematic and integrative analysis of large gene lists using DAVID bioinformatics resources. Nat. Protoc. *4*, 44–57.

Jong, M.C., Gijbels, M.J., Dahlmans, V.E., Gorp, P.J., Koopman, S.J., Ponec, M., Hofker, M.H., and Havekes, L.M. (1998). Hyperlipidemia and cutaneous abnormalities in transgenic mice overexpressing human apolipoprotein C1. J. Clin. Invest. *101*, 145–152.

Joost, S., Zeisel, A., Jacob, T., Sun, X., La Manno, G., Lönnerberg, P., Linnarsson, S., and Kasper, M. (2016). Single-Cell Transcriptomics Reveals that Differentiation and Spatial Signatures Shape Epidermal and Hair Follicle Heterogeneity. Cell Syst. *3*, 221–237.e9.

Kentala, H., Koponen, A., Kivelä, A.M., Andrews, R., Li, C., Zhou, Y., and Olkkonen, V.M. (2018). Analysis of ORP2-knockout hepatocytes uncovers a novel function in actin cytoskeletal regulation. FASEB J. *32*, 1281–1295.

Kwon, S., Chin, K., Nederlof, M., and Gray, J.W. (2017). Quantitative, in situ analysis of mRNAs and proteins with subcellular resolution. Sci. Rep. *7*, 16459.

Law, C.W., Chen, Y., Shi, W., and Smyth, G.K. (2014). voom: Precision weights unlock linear model analysis tools for RNA-seq read counts. Genome Biol. *15*, R29.

Lessard, J.C., Piña-Paz, S., Rotty, J.D., Hickerson, R.P., Kaspar, R.L., Balmain, A., and Coulombe, P.A. (2013). Keratin 16 regulates innate immunity in response to epidermal barrier breach. Proc. Natl. Acad. Sci. USA *110*, 19537–19542.

Lim, X., Tan, S.H., Yu, K.L., Lim, S.B.H., and Nusse, R. (2016). Axin2 marks quiescent hair follicle bulge stem cells that are maintained by autocrine Wnt/β-catenin signaling. Proc. Natl. Acad. Sci. USA *113*, E1498–E1505.

Macosko, E.Z., Basu, A., Satija, R., Nemesh, J., Shekhar, K., Goldman, M., Tirosh, I., Bialas, A.R., Kamitaki, N., Martersteck, E.M., et al. (2015). Highly Parallel Genome-wide Expression Profiling of Individual Cells Using Nanoliter Droplets. Cell *161*, 1202–1214.

Malinauskas, T., Aricescu, A.R., Lu, W., Siebold, C., and Jones, E.Y. (2011). Modular mechanism of Wnt signaling inhibition by Wnt inhibitory factor 1. Nat. Struct. Mol. Biol. *18*, 886–893.

Mitoma, C., Kohda, F., Mizote, Y., Miake, A., Ijichi, A., Kawahara, S., Kohno, M., Sonoyama, H., Mitamura, Y., Kaku, Y., et al. (2014). Localization of S100A2, S100A4, S100A6, S100A7, and S100P in the human hair follicle. Fukuoka Igaku Zasshi Hukuoka Acta Medica *105*, 148–156.

Ohyama, M., Terunuma, A., Tock, C.L., Radonovich, M.F., Pise-Masison, C.A., Hopping, S.B., Brady, J.N., Udey, M.C., and Vogel, J.C. (2006). Characterization and isolation of stem cell-enriched human hair follicle bulge cells. J. Clin. Invest. *116*, 249–260.

Richard, G., Rouan, F., Willoughby, C.E., Brown, N., Chung, P., Ryynänen, M., Jabs, E.W., Bale, S.J., DiGiovanna, J.J., Uitto, J., and Russell, L. (2002). Missense mutations in GJB2 encoding connexin-26 cause the ectodermal dysplasia keratitis-ichthyosis-deafness syndrome. Am. J. Hum. Genet. *70*, 1341–1348.

Risso, D., Perraudeau, F., Gribkova, S., Dudoit, S., and Vert, J.-P. (2018). A general and flexible method for signal extraction from single-cell RNA-seq data. Nat. Commun. *9*, 284.

Ritchie, M.E., Phipson, B., Wu, D., Hu, Y., Law, C.W., Shi, W., and Smyth, G.K. (2015). limma powers differential expression analyses for RNA-sequencing and microarray studies. Nucleic Acids Res. *43*, e47.

Ruano, J., Suárez-Fariñas, M., Shemer, A., Oliva, M., Guttman-Yassky, E., and Krueger, J.G. (2016). Molecular and Cellular Profiling of Scalp Psoriasis Reveals Differences and Similarities Compared to Skin Psoriasis. PLoS ONE *11*, e0148450.

Skelly, D.A., Squiers, G.T., McLellan, M.A., Bolisetty, M.T., Robson, P., Rosenthal, N.A., and Pinto, A.R. (2018). Single-Cell Transcriptional Profiling Reveals Cellular Diversity and Intercommunication in the Mouse Heart. Cell Rep. *22*, 600–610.

Soneson, C., and Robinson, M.D. (2018). Bias, robustness and scalability in single-cell differential expression analysis. Nat. Methods *15*, 255–261.

Stoll, S.W., Stuart, P.E., Lambert, S., Gandarillas, A., Rittié, L., Johnston, A., and Elder, J.T. (2016). Membrane-Tethered Intracellular Domain of Amphiregulin Promotes Keratinocyte Proliferation. J. Invest. Dermatol. *136*, 444–452.

Street, K., Risso, D., Fletcher, R.B., Das, D., Ngai, J., Yosef, N., Purdom, E., and Dudoit, S. (2018). Slingshot: cell lineage and pseudotime inference for single-cell transcriptomics. BMC Genomics *19*, 477.

Sun, L.-D., Cheng, H., Wang, Z.-X., Zhang, A.-P., Wang, P.-G., Xu, J.-H., Zhu, Q.-X., Zhou, H.-S., Ellinghaus, E., Zhang, F.-R., et al. (2010). Association analyses identify six new psoriasis susceptibility loci in the Chinese population. Nat. Genet. *42*, 1005–1009.

Suomela, S., Cao, L., Bowcock, A., and Saarialho-Kere, U. (2004). Interferon alpha-inducible protein 27 (IFI27) is upregulated in psoriatic skin and certain epithelial cancers. J. Invest. Dermatol. *122*, 717–721.

Tapia, R., Kralicek, S.E., and Hecht, G.A. (2017). EPEC effector EspF promotes Crumbs3 endocytosis and disrupts epithelial cell polarity. Cell. Microbiol. Published online July 27, 2017. https://doi.org/10.1111/cmi.12757.

Troyanovsky, S.M., Guelstein, V.I., Tchipysheva, T.A., Krutovskikh, V.A., and Bannikov, G.A. (1989). Patterns of expression of keratin 17 in human epithelia: dependency on cell position. J. Cell Sci. *93*, 419–426.

Tsang, J.C.H., Vong, J.S.L., Ji, L., Poon, L.C.Y., Jiang, P., Lui, K.O., Ni, Y.-B., To, K.F., Cheng, Y.K.Y., Chiu, R.W.K., and Lo, Y.M.D. (2017). Integrative single-cell and cell-free plasma RNA transcriptomics elucidates placental cellular dynamics. Proc. Natl. Acad. Sci. USA *114*, E7786–E7795.

van Dijk, D., Nainys, J., Sharma, R., Kathail, P., Carr, A.J., Moon, K.R., Mazutis, L., Wolf, G., Krishnaswamy, S., and Pe'er, D. (2017). MAGIC: A diffusion-based imputation method reveals gene-gene interactions in single-cell RNA-sequencing data. bioRxiv. https://doi.org/10.1101/111591.

Veniaminova, N.A., Vagnozzi, A.N., Kopinke, D., Do, T.T., Murtaugh, L.C., Maillard, I., Dlugosz, A.A., Reiter, J.F., and Wong, S.Y. (2013). Keratin 79 identifies a novel population of migratory epithelial cells that initiates hair canal morphogenesis and regeneration. Development *140*, 4870–4880.

Warzecha, C.C., Jiang, P., Amirikian, K., Dittmar, K.A., Lu, H., Shen, S., Guo, W., Xing, Y., and Carstens, R.P. (2010). An ESRP-regulated splicing programme is abrogated during the epithelial-mesenchymal transition. EMBO J. *29*, 3286–3300.

Wolf, R., Howard, O.M.Z., Dong, H.-F., Voscopoulos, C., Boeshans, K., Winston, J., Divi, R., Gunsior, M., Goldsmith, P., Ahvazi, B., et al. (2008). Chemotactic activity of S100A7 (Psoriasin) is mediated by the receptor for advanced glycation end products and potentiates inflammation with highly homologous but functionally distinct S100A15. J. Immunol. *181*, 1499–1506.

Xing, L., Dai, Z., Jabbari, A., Cerise, J.E., Higgins, C.A., Gong, W., de Jong, A., Harel, S., DeStefano, G.M., Rothman, L., et al. (2014). Alopecia areata is driven by cytotoxic T lymphocytes and is reversed by JAK inhibition. Nat. Med. *20*, 1043–1049.

Yan, D., Huang, L., and Jordan, M.I. (2009). Fast Approximate Spectral Clustering. In Proceedings of the 15th ACM SIGKDD International Conference on Knowledge Discovery and Data Mining (Association for Computing Machinery), pp. 907–916.

Yang, H., Adam, R.C., Ge, Y., Hua, Z.L., and Fuchs, E. (2017). Epithelial-Mesenchymal Micro-niches Govern Stem Cell Lineage Choices. Cell *169*, 483–496.e13.

Zaba, L.C., Fuentes-Duculan, J., Eungdamrong, N.J., Abello, M.V., Novitskaya, I., Pierson, K.C., Gonzalez, J., Krueger, J.G., and Lowes, M.A. (2009). Psoriasis is characterized by accumulation of immunostimulatory and Th1/Th17 cell-polarizing myeloid dendritic cells. J. Invest. Dermatol. *129*, 79–88.

Zheng, G.X.Y., Terry, J.M., Belgrader, P., Ryvkin, P., Bent, Z.W., Wilson, R., Ziraldo, S.B., Wheeler, T.D., McDermott, G.P., Zhu, J., et al. (2017). Massively parallel digital transcriptional profiling of single cells. Nat. Commun. *8*, 14049.

Cell Reports, Volume 25

Supplemental Information

Transcriptional Programming of Normal and Inflamed Human Epidermis at Single-Cell Resolution

Jeffrey B. Cheng, Andrew J. Sedgewick, Alex I. Finnegan, Paymann Harirchian, Jerry Lee, Sunjong Kwon, Marlys S. Fassett, Justin Golovato, Matthew Gray, Ruby Ghadially, Wilson Liao, Bethany E. Perez White, Theodora M. Mauro, Thaddeus Mully, Esther A. Kim, Hani Sbitany, Isaac M. Neuhaus, Roy C. Grekin, Siegrid S. Yu, Joe W. Gray, Elizabeth Purdom, Ralf Paus, Charles J. Vaske, Stephen C. Benz, Jun S. Song, and Raymond J. Cho

Supplemental Figures

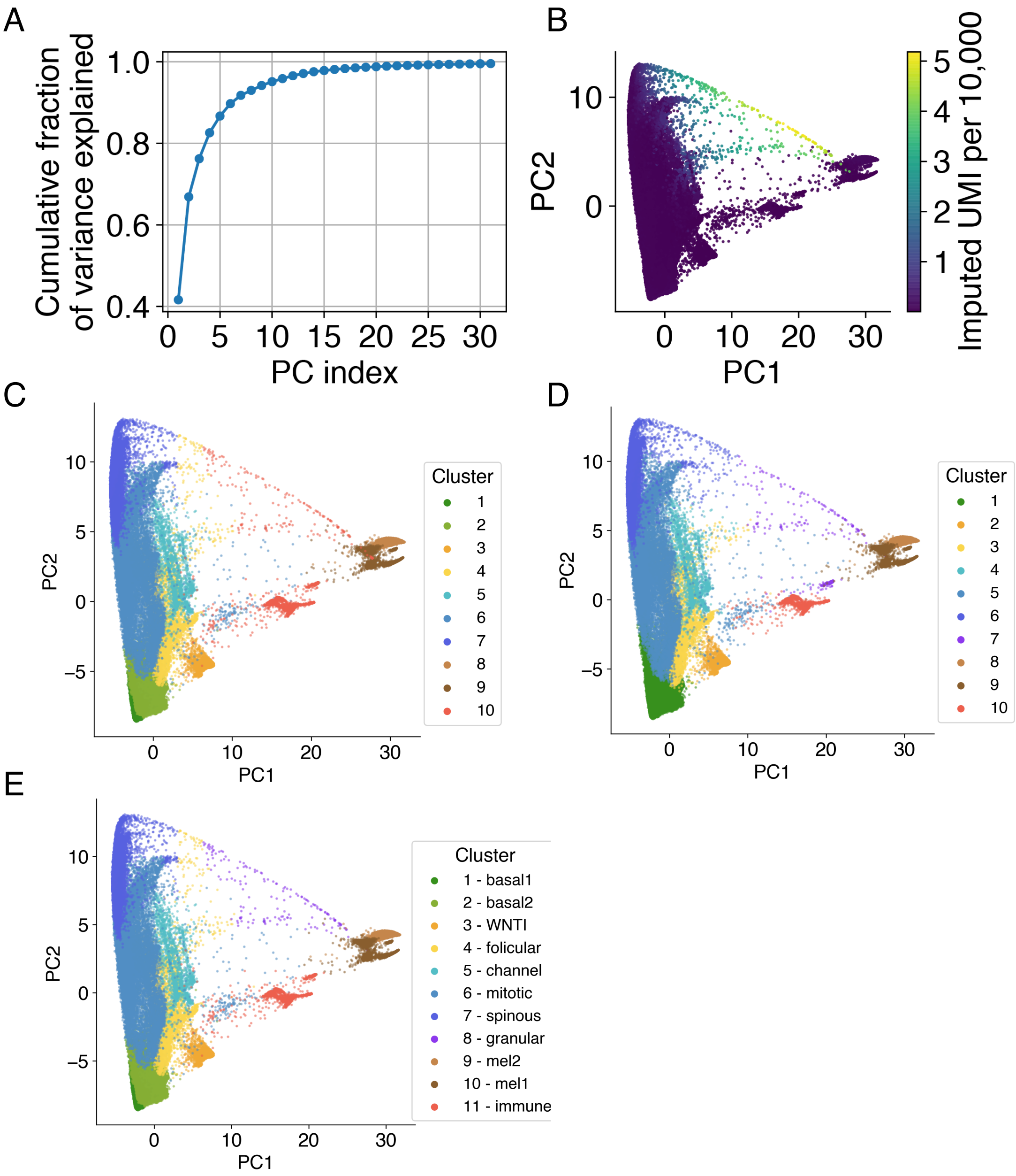

Figure S1 Spectral Clustering Details, Related to Figure 1
Details on spectral clustering of imputed expression. (A) Cumulative fraction of variance explained in PCA analysis of log transformed expression of robustly expressed genes in healthy tissue. (B) Principal components plot colored by imputed expression of late keratinocyte differentiation marker LOR. (C) Approximate spectral clustering with adaptive distance parameters ka=10 and k=30 combines late keratinocytes and immune cells in cluster 10. (D) Approximate spectral clustering with adaptive distance parameters ka=3 and k=10, favoring a more local notion of cell similarity, separates late keratinocytes and immune cells. (E) Principal components plot colored by cluster after separating panel C cluster 10 with second round of spectral clustering.

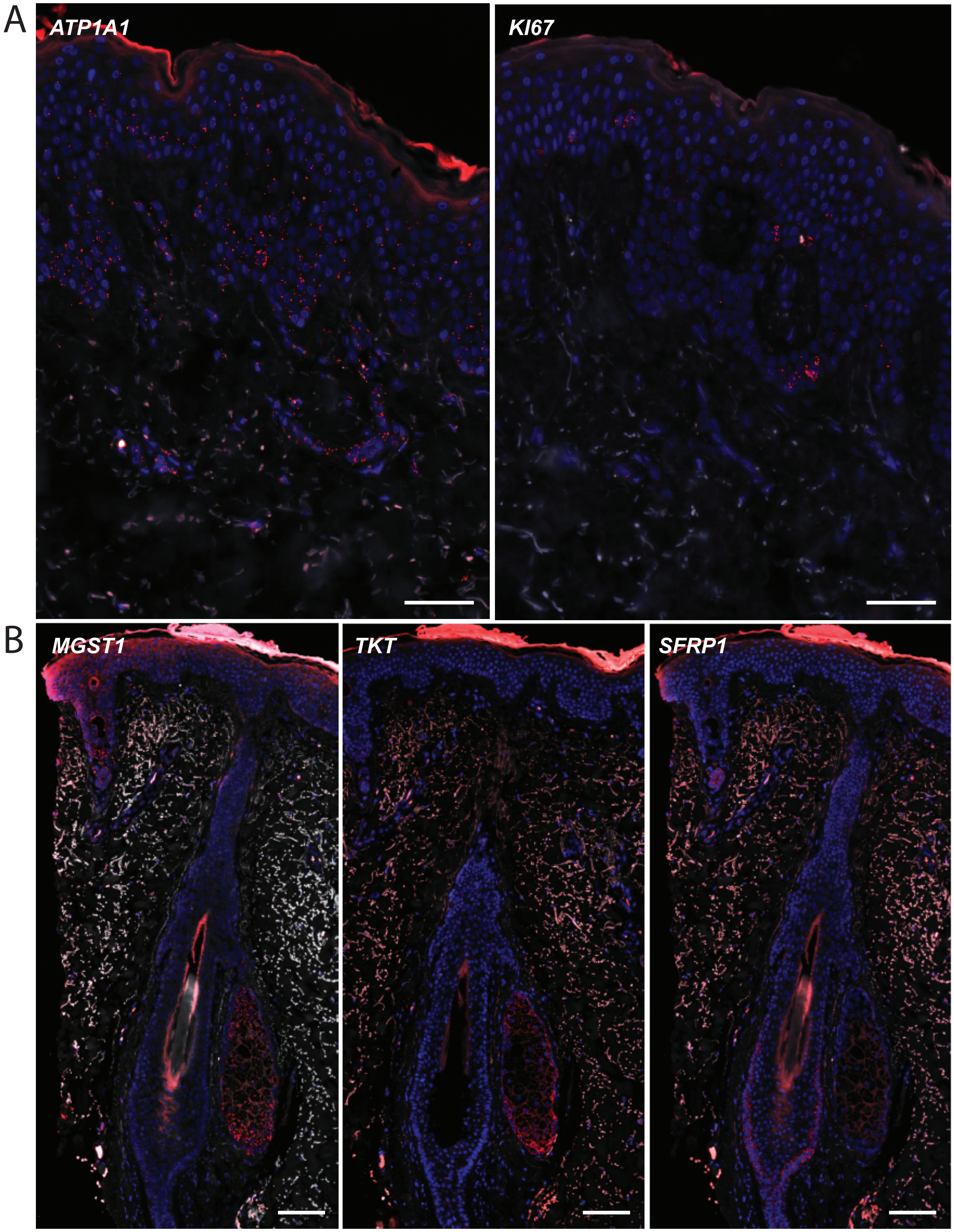

Figure S2 RNA *in situ* hybridization, Related to Figure 1, 3, and 4
(A) RNA *in situ hybridization* staining of trunk skin for *KI67* and *ATP1A1* (red channel). (B) RNA *in situ hybridization* staining (red channel) of scalp skin for hair subpopulation markers *MGST1, TKT,* and *SFRP1.* Blue channel is DAPI staining. Scale bars, 50 μM for A and 100 μM for B.

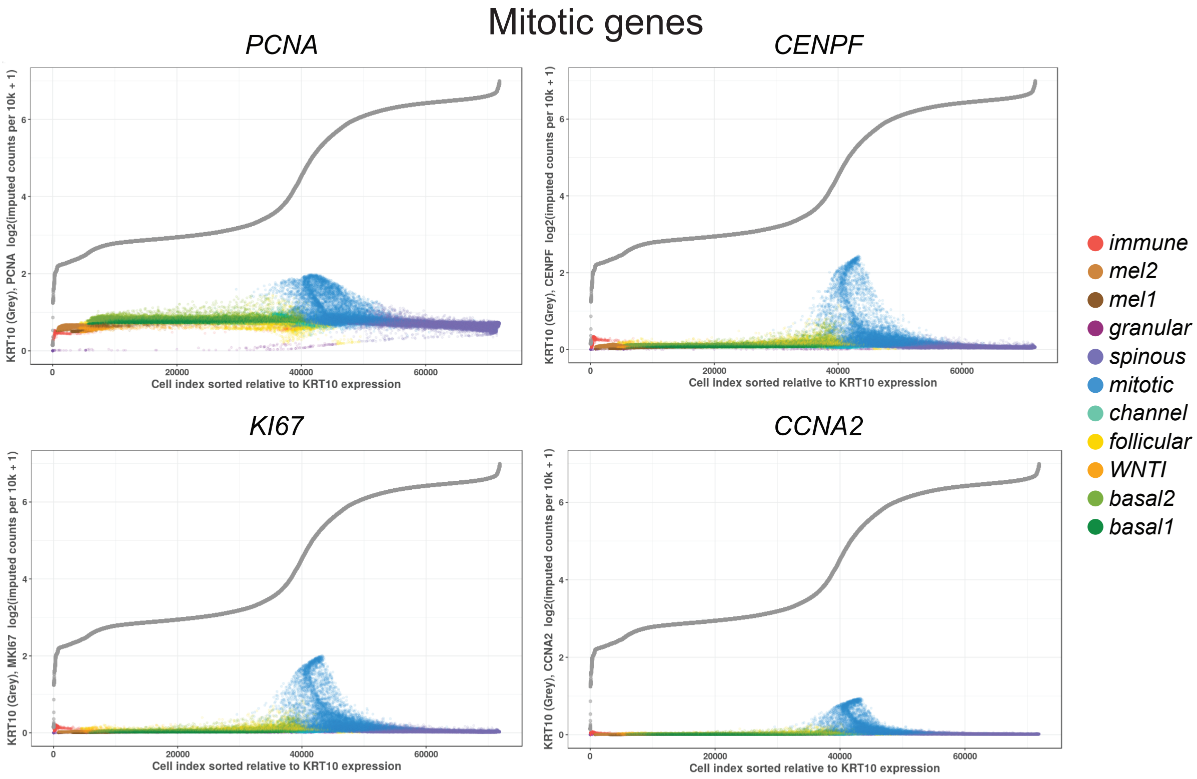

Figure S3 Variance of mitotic and potential progenitor genes with *KRT10*, Related to Figure 3
Imputed expression of mitotic cell cycle genes (*PCNA*, *CENPF*, *KI67*, and *CCNA2*) pseudocolored by cluster and plotted with *KRT10* imputed expression in grey (both expression values on y-axis). Cells are indexed along the x-axis by low to high *KRT10* expression.

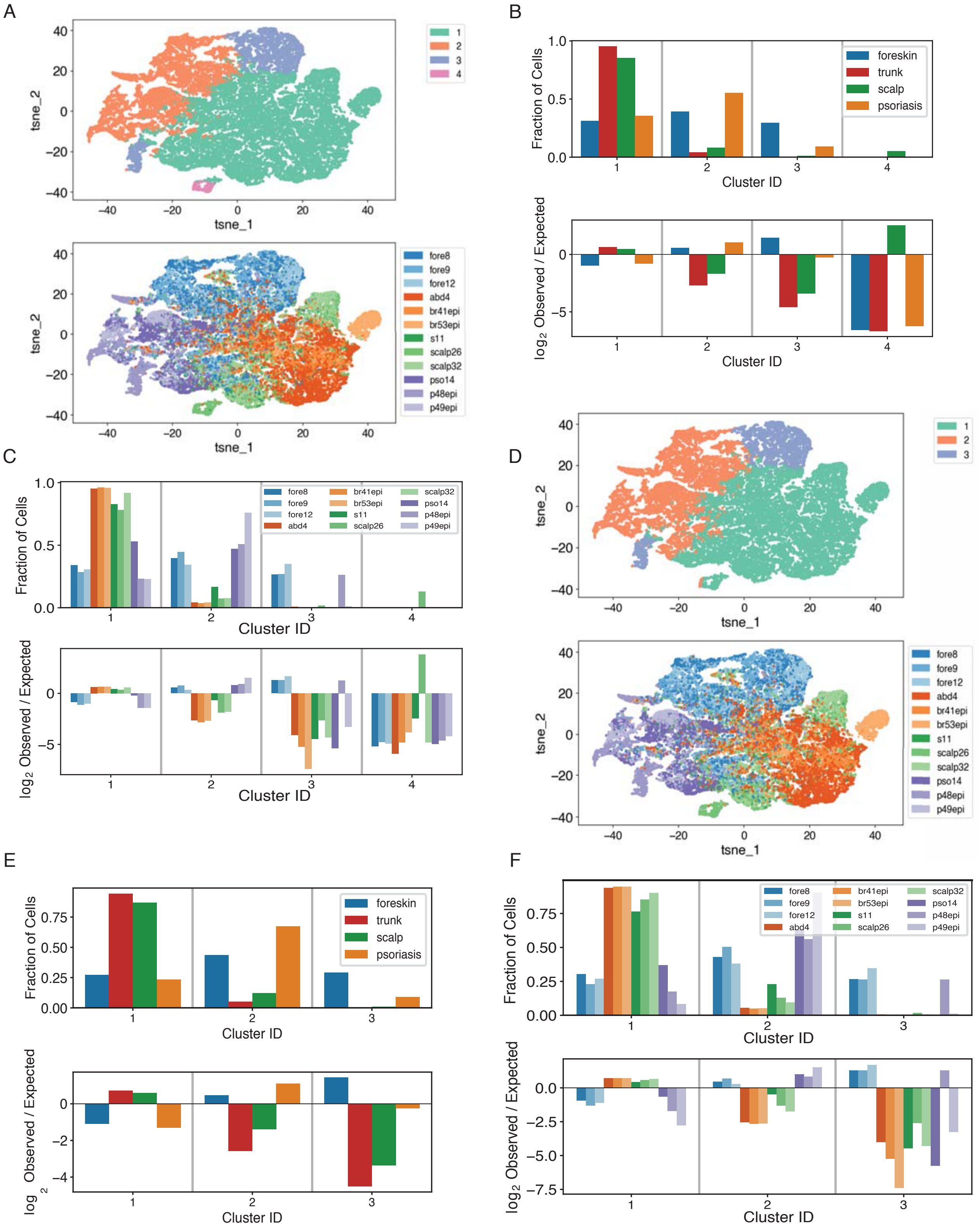
A
B
C
D
E
F
tsne_1
tsne_2
1
2
3
4
fore8
fore9
fore12
abd4
br41epi
br53epi
s11
scalp26
scalp32
pso14
p48epi
p49epi
foreskin
trunk
scalp
psoriasis
Fraction of Cells
$\log_2$ Observed / Expected
Cluster ID

Figure S4 Re-clustering of basal keratinocytes into 3 and 4 clusters, Related to Figure 1
(A) tSNE plots showing subclustering of 2 basal clusters (basal1 and basal2) into 4 total clusters (top pseudocolored by cluster, bottom pseudocolored by sample). (B) Fraction of cells from each anatomic site belonging to each cluster by tissue (top) or Log ratio of observed number of cells from anatomic site in cluster to expected number if sampling cells in cluster uniformly without replacement (bottom). Positive/negative log ratios indicate cluster enrichment/depletion for anatomic site or sample. Pseudo-counts of 1 are added to numerator of log-ratios when observed co-occurence is zero. All tissue and cluster associations are significant ($padj < 0.05$, Person chi square test with Bonferroni adjustment). (C). Same as B except for each individual sample, rather than anatomic site. Note that cluster 4 is almost entirely composed of cells from *scalp26* sample. (D-F) same as (A-C) except showing subclustering into 3 total clusters.

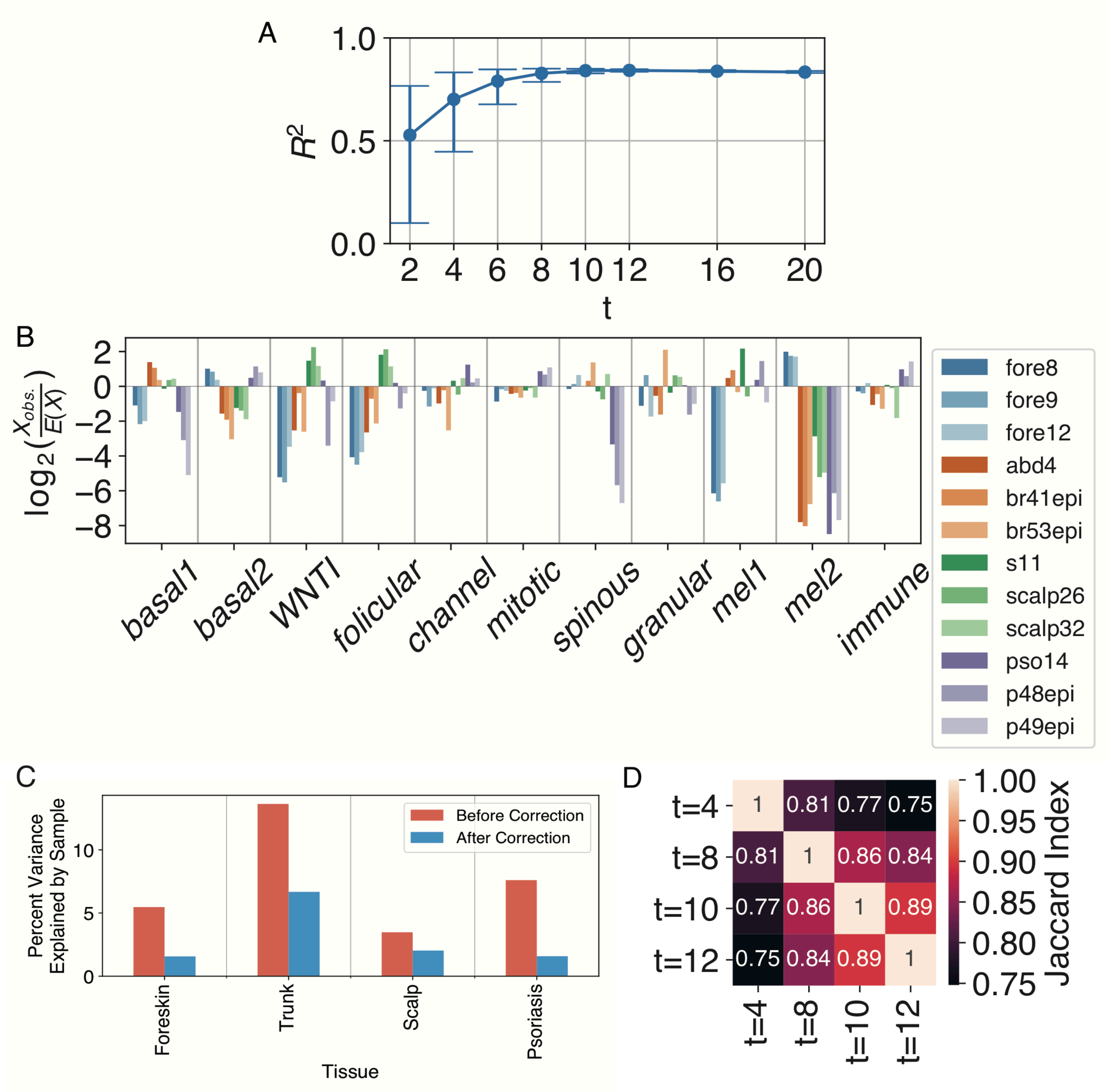

Figure S5 Choice of MAGIC t parameter and variance explained by batch, Related to Figure 1

Choice of MAGIC t parameter and variance explained by batch before and after correction. (A) Square of Pearson correlation coefficient between artificial dropout targets and imputation results for MAGIC algorithm with PCA based similarity. Each t value is described by min, max and mean for 3 replicates of the artificial dropout procedure. (B) Batches are enriched or depleted in clusters according to their anatomical origin. A pseudo-count of 1 is added to allow illustration of depletion when a batch has zero occurrence in a cluster. (C) Percent of variance in gene expression explained by sample within each tissue type. Percentage is calculated as $R^2 \times 100$, summing total and residual sums of squares over 12,783 genes expressed in more than one percent of cells. We used a similar method to quantify biological variation after batch correction and imputation and found that 10.5% of variation in expression is explained by tissue/disease state. (D) Jaccard Indices measuring agreement between KASP clustering (10 clusters ka=10 , k=30) for range of t values.

Supplemental Experimental Procedures

Data processing and QC filtering

Cellranger (10X genomics version 2.0.2) was used to de-multiplex the raw Illumina data, quantify UMIs and aggregate data from our 12 samples. We used the GRCh38 version 1.2.0 reference transcriptome provided by 10X genomics for quantification. We used the default read depth normalization mode for the aggregation step which subsamples reads from higher-depth samples to match the number of confidently mapped reads per cell across samples. We used Seurat (version 2.2.0) (Macosko et al., 2015) to manage and interact with scRNA expression profiles from 116,497 cells from the 12 libraries produced by our sequencing experiments. We performed filtering on the data using percent mitochondrial UMI and number of transcripts with UMI > 0 as quality control metrics. A high proportion of mitochondrial expression is indicative of cells damaged during isolation so we filtered out cells in the top 5th percentile of proportion of mitochondrial UMI, which corresponded to cells with greater than 9.5% of total UMI accounted for by mitochondrial transcripts. To filter on number of detected transcripts, we removed cells in the bottom 0.5% (fewer than 928 transcripts detected) and top 15% (more than 3441 detected) of cells based on this metric, in order to avoid including partial cells and doublets respectively in our analysis. These filters left 92,889 cells to include in our primary analyses.

Imputation

We used the low dimensional representation of cell expression profiles output by ZINB-WaVE (Risso et al., 2018) (version 1.0.0). This representation was used in a modified version of the MAGIC algorithm (van Dijk et al., 2018), described below, to mitigate the effects of scRNA-seq dropout by sharing expression information among cells with similar corrected expression profiles.

Specifically, we used ZINB-WaVE (version 1.0.0) to model, for each gene in each cell, the mean expression and probability of dropout as functions of cell-level covariates: percent mitochondrial UMI, total UMI, batch, and 20 latent cell-level features learned from the raw expression matrix. With this choice of covariates, ZINB-WaVE fits the model:

$$\begin{aligned} \ln(\mu_{i,j}) &= X\beta_u + \left(V\gamma_\mu\right)^T + W\alpha_\mu \\ \text{logit}(\pi_{i,j}) &= X\beta_\pi + (V\gamma_\pi)^T + W\alpha_\pi \\ \ln(\theta_{i,j}) &= \zeta_j, \end{aligned}$$

where, adopting the notation of Risso et al. (2018), $\mu_{i,j}$ and $\pi_{i,j}$ are, respectively, the mean of the negative binomial distribution and the inflated probability of zero expression for the jth gene ($j \in \{1, \dots, J\}$) in the ith cell ($i \in \{1, \dots, n\}$), X is an (n , 14) matrix encoding the known cell level covariates and including a column of 1's, V is a (J, 1) matrix of 1's, and W is a (n, 20) matrix of latent cell-level features. The matrices β_μ , β_π , γ_μ and γ_π with shapes (14, J), (14, J), (1, n), (1, n), respectively, are regression parameters and, α_μ and α_π are (20, J) loading matrices associated with W. Finally, $\theta_{i,j}$ is the inverse dispersion parameter for the negative binomial distribution described by the single parameter ζ_j for each gene.

Covariates in matrices X and V explained unwanted sources of variation in measured gene expression across cells. The matrix W captured the remaining low dimensional variation of interest, with each row providing a 20-dimensional description of a cell's expression state. These rows were used to calculate cell-cell similarities needed to impute expression using the MAGIC algorithm.

Our version of MAGIC, modified to include only wanted sources of variation captured by ZINB-WaVE, is as follows. First, we constructed the MAGIC affinity matrix, using adaptive distance parameters ka = 10 and k = 30 and measuring cell-cell distances with a Euclidean metric on the corresponding rows of the ZINB-WaVE W matrix. That is, we simply replaced the principal component coordinates in the typical MAGIC affinity matrix construction with entries of the ZINB-WaVE W matrix. This custom affinity matrix and the (n, J) matrix R of raw counts were then input to a protocol exactly following the MAGIC algorithm (version 0.0).

Specifically, we calculated the library size normalized raw expression matrix D as

$$D = \frac{R_{i,j}}{\sum_{j=1}^{J} R_{i,j}} \underset{i}{\text{median}} \left(\sum_{j=1}^{J} R_{i,j} \right)$$

and formed the Markov affinity matrix M by row normalizing the affinity matrix constructed from W. From these matrices, imputed expression is given by

$$D_{imputed} = M^t D$$

where the "diffusion time" t is an integer chosen to yield good recovery of simulated drop-out events on our data set (see Supplementary Experimental Procedures: Choice of MAGIC t parameter"). Finally, the MAGIC protocol dictates rescaling the imputed expression values via:

$$(D_{rescale})_{i,j} = (D_{imputed})_{i,j} \frac{\underset{i}{\text{percentile}}(D_{i,j}, 99)}{\underset{i}{\max}((D_{imputed})_{i,j})};$$

that is, imputed expression values were rescaled so that the max imputed expression for each gene matched the 99th percentile of the pre-imputed expression. The matrix $D_{rescale}$ is the imputed result of the MAGIC protocol; however, because it is not guaranteed to be normalized to a common cell library size, we renormalize each row of $D_{rescale}$ to units of imputed expression per 10,000. The resulting expression matrix E, with genes in columns and cells in rows, is the output of our imputation procedure.

Principal component analysis

We used principal component analysis (PCA) on imputed expression of robustly expressed genes to obtain a low dimensional representation of cells according to coordinated variation in gene expression. Specifically, we first restricted to columns of E corresponding to genes robustly expressed in the raw data (>= 5 UMI in >= 100 cells). We then partitioned the resulting matrix, with 2468 genes, into sub-matrices E_{health} corresponding to cells from foreskin, scalp and trunk samples, and E_{pso} corresponding to cells from psoriasis samples. We $\log_2$ transformed entries of E_{health} (with pseudo-count 1) mean centered genes and performed PCA, describing healthy cells by their coordinates along the first 20 principal components (PCs). These PCs sufficed to capture nearly all the variation in our imputed data (Figure S1A). To represent psoriasis cells in this space, we applied the same $\log_2$ transformation and constant shift and projected the transformed psoriasis cell expression vectors onto the 20 PCs. The resulting 20-dimensional cell representations were used as input for t-SNE and multi-tissue clustering of cells.

Spectral clustering

We used spectral clustering of healthy cells represented in the aforementioned 20 PC space to identify similar phenotypic states. Spectral clustering uses eigen-decomposition of a cell-cell similarity matrix to group cells in a manner that depends locally on distance but globally on the distribution of cells in the ambient expression space. Thus, spectral clustering is less biased towards ellipsoidal clusters often identified by purely distance-based clustering algorithms. To reduce the computational cost of diagonalizing a large cell-cell similarity matrix, we employed a modified form of the k-means-based approximate spectral clustering (KASP) algorithm (Yan et al., 2009). Given a matrix $\mathcal{E}_{health}$, with PC coordinates of cells in rows and shape $(71864, 20)$, the number n_{clust} of clusters, and a data reduction factor, alpha, of 10 -- so that spectral clustering is performed on $\text{floor}(71864/10) = 7186$ observations -- the KASP algorithm is:

1. Select $n_{k-means}$ rows from $\mathcal{E}_{health}$, randomly and without replacement, and perform k-means clustering of theses rows identifying 7186 centroids. (Our implementation used $n_{k-means} = 0.5 \times 71864 = 35932$ and took the best k-means results from 20 centroid initializations)
2. Perform a second round of k-means clustering of all 71864 cells initializing centroids at the best result from step 1.
3. Perform spectral clustering of the 7186 centroids from step 2 and identify n_{clust} clusters.
4. Assign each cell to the spectral cluster label of its corresponding k-means centroid from step 2.

Step 3 requires specification of a similarity matrix and type of graph Laplacian constructed from this matrix. We obtained the similarity matrix among k-means centroids using the same construction as for the MAGIC affinity matrix with Euclidean distance measured between centroid representations in 20-dimensional PC space and adaptive similarity parameters ka = 10 and k = 30. From this similarity matrix we construct the random-walk graph Laplacian and perform the Shi and Malik version of spectral clustering (Shi and Malik, 2000) as described by von Luxburg (2007). After assigning cluster labels to all healthy cells, psoriasis cells are assigned to clusters via scikit-learn's (version 0.19.0) KNeighborsClassifier fit on healthy cells represented in the 20 PC space and using 10 nearest neighbors with other parameters set to default values (Pedregosa et al., 2011).

We observed that the resulting clustering mixed a small number (approximately 200) of late keratinocytes with the primary immune cell cluster (Figure S1B and C). In addition to this important biological distinction, we observed that making the adaptive cell-cell similarity parameters used for clustering more local (decreasing ka and k from 10 to 3 and from 30 to 10, respectively) yielded clustering that successfully partitioned the problematic cluster (Figure S1D). Given these biological and data-driven motivations, we isolated the cells in the immune cell cluster (Figure

S1C, cluster 10) and used spectral clustering (without the k-means based approximation) to split this cluster in two. The resulting clusters are illustrated in Figure S1E and used throughout the main text.

Secondary clustering of basal keratinocytes, scalp keratinocytes, and immune cells

Secondary spectral clustering was performed on three subsets of cells. The first subset consisted of cells assigned to *basal1* and *basal2* in primary spectral clustering; the second subset consisted of cells from the three scalp samples not assigned to the *mel1*, *mel2*, and *immune* cell clusters; and, the third subset consisted of immune cells from primary spectral clustering. We used the method of Supplemental Experimental Procedures: Principal component analysis, restricted to each of these subsets, to obtain 20-dimensional cell representations. Representations of data in each subset were clustered using the KASP algorithm with parameters alpha=2, ka=10, k=20. Secondary clustering of the *basal1* and *basal2* subset tested n_{clust} between 2 and 10. Secondary clustering of scalp and immune subsets tested n_{clust} = 10, 15, and 20.

Choice of MAGIC t parameter

MAGIC's diffusion time parameter t controls the extent of neighbors in the weighted cell-cell similarity graph over which cell expression vectors are averaged to yield imputed expression. Increasing t decreases locality and increases the number of similar cells used in the average. Our modified version of the MAGIC algorithm used this averaging method to: first, reduce the effect of dropout in raw data and, second, remove patterns of variation in raw data attributable to unwanted sources. The second objective was achieved by using ZINB-WaVE's W matrix in our construction of the cell-cell similarity graph, so that MAGIC removes potential batch effects by averaging raw expression vectors over cells having similar corrected low-dimensional representations in W.

To choose t, we simulated MAGIC's ability to recover dropout values in our raw expression matrix R. Because the cell-cell similarity information contained in ZINB-WaVE's W matrix was based on the entire data matrix R without simulated dropouts, using the W matrix as an input would over-estimate the true recovery rate. Thus, we instead used the original MAGIC algorithm to choose a value of t yielding good simulated dropout recovery. We used this t in our modified version of MAGIC and verified the absence of batch effect in clustering of the resulting imputed data.

Specifically, we sampled (for computational efficiency) 8,000 foreskin cells at random and formed the raw expression matrix R_{sub} for these cells and for genes with at least 1 UMI in 1% of these cells. We randomly sampled 20% (~3,800,000) of the non-zero entries of R_{sub} as dropout events. Denoting the set of selected (row, column) dropout pairs by S, the targets for imputation recovery were

$$T = \left((D_{sub})_{i,j} : (i,j) \in S\right)$$

where D_{sub} is the library normalized raw expression matrix

$$D_{sub} = \frac{(R_{sub})_{i,j}}{\sum_{j=1}^{J} (R_{sub})_{i,j}} \operatorname*{median}_{i}\left(\sum_{j=1}^{J} (R_{sub})_{i,j}\right).$$

We then set $(R_{sub})_{i,j} = 0$ for all $(i,j) \in S$ and called the resulting corrupted matrix $\mathcal{R}$. We ran the standard MAGIC algorithm on $\mathcal{R}$, calculating the library normalized matrix $\mathcal{D}$ from $\mathcal{R}$ and then constructing the MAGIC affinity matrix from Euclidean distances between cells represented in the space spanned by the 20 highest-variance PCs, where PCA was performed on the $\log_2$ transformed (with pseudo-count 1) and mean centered $\mathcal{D}$ matrix. We measured recovery using R^2 (the square of Pearson correlation coefficient) between the sequences T and $((\mathcal{D}_{rescale})_{i,j} : (i,j) \in S)$, where $\mathcal{D}_{rescale}$ is the output of the MAGIC algorithm (described in Supplemental Experimental Procedures: Imputation). Based on the results in Figure S5A, we identified $t = 10$ as a candidate parameter with good recovery.

To verify that $t = 10$ removed unwanted batch effects in imputed expression, we performed imputation on the full data set using our modified MAGIC algorithm and examined the enrichment/depletion of independent samples in each of the KASP clusters (adaptive distance parameters ka=10, k=30). The samples were enriched or depleted in clusters according to their anatomical origin, rather than experimental batch, implying that the choice of diffusion time $t = 10$ successfully removed unwanted batch artifacts (Figure S5B).

We quantified the batch correction performance by calculating the percentage of variance in gene expression ($R^2 \times 100$) explained by sample within each tissue type, before and after application of the ZINB-WaVE/MAGIC

algorithm (Figure S5C). Quantification via R^2 used total and residual sums of squares taken over 12,783 genes expressed in more than one percent of cells. We used a similar method to quantify biological variation after batch correction and found that 10.5% of variation in expression is explained by tissue/disease state.

Finally, we demonstrated the stability of clustering results against changes in t. We ran our modified MAGIC algorithm on the full data set with $t = 4, 8, 10, 12$, clustered each imputed result into 10 clusters with KASP (same parameters as in Supplemental Experimental Procedures: Spectral clustering) and measured the clustering concordance using the Jaccard index. The Jaccard Index between clusterings A and B ranges between 0 (no agreement) and 1 (perfect agreement) and is the ratio of the number of cell pairs co-clustered in both A and B to the number of cell pairs co-clustered in either A or B. The clustering results did not change appreciably as t was varied between 8 and 12 (Figure S5D).

Pseudotime
We used Slingshot (Street et al., 2018) (version 0.1.2-1) to infer developmental lineages of scalp keratinocytes. Coordinates of scalp cells in the basis of the first 10 PCs obtained in Supplemental Experimental Methods: Principal component analysis were used as input to the Slingshot algorithm. We specified the basal1 cluster as the starting state, and the granular cluster as a terminal state. We then used the pseudotime prediction to order all cells in all three scalp samples along the predicted differentiation trajectory to each terminal cluster.

tSNE mapping
We used Rtsne (Krijthe, 2015; Maaten, 2008) (version 0.13) to perform T-distributed stochastic neighbor embedding (tSNE) on the 20-dimensional representation of cells described in Experimental Procedures: Principal component analysis. tSNE used parameters theta = .5 and perplexity = 40.

Processing time and sex-specific bias analysis
To assess for possible confounding effects of processing time and male/female gene expression differences, we compared 4 samples to our 9 core normal epidermis samples (abd4, br41epi, br53epi, fore12, fore8, fore9, s11, scalp26, and scalp32). Two additional trunk samples were processed in less than 2 hours (vs. median of 22 hours for samples in the 9 core samples), in order to control for variances in processing time. Two additional male trunk samples were also collected, rather than the female trunk samples in our core series. These 4 samples were normalized and imputed together with cells from our 9 core normal samples. The additional 4 samples were then projected onto the 11 clusters identified from the core 9 samples using the same PCA-based nearest neighbor method and settings (scikit-learn's KNeighborsClassifier) used to map the psoriasis samples.

The silhouette statistic (Rousseeuw, 1987) was then used to assess whether cells in these four new samples clustered differently from our original samples. The silhouette statistic compares the average principal component distance between a cell and A) other cells of its same group vs. B) cells of any other group. Tissue type and cluster represent types of groups. Lower average distance within cells of the same group, relative to cells of the closest other group, corresponds to a positive silhouette, with a maximum of 1. Higher average distance within cells of the same group, relative to cells of the closest other group, corresponds to a negative silhouette, with a minimum of -1 (and indicates that a cell would fit better in the other group). A silhouette near 0 means that a cell has a similar average distance to two groups. Averaging the silhouette statistic across cells can give an overall sense of whether the assessed groupings represents tight, well separated sets (average silhouette approaching 1) or heterogeneous groups where cells don't clearly fall within a single group (average silhouette around 0).

In column 2 of the table below (labelled tissue), we show the silhouette statistic calculated on the tissue groups within each cluster, averaged across all the cells of each tissue**.** Here, the silhouette statistic shows that in clusters, cells demonstrate similar distance between other cells in their tissue of origin and the next closest tissue (averaged values are neither close to 1 or -1 but close to 0). This indicates that cells in a given cluster are not highly related by tissue type. For the 2-hour and male samples, this indicates that time or sex-specific transcriptional signal causes relatively minor confounding segregation, even within discrete clusters.

In column 3 of the table below (labelled cluster), we show the silhouette statistic calculated on the cluster groups within each tissue, averaged across the cells of each tissue. The relatively high values across this analysis show that cells of a given cluster are tightly related, regardless of tissue source. The 2-hour and male samples show similar average silhouettes to the originally analyzed tissue types, indicating that any time or sex-specific transcriptional signal does not substantially impair how robustly the clusters segregate cells.

	tissue	cluster
2 hour	-0.08617	0.374327
male	-0.08449	0.382997
foreskin	0.075526	0.297912
trunk	0.031249	0.408574
scalp	-0.01125	0.335295

Supplemental References

Krijthe, J.H. (2015). Rtsne: T-Distributed Stochastic Neighbor Embedding using a Barnes-Hut Implementation.

Maaten, L. (2008). Visualizing data using t-SNE. The Journal of Machine Learning Research *9*, 2579.

Macosko, E.Z., Basu, A., Satija, R., Nemesh, J., Shekhar, K., Goldman, M., Tirosh, I., Bialas, A.R., Kamitaki, N., Martersteck, E.M., *et al.* (2015). Highly Parallel Genome-wide Expression Profiling of Individual Cells Using Nanoliter Droplets. Cell *161*, 1202-1214.

Pedregosa, F., Varoquaux, G., Gramfort, A., Michel, V., Thirion, B., Grisel, O., Blondel, M., Prettenhofer, P., Weiss, R., Dubourg, V., *et al.* (2011). Scikit-learn: Machine Learning in Python. J Mach Learn Res *12*, 2825-2830.

Risso, D., Perraudeau, F., Gribkova, S., Dudoit, S., and Vert, J.P. (2018). A general and flexible method for signal extraction from single-cell RNA-seq data. Nat Commun *9*, 284.

Rousseeuw, P.J. (1987). Silhouettes: A graphical aid to the interpretation and validation of cluster analysis. Journal of Computational and Applied Mathematics *20*, 53-65.

Shi, J.B., and Malik, J. (2000). Normalized cuts and image segmentation. Ieee T Pattern Anal *22*, 888-905.

Street, K., Risso, D., Fletcher, R.B., Das, D., Ngai, J., Yosef, N., Purdom, E., and Dudoit, S. (2018). Slingshot: cell lineage and pseudotime inference for single-cell transcriptomics. BMC Genomics *19*, 477.

van Dijk, D., Sharma, R., Nainys, J., Yim, K., Kathail, P., Carr, A.J., Burdziak, C., Moon, K.R., Chaffer, C.L., Pattabiraman, D., *et al.* (2018). Recovering Gene Interactions from Single-Cell Data Using Data Diffusion. Cell *174*, 716-729 e727.

von Luxburg, U. (2007). A tutorial on spectral clustering. Statistics and Computing *17*, 395-416.

Yan, D.H., Huang, L., and Jordan, M.I. (2009). Fast Approximate Spectral Clustering. Kdd-09: 15th Acm Sigkdd Conference on Knowledge Discovery and Data Mining, 907-915.

Supplemental Data

Data S1 Per sample representation and marker gene expression in t-SNE plot epidermal clusters, Related to Figure 1, 4, and 6

(A) Trunk-specific tSNE plot (as in Figure 1A) pseudocolored by sample. (B) Foreskin-specific tSNE plot (as in Figure 1A) pseudocolored by sample. (C) Scalp-specific tSNE plot (as in Figure 1A) pseudocolored by sample. (D) 15 scalp-specific cluster tSNE plot (as in Figure 4) pseudocolored by sample. (E). Immune cluster tSNE plot (as in Figure 6) pseudocolored by sample. (F) tSNE plot incorporating all 9 normal samples (G) Expression pattern of epidermal subpopulation marker gene expression across cell clusters in anatomic site-specific tSNE plots.

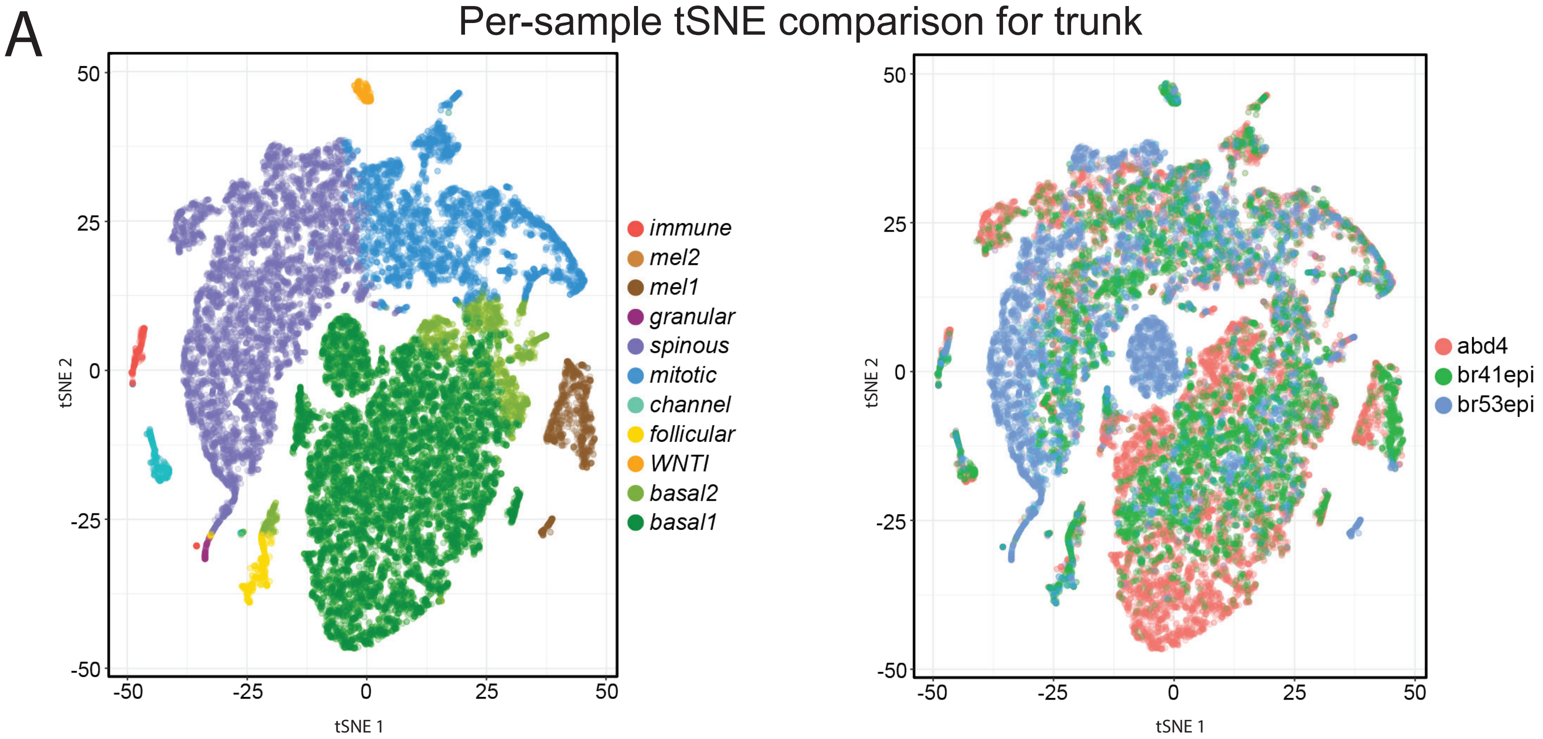

Color-coded by cluster (Fig 1A)

Color-coded by sample

B

Per-sample tSNE comparison for foreskin

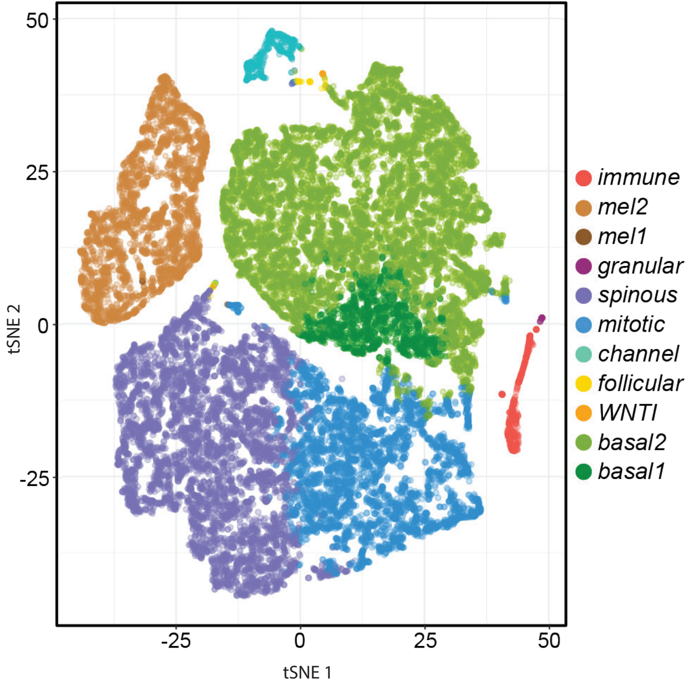

Color-coded by cluster (Fig 1A)

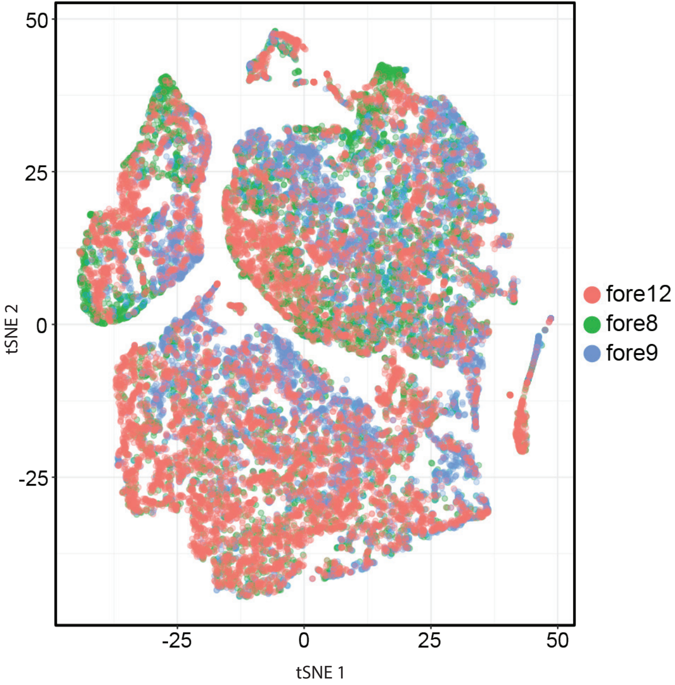

Color-coded by sample

C

Per-sample tSNE comparison for scalp

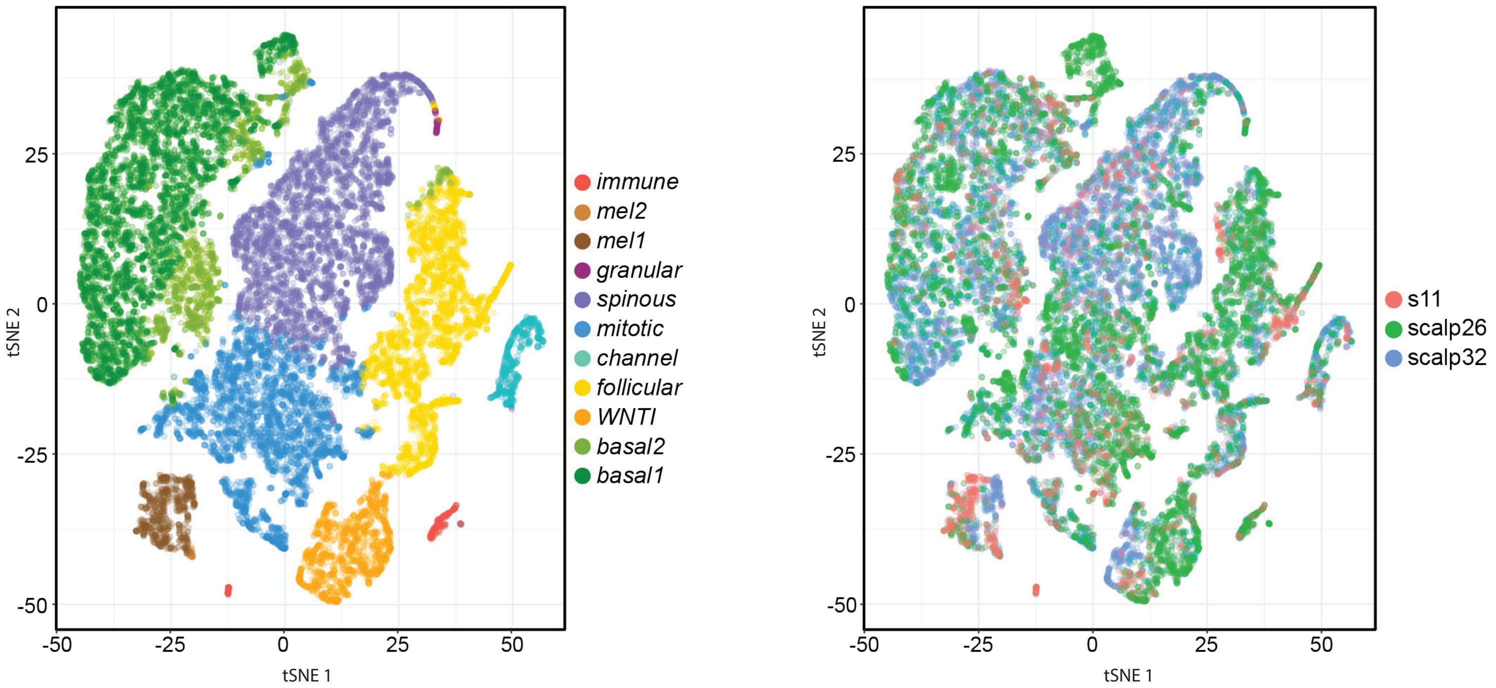

Color-coded by cluster (Fig 1A)

Color-coded by sample

D

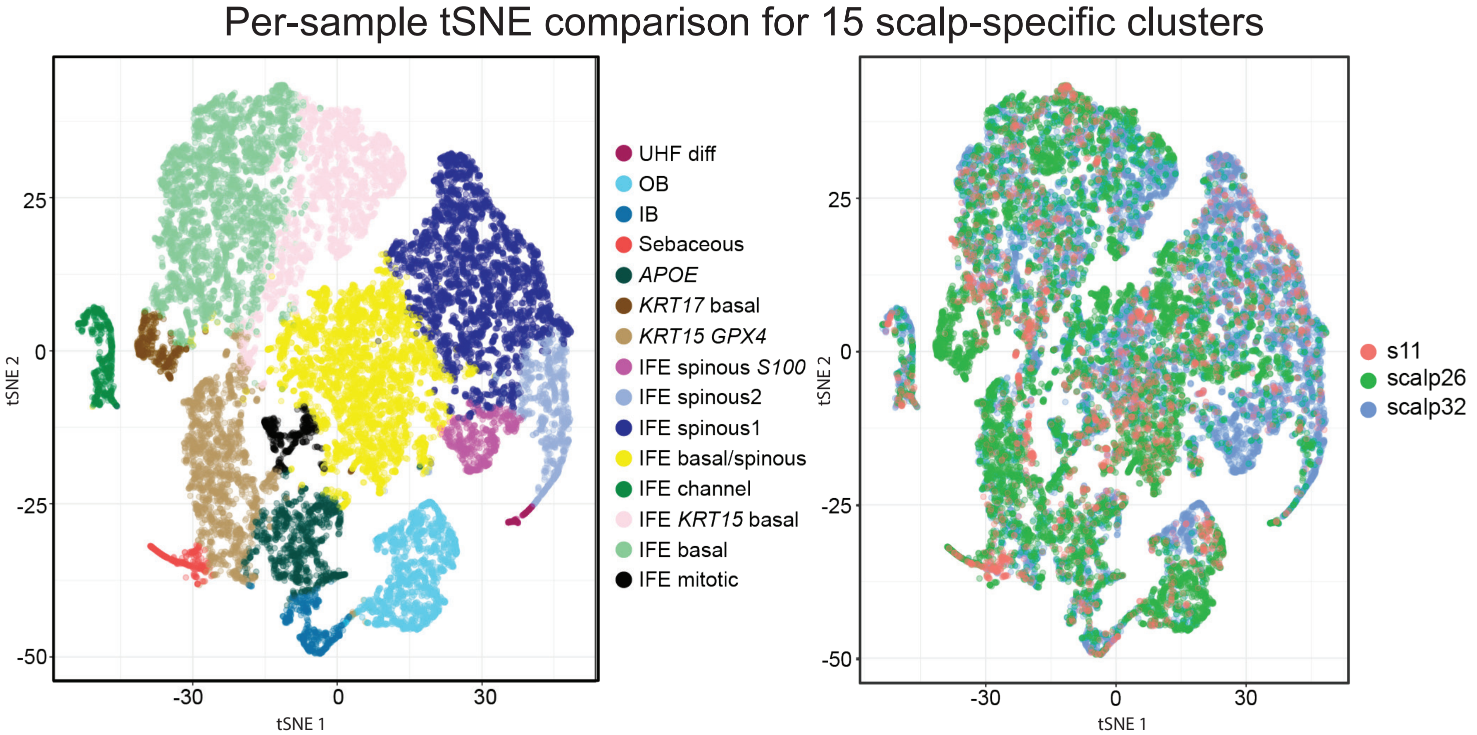

Color-coded by cluster (Fig 3)

Color-coded by sample

E

Per-sample tSNE comparison for immune clusters

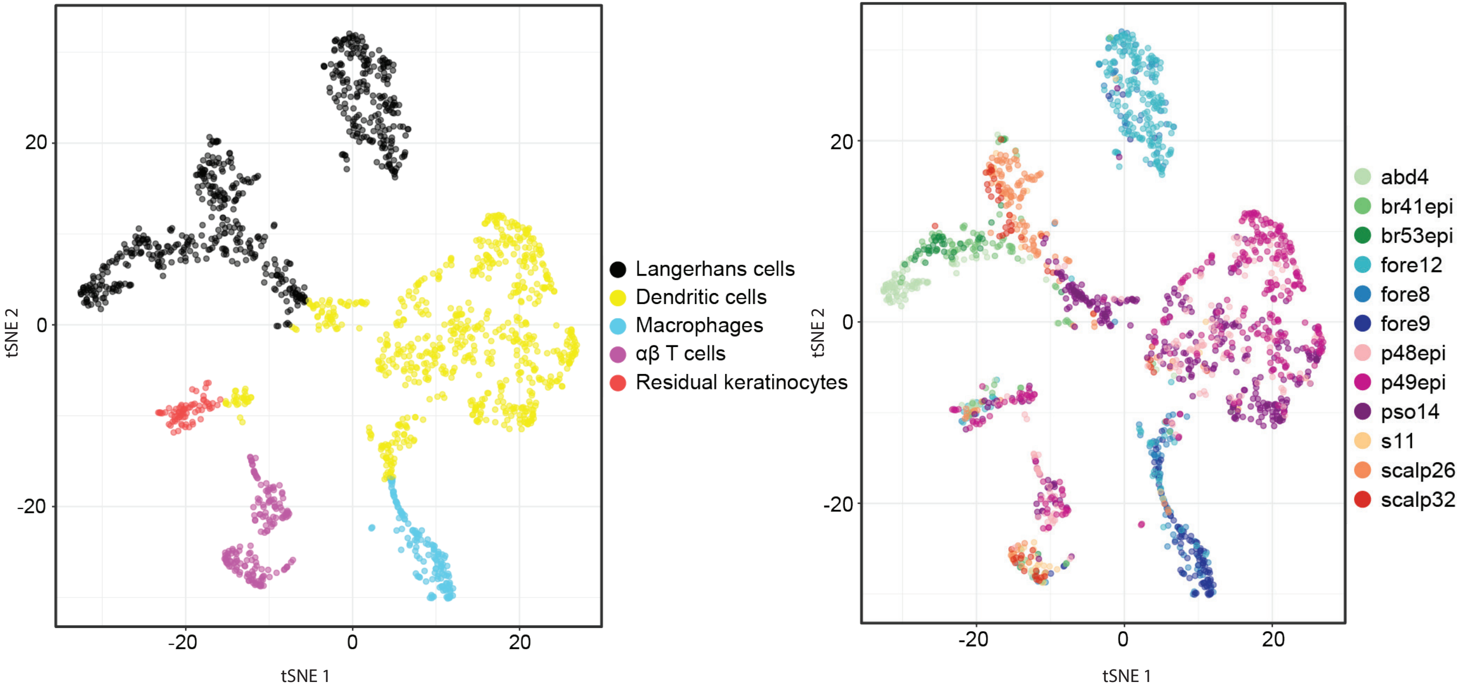

Color-coded by cluster (Fig 6)

Color-coded by sample

F

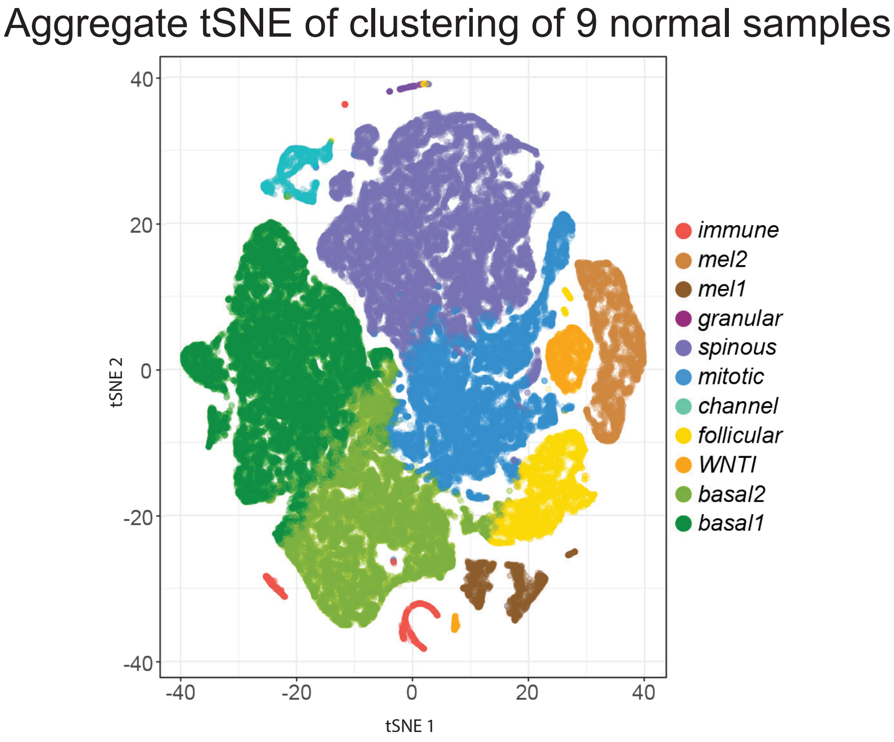
Aggregate tSNE of clustering of 9 normal samples
40
20
0
-20
-40
tSNE 2
-40
-20
0
20
40
tSNE 1
immune
mel2
mel1
granular
spinous
mitotic
channel
follicular
WNTI
basal2
basal1

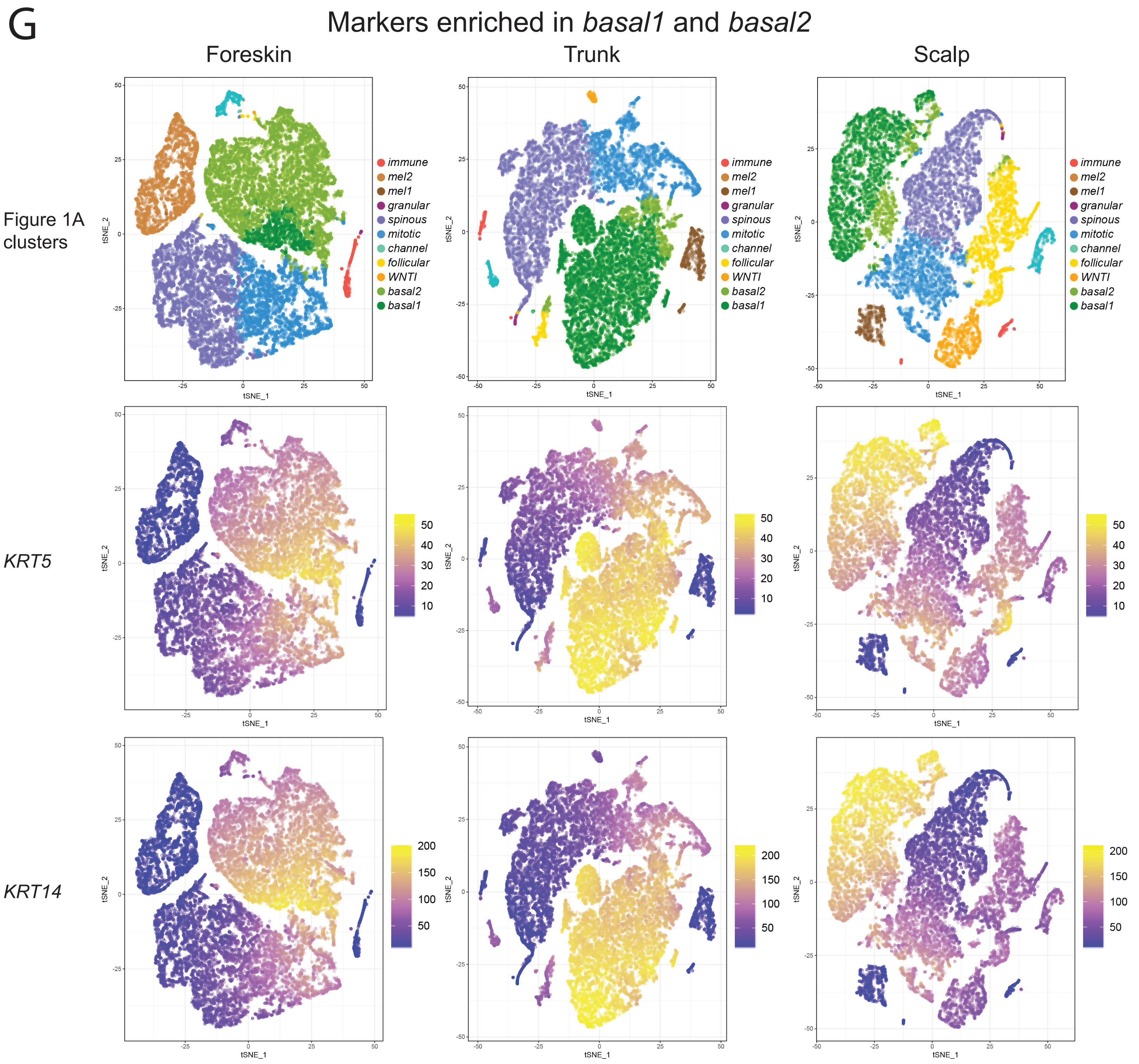
G
Markers enriched in basal1 and basal2
Foreskin
Trunk
Scalp
Figure 1A clusters
KRT5
KRT14
immune
mel2
mel1
granular
spinous
mitotic
channel
follicular
WNTI
basal2
basal1
tSNE_1
tSNE_2
50
40
30
20
10
200
150
100
50

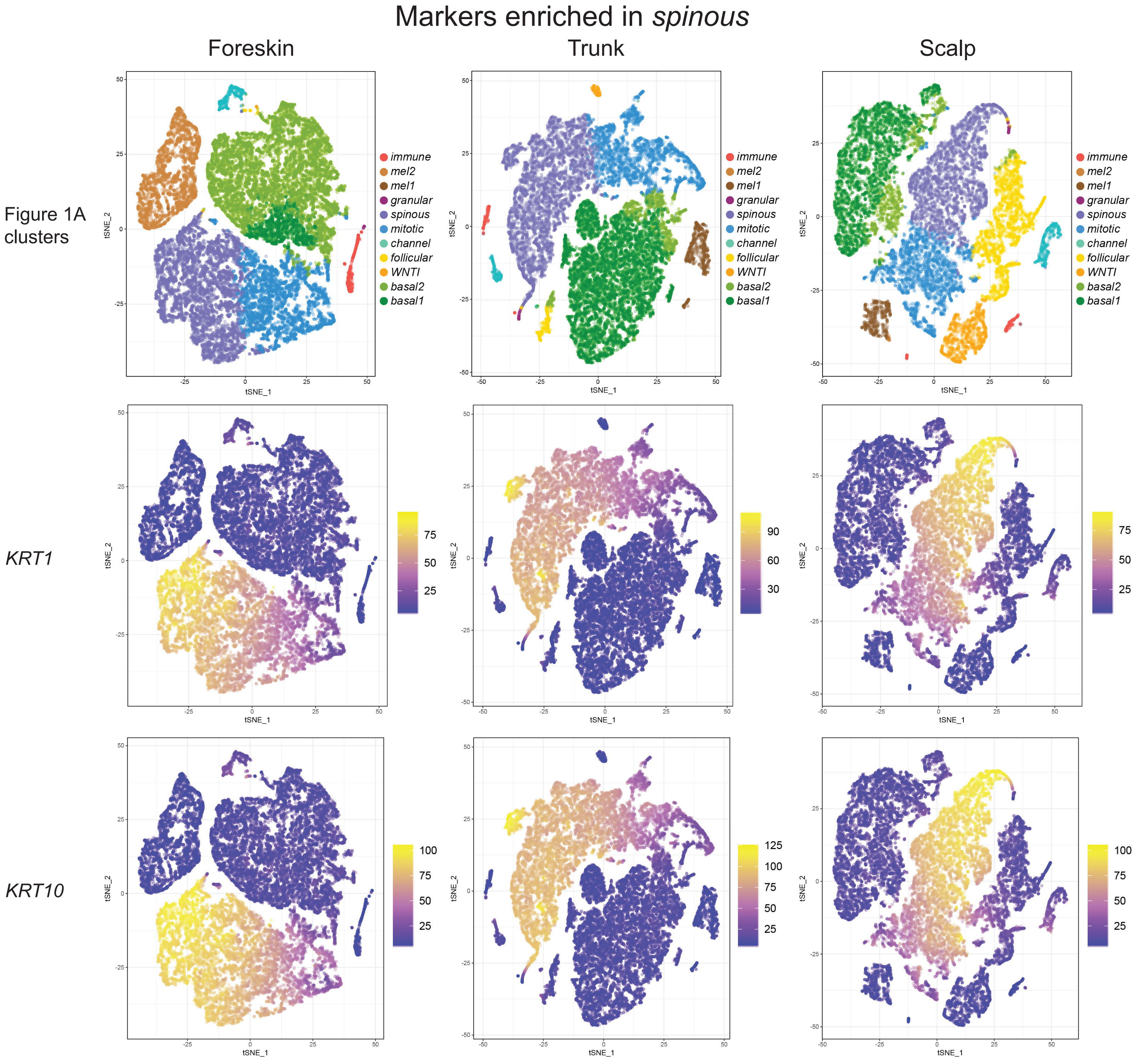
Markers enriched in spinous
Foreskin
Trunk
Scalp
Figure 1A clusters
KRT1
KRT10
immune
mel2
mel1
granular
spinous
mitotic
channel
follicular
WNTI
basal2
basal1
tSNE_1
tSNE_2

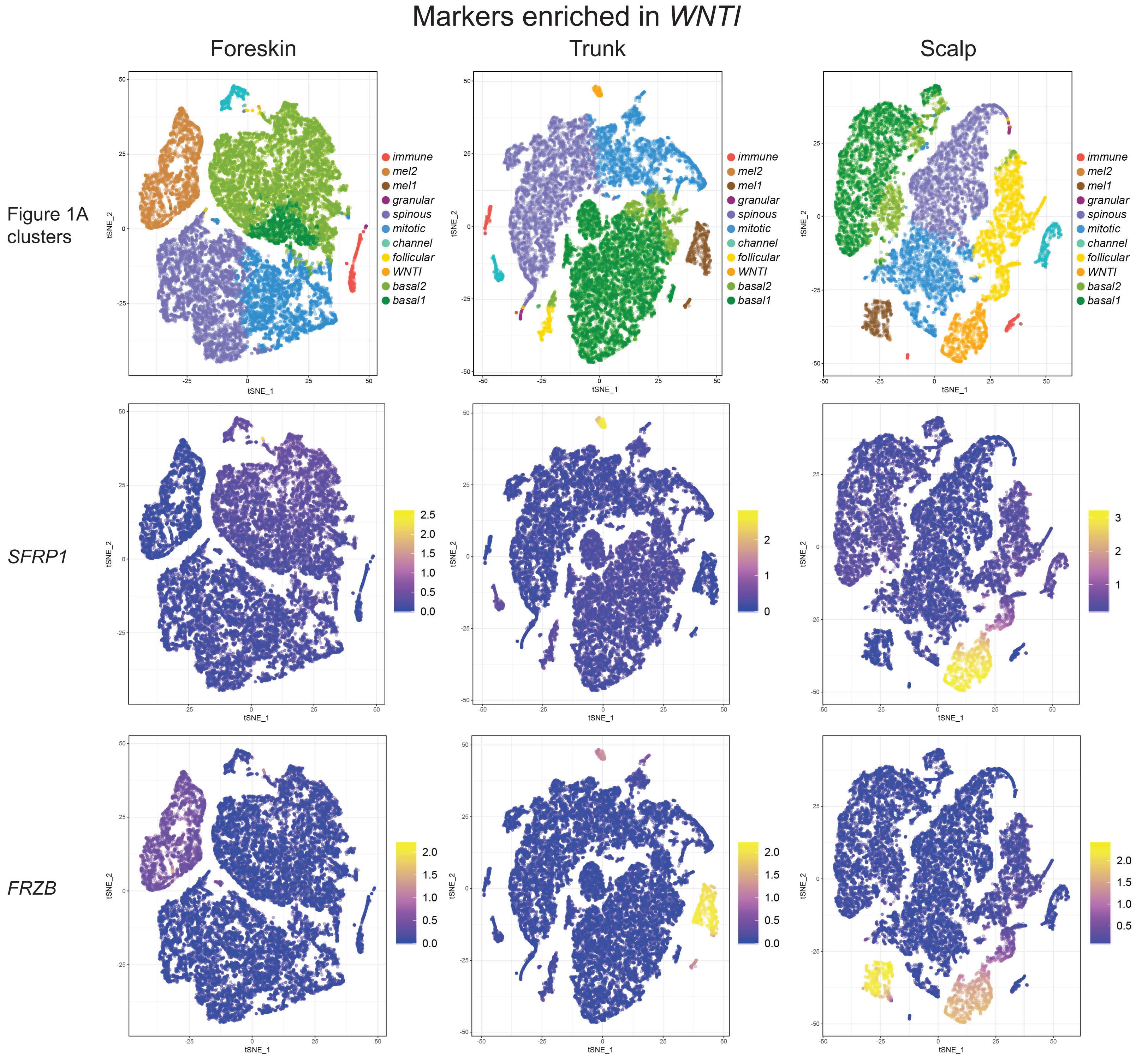
Markers enriched in WNTI
Foreskin
Trunk
Scalp
Figure 1A clusters
SFRP1
FRZB
immune
mel2
mel1
granular
spinous
mitotic
channel
follicular
WNTI
basal2
basal1
tSNE_1
tSNE_2

Markers enriched in *follicular*

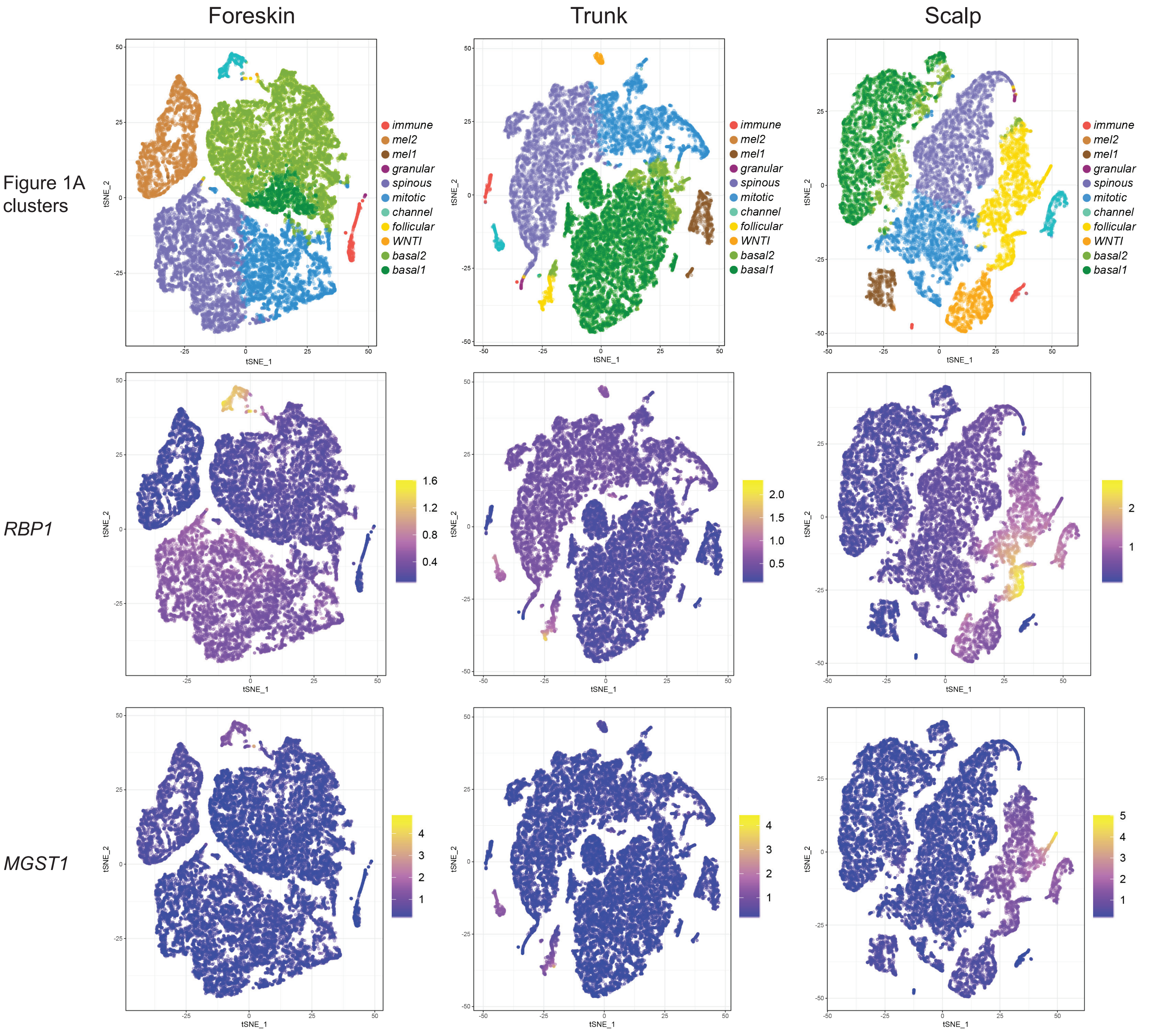

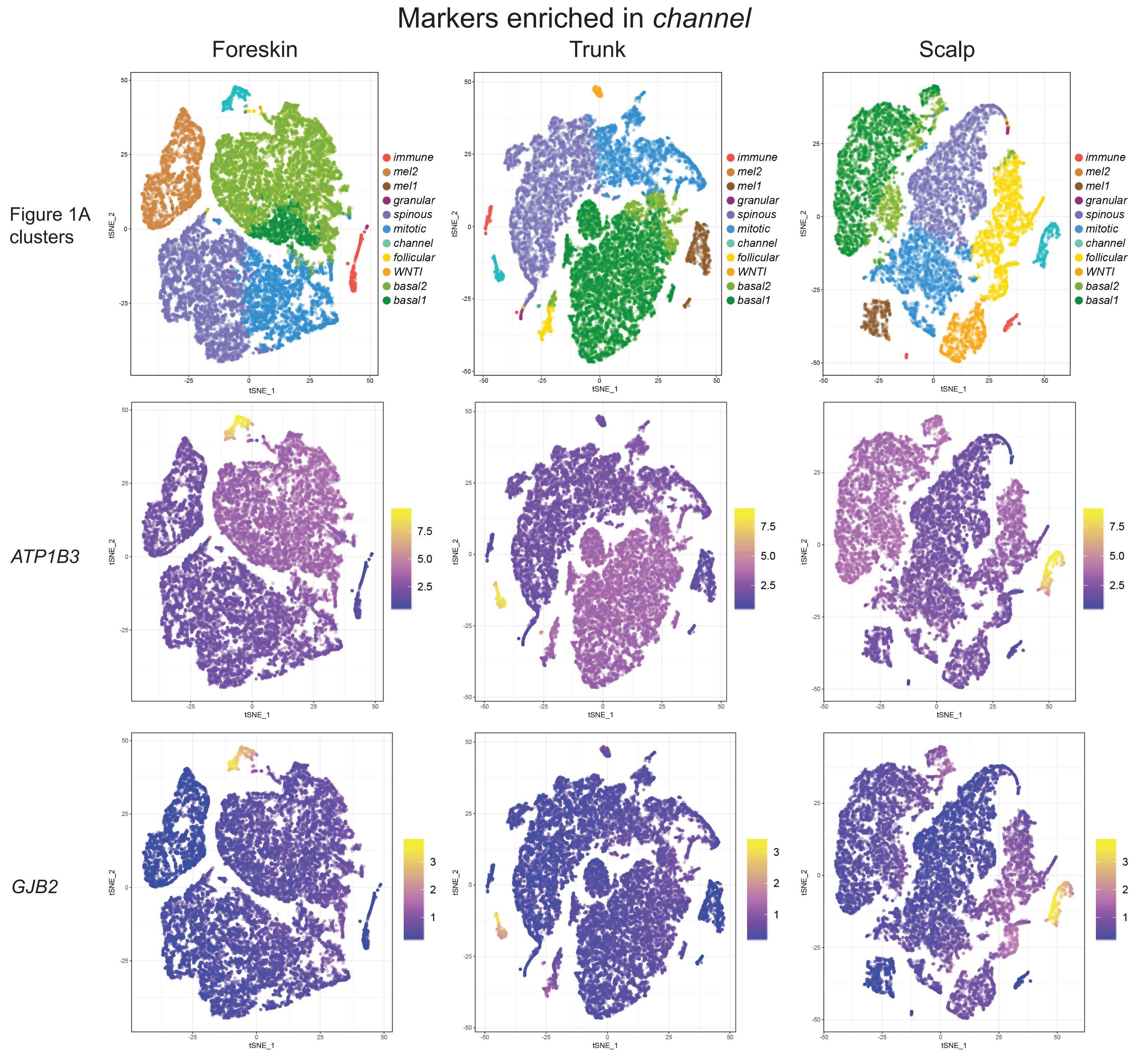
Markers enriched in channel
Foreskin
Trunk
Scalp
Figure 1A clusters
ATP1B3
GJB2
immune
mel2
mel1
granular
spinous
mitotic
channel
follicular
WNTI
basal2
basal1
tSNE_1
tSNE_2

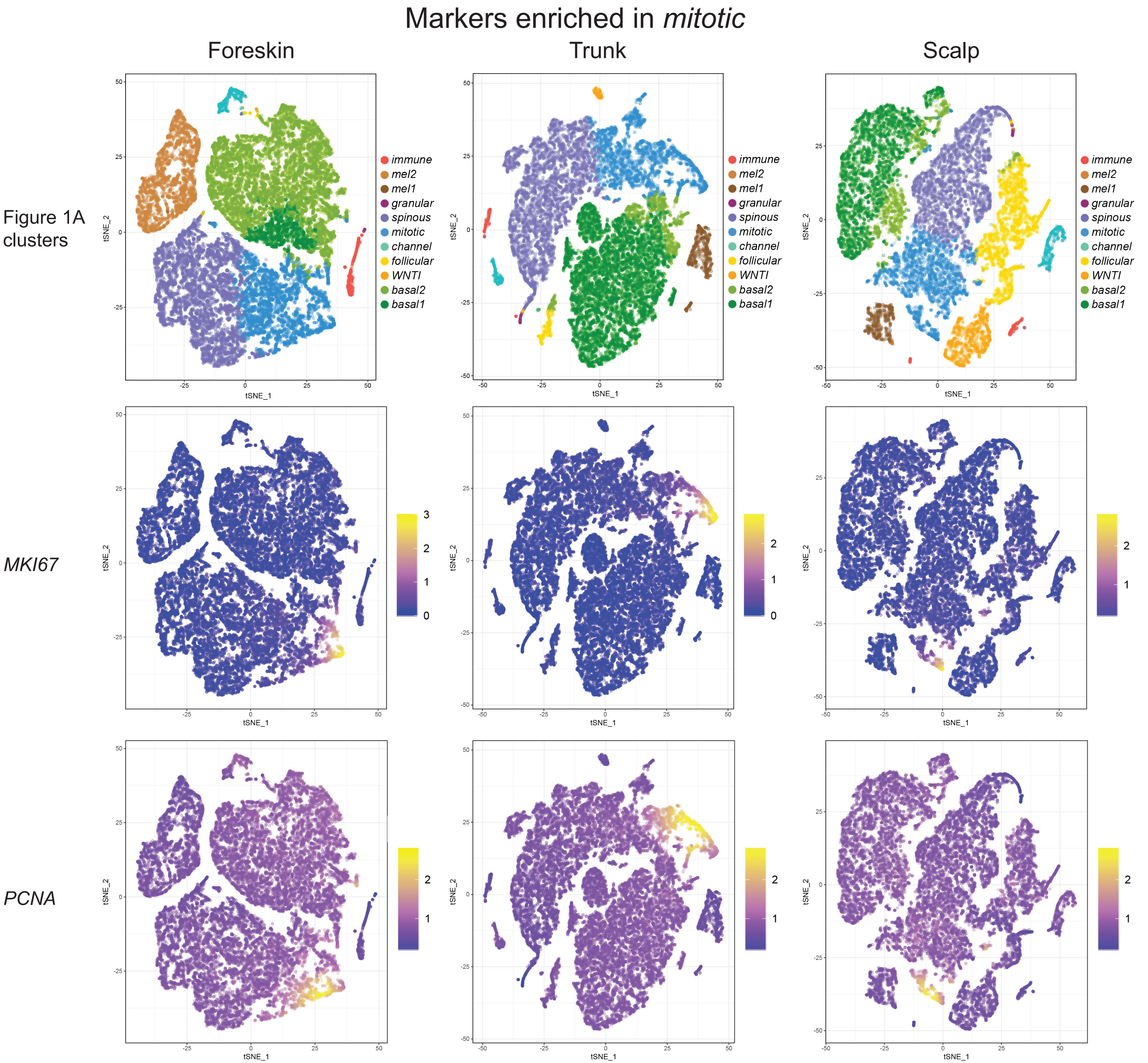

Markers enriched in *mitotic*
Foreskin
Trunk
Scalp
Figure 1A clusters
MKI67
PCNA
immune
mel2
mel1
granular
spinous
mitotic
channel
follicular
WNTI
basal2
basal1
tSNE_1
tSNE_2

Markers enriched in *granular*

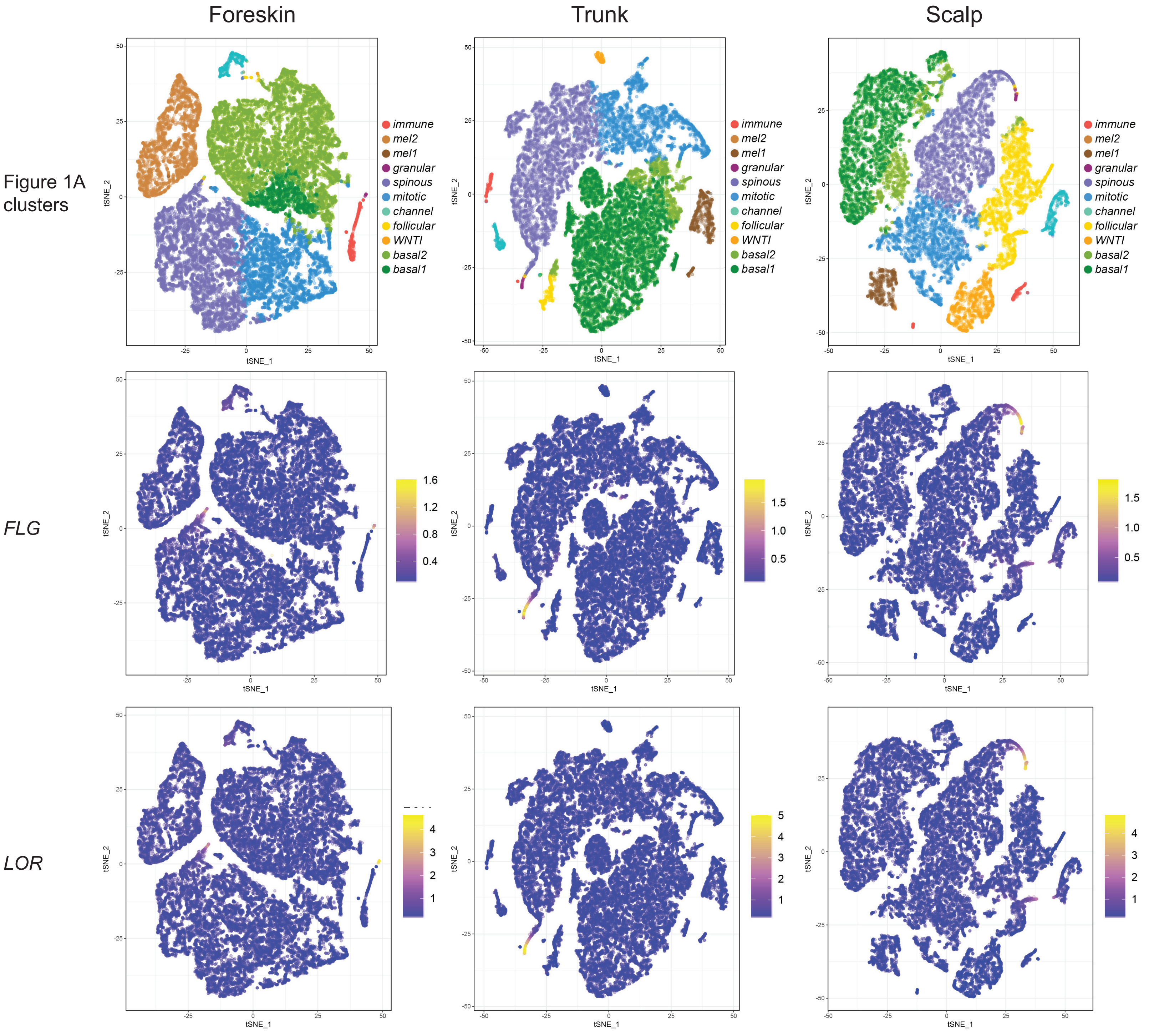

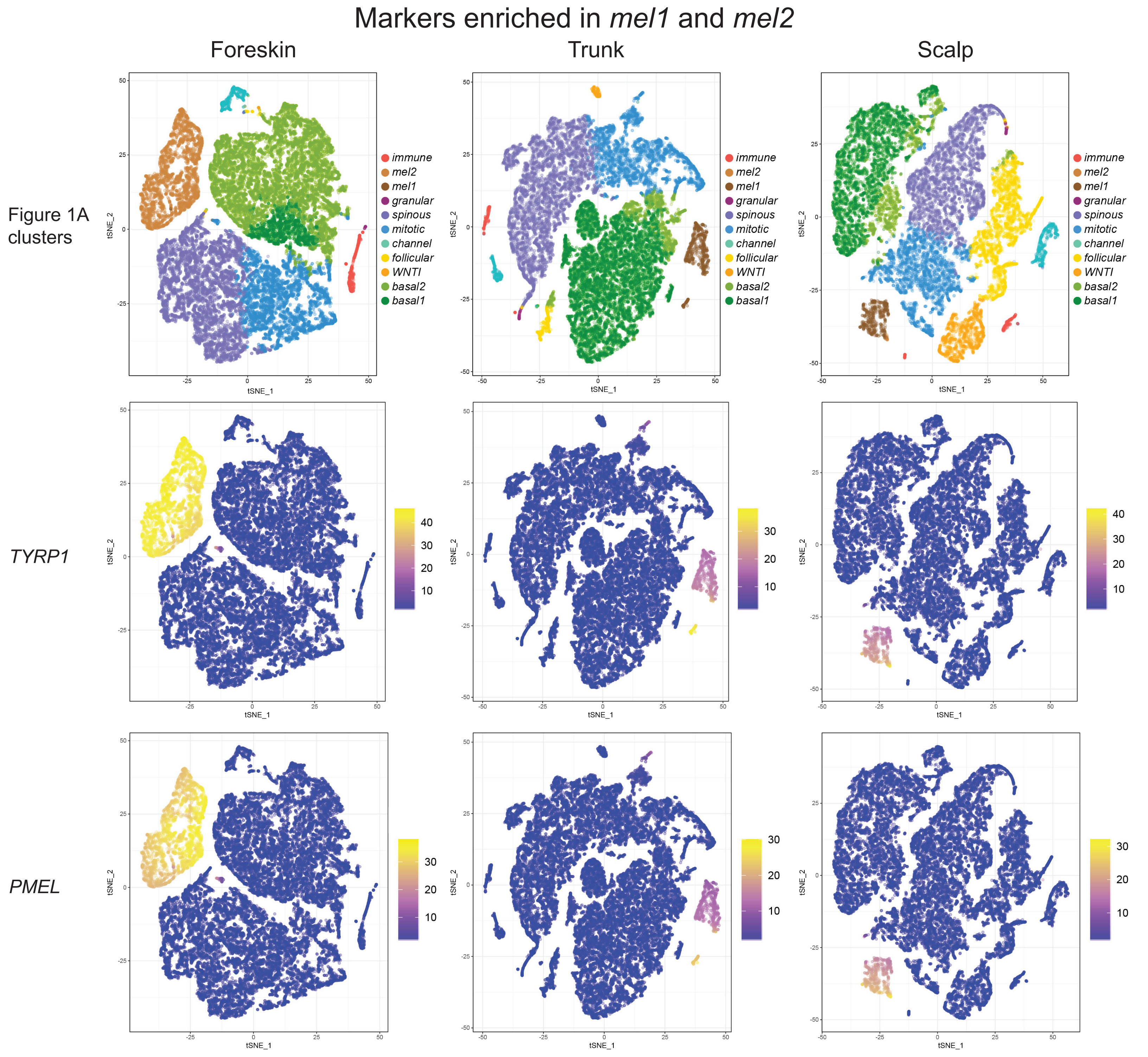
Markers enriched in mel1 and mel2
Foreskin
Trunk
Scalp
Figure 1A clusters
TYRP1
PMEL
immune
mel2
mel1
granular
spinous
mitotic
channel
follicular
WNTI
basal2
basal1
tSNE_1
tSNE_2

Markers enriched in *immune*

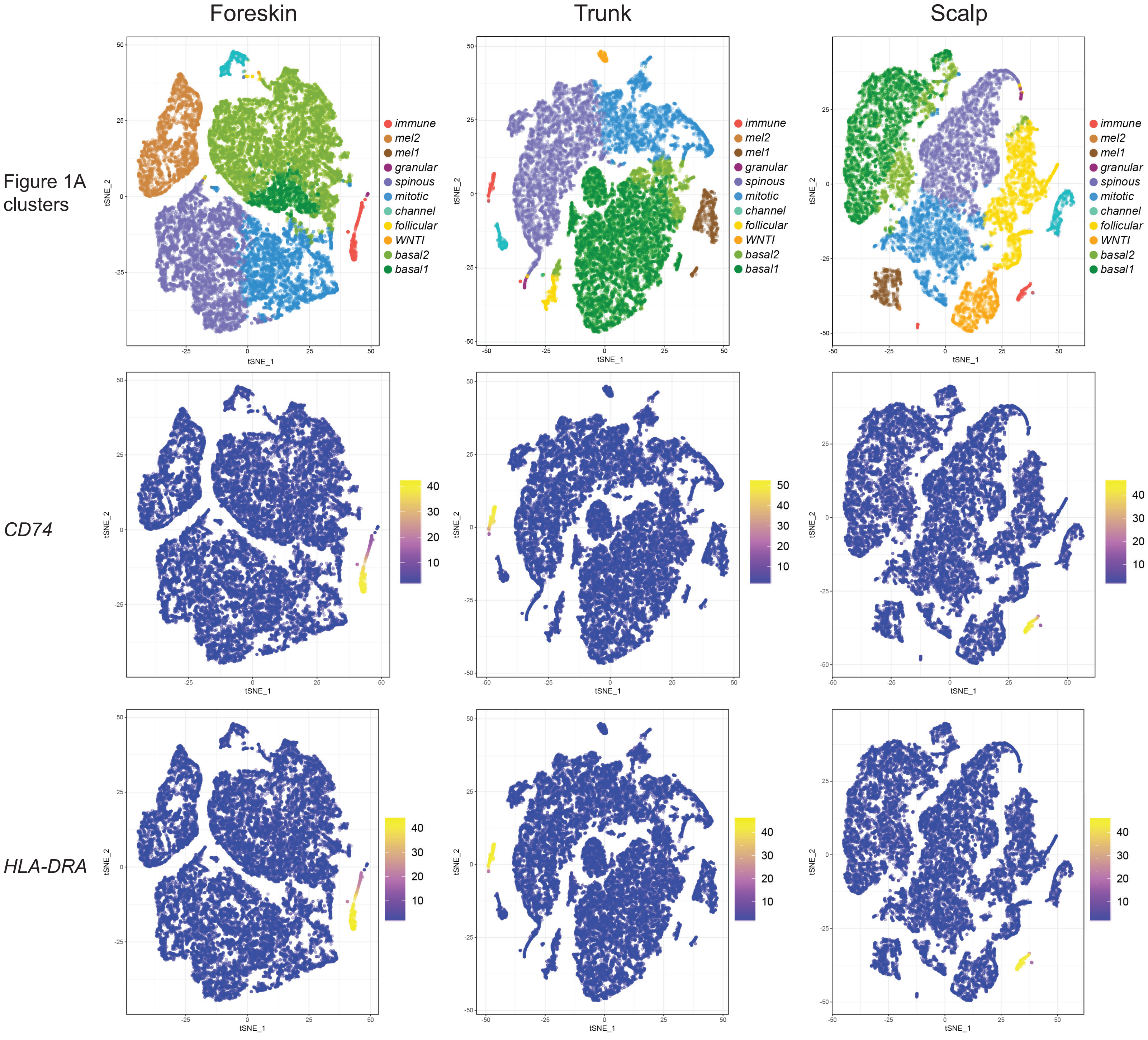

Supplement: 2 [file NIHMS1516330-supplement-2.pdf]
